# Cancer care coordinators in stage III colon cancer: a cost-utility analysis

**DOI:** 10.1186/s12913-015-0970-5

**Published:** 2015-08-05

**Authors:** Tony Blakely, Lucie Collinson, Giorgi Kvizhinadze, Nisha Nair, Rachel Foster, Elizabeth Dennett, Diana Sarfati

**Affiliations:** Burden of Disease Epidemiology, Equity, and Cost-Effectiveness (BODE3) Programme, Department of Public Health, University of Otago Wellington, PO Box 7343, Wellington, New Zealand; Department of Surgery, University of Otago Wellington, PO Box 7343, Wellington, New Zealand

**Keywords:** Care coordinators, Patient navigators, Economic evaluation, Cost-effectiveness, Cost-utility analysis, Colon cancer

## Abstract

**Background:**

There is momentum internationally to improve coordination of complex care pathways. Robust evaluations of such interventions are scarce. This paper evaluates the cost-utility of cancer care coordinators for stage III colon cancer patients, who generally require surgery followed by chemotherapy.

**Methods:**

We compared a hospital-based nurse cancer care coordinator (CCC) with ‘business-as-usual’ (no dedicated coordination service) in stage III colon cancer patients in New Zealand. A discrete event microsimulation model was constructed to estimate quality-adjusted life-years (QALYs) and costs from a health system perspective. We used New Zealand data on colon cancer incidence, survival, and mortality as baseline input parameters for the model. We specified intervention input parameters using available literature and expert estimates. For example, that a CCC would improve the coverage of chemotherapy by 33 % (ranging from 9 to 65 %), reduce the time to surgery by 20 % (3 to 48 %), reduce the time to chemotherapy by 20 % (3 to 48 %), and reduce patient anxiety (reduction in disability weight of 33 %, ranging from 0 to 55 %).

**Results:**

Much of the direct cost of a nurse CCC was balanced by savings in business-as-usual care coordination. Much of the health gain was through increased coverage of chemotherapy with a CCC (especially older patients), and reduced time to chemotherapy. Compared to ‘business-as-usual’, the cost per QALY of the CCC programme was $NZ 18,900 (≈ $US 15,600; 95 % UI: $NZ 13,400 to 24,600). By age, the CCC intervention was more cost-effective for colon cancer patients < 65 years ($NZ 9,400 per QALY). By ethnicity, the health gains were larger for Māori, but so too were the costs, meaning the cost-effectiveness was roughly comparable between ethnic groups.

**Conclusions:**

Such a nurse-led CCC intervention in New Zealand has acceptable cost-effectiveness for stage III colon cancer, meaning it probably merits funding. Each CCC programme will differ in its likely health gains and costs, making generalisation from this evaluation to other CCC interventions difficult. However, this evaluation suggests that CCC interventions that increase coverage of, and reduce time to, effective treatments may be cost-effective.

**Electronic supplementary material:**

The online version of this article (doi:10.1186/s12913-015-0970-5) contains supplementary material, which is available to authorized users.

## Background

Cancer care can be complex for patients to navigate. Partly in response to this, there has been an increasing emphasis on cancer care coordinator roles (CCC; also known as patient navigators, key workers, one to one support workers, liaison officers, coordination officers, and case management nurses) to improve patient outcomes within cancer care services, especially among lower socio-economic populations [1, 2]. Providing better coordinated care for cancer patients has been identified by the National Institute for Health and Care Excellence (NICE) as essential to enabling the needs of patients to be identified and met [3]. CCC roles vary in terms of the stage of cancer management on which they focus, ranging from access to cancer screening, to coordination of care following diagnosis, to survivorship support. Evidence of effectiveness is starting to emerge with regards to improving uptake of cancer screening, earlier stage at diagnosis, timeliness of care, adherence to treatment, hospital utilisation and patient satisfaction [4–13].

Expenditure on cancer care is increasing at an alarming rate worldwide. This increase highlights the need for changes to models of care delivery and a need for evaluations to assist with determining and prioritising cost-effective interventions – including service configurations in addition to pharmaceuticals and discrete treatments [14]. There are examples of economic analyses of CCC type interventions conducted elsewhere [15–17], but to our knowledge no nurse-led hospital-based cancer coordination intervention has been subjected to a rigorous cost-utility analysis [18]. There is considerable uncertainty in the effect and cost of service-level interventions. However, decision-makers still need to prioritise where cancer control resources are deployed. This paper brings together these agendas, using microsimulation modelling incorporating the considerable uncertainties.

Given the diverse nature of CCC programmes, it was not possible to evaluate them generally across all cancer sites. We thus focused on stage III colon cancer for three reasons. First, colon cancer is a ‘priority’ cancer for which CCC programmes are emerging; second, the treatment pathway requires both surgery and chemotherapy and therefore more coordination may be required than (say) early stage colon cancer for which surgery alone is considered curative; and third, there is a consensus that adjuvant chemotherapy should be offered following surgery for stage III disease.

There is increasing concern about social inequalities in cancer survival and outcomes, and New Zealand is no exception. For example, Māori (the indigenous population) have worse survival from cancer that is not fully explained by stage at presentation [19], including for colon cancer [20]. Part of the reason for these social inequalities in survival is likely to include differences in receipt of treatments and waiting times to treatments [21], which may be remedied through the CCC programmes.

The objective of this paper is to determine the cost-utility of a CCC intervention in stage III colon cancer, including differences by patient age, sex, ethnicity, and socio-economic status (collectively called socio-demographics from here on). The CCC in question is a clinical nurse specialist who provides support and information to the patient, coordinates the provision of treatment, and identifies and addresses barriers to care.

## Methods

Methods are briefly outlined here; more detail is provided in the Additional files. A health system perspective was used; costs and benefits beyond the health system (e.g. productivity costs) were out of scope. Participants were patients with stage III colon cancer in New Zealand, modelled till death, or age 110 years. Costs were in 2011 New Zealand dollars (with conversion of the main incremental cost-effectiveness ratio to US dollars and UK pounds using exchange rates as of March 2013). A 3 % per annum discount rate was applied to costs and benefits.

### Intervention and comparator definition

The CCC intervention was defined as a hospital-based clinical nurse specialist (CNS) who is the main point of contact for the patient and a key point of contact for health professionals involved in the patient’s care. This begins at the point of provisional diagnosis of colon cancer and continues until initiation of chemotherapy for patients with confirmed stage III colon cancer. The CCC role would include: providing information and support for the patient, identifying and addressing patient barriers to accessing care (transport/financial/social), coordinating arrangements for pre-operative assessments and hospital admission, optimising post-operative care, tracking investigations and appointments, ensuring the patient is discussed at a multidisciplinary team meeting, making referrals as necessary, and acting on any administrative delays. (See Additional file 1 for more detail on the CCC intervention).

We specified that the health gain from a CCC intervention would be via four effects: reducing time from provisional diagnosis to surgery (and associated improved survival), reducing time from surgery to chemotherapy (and associated improved survival), improving the coverage of chemotherapy and associated improved survival (there was little room for improvement in coverage of surgery) and reducing patients’ anxiety during diagnosis and treatment (thus improving patients’ quality of life). How these effects were quantified is described in the Input Parameter section below and in Additional file 2. We acknowledge that treatment coverage and waiting times are also influenced by other resource constraints (e.g. theatre space/time, staffing, and ward space), and a CCC per se will have little or no impact on these structural barriers. It should also be noted that this intervention was conceptualised based on the New Zealand context.

The comparator was business-as-usual i.e. no dedicated CCC programme as is common in many countries. In a business-as-usual scenario, needs assessment is provided by a number of different nurses, doctors or other health professionals at various points along the cancer care pathway, followed by referrals to other health services. No one individual is responsible for tracking referrals, investigations or appointments and acting on delays, with potential for tasks to be duplicated or missed. (See Additional file 1 for more detail on the comparator).

### Model overview

**Fig. 1:**
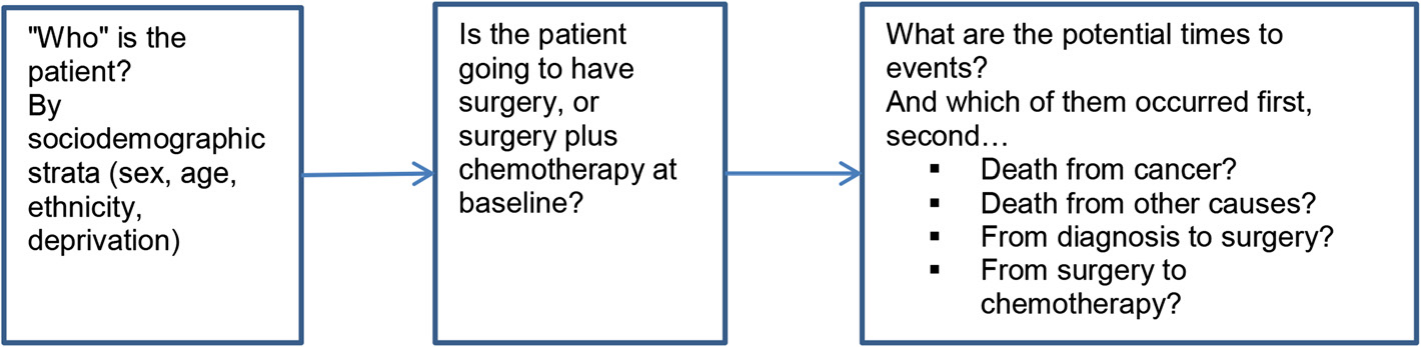
Discrete event simulation (DES) model structure

We constructed a discrete event simulation model (DES; a form of microsimulation) to address our research question. Modelling was conducted in Tree Age Pro 2012. A DES model was chosen as it allows “jumping” from the time of one ‘event’ to the time of the next event, making it particularly useful when patients are subject to competing events. There were four competing events in our case: time to death from colon cancer, time to death from other causes, time to surgery, and time to start of chemotherapy. The first three ‘compete’ at diagnosis. If the patient makes it to surgery, then deaths from cancer, death from other causes and time to chemotherapy are the three competing events. And if the patient makes it to chemotherapy, the two (absorbing) death states are the remaining competing events (see Figure 1 for model structure overview). The main model outputs were health gain (in quality-adjusted life-years or QALYs), incremental costs, and incremental cost-effectiveness ratios (ICERs).

### Input parameters

Selected input parameters are shown in Tables 1 and 2, and summarised in the text below. A full input parameter table and further explanation is provided in Additional file 2. Most input parameters have a best estimate (‘expected value’). To capture the lack of perfect knowledge around each input parameter (input parameter uncertainty), a probability distribution around the expected value was usually defined.

**Table 1:** Selected effect size parameters used in the model (greater detail and full list provided in Additional file 2)

Variable name	Variable definition	Source, derivation and application	Expected value and 95 % uncertainty interval
*Effect of a CCC on increasing receipt of chemotherapy*			
*Proportion surgery only at baseline → surgery and chemotherapy*	Proportion shifted from receiving surgery only to surgery plus chemotherapy	Goodwin et al. 2003 [25] and expert estimates	0.33 (0.09 to 0.65)
		See Additional file 2	Beta distribution
*Hazard ratio (HR) for chemotherapy*	Effect of chemotherapy with oxaliplatin on breast cancer mortality	Sargent et al. 2009 [36]	1: 0.72 (0.61 to 0.85)
		De Gramont et al. 2007 [37]	2: 0.78 (0.63 to 0.98)
		Andre et al. 2004 [38]	Log normal distribution
	(Effect of chemotherapy without oxaliplatin on breast cancer mortality considered as scenario analysis)	See Additional file 2	
		Product of two HRs: 1: effect of chemo without oxaliplatin compared to no chemo multiplied by 2: effect of chemo with oxaliplatin compared to without oxaliplatin	
*Effect of a CCC on reducing wait times to treatments*			
*Reduction in days to surgery*	Proportionate reduction in days to surgery due to a CCC	Haideri et al. 2011 [10] and expert estimates	0.20 (0.03 to 0.48)
		See Additional file 2	Beta distribution
*Reduction in EMR per day decrease in time from diagnosis to surgery*	Reduction in cancer excess mortality rate (EMR) per day decrease in time from diagnosis to surgery (i.e. the effect of getting surgery faster on colon cancer mortality)	No direct evidence. Estimated using protocol, [39] Whyte et al. 2011, [40] Tappenden et al. 2007 [41]	0.9972 ratio decrease in EMR per day quicker to surgery (0.9955 to 0.9987)
			Log normal distribution
		See Additional file 2	
*Reduction in days to chemotherapy*	Proportionate reduction in average days from surgery to chemotherapy due to a CCC	Expert estimates	0.20 (0.03 to 0.48)
		See Additional file 2	Beta distribution
*Reduction in EMR per day decrease in time from surgery to chemotherapy*	Reduction in cancer excess mortality rate (EMR) per day decrease in time from surgery to chemotherapy (i.e., the effect of getting chemotherapy faster on colon cancer mortality)	Biagi et al. 2011 [42]	0.9953 ratio decrease in EMR per day quicker to chemotherapy (0.9983 to 0.9969)
		See Additional file 2	
			Log normal distribution
*Effect of a CCC on reducing colon cancer morbidity*			
*↓DW due to CCC*	Reduction in disability weight (DW) during diagnosis and treatment phase due to a CCC reducing patient anxiety	Ferrante et al. [8]	0.67 (0.45 to 1.0)
		See Additional file 2	Log normal distribution

**Table 2:** Selected cost parameters used in the model (greater detail and full list provided in Additional files 5 and 4)

Variable name	Variable definition	Source, derivation and application	Expected value and 95 % uncertainty interval
*Incremental CCC cost from diagnosis to surgery*	Incremental cost of CCC programme from provisional diagnosis to surgery (difference in costs for pathway of care with CCC minus pathway of care in business-as-usual comparator)	Consultation with local health care professionals (costed based on average salaries + 50 % overheads)	$64.03 per patient
			($29.42 to $98.64)
			Normal distribution
		See Additional file 5	
*Incremental CCC cost from surgery to start of chemotherapy*	Incremental cost of CCC programme from surgery to start of chemotherapy	Consultation with local health care professionals (costed based on average salaries + 50 % overheads)	$5.00 per patient
			($-10.39 to $20.39)
			Normal distribution
		See Additional file 5	
*Cost of chemotherapy per patient*	Cost per patient of 12 cycles of chemotherapy with oxaliplatin over 6 months	Bottom-up costing approach including cost of pharmaceuticals, outpatient attendance and overheads.	$17,811.78 per patient ($14,494.69 to $21,390.41)
			Gamma distribution
		See Additional file 4	
*Dietician costs*	Additional costs from dietician referrals precipitated by a CCC	Expert estimates	$115.89 per patient ($81.38 to $141.16)
		Referrals estimated to increase by 50 %, 2 contacts per referral. See Additional files 5 and 4	Gamma distribution
*Social worker costs*	Additional costs from social worker referrals precipitated by a CCC	Referrals estimated to increase by 42 %, 6 contacts per referral.	$327.95 to $483.97
		See Additional files 5 and 4	Gamma distribution

#### Baseline incidence, survival and mortality data

The estimated incidence rates of colon cancer were calculated across all combinations of sex, age, ethnicity (Māori, non-Māori), and socio-economic status (three levels) from New Zealand Cancer Registry data. These were disaggregated by ethnicity and deprivation using linked census-cancer data (as described elsewhere) [22]. This was further restricted to those with stage III colon cancer, and disaggregated by receipt of surgery and chemotherapy [23].

Colon cancer mortality rates (by time since diagnosis and socio-demographics) were estimated using excess mortality rate modelling on cancer registry data linked to mortality data. These rates were then adjusted to be specific to stage III colon cancer and receipt of surgery and chemotherapy (see Additional file 2). Background population mortality rates were derived from socio-demographic life tables [24].

#### Baseline waiting times to surgery and chemotherapy, and baseline coverage of chemotherapy

Baseline waiting times to surgery and waiting times from surgery to chemotherapy (without a CCC) by socio-demographics were estimated using data from a previous New Zealand hospital notes review study of over 600 colon cancer patients diagnosed between 1996 and 2003 [20]. Baseline coverage of chemotherapy was calculated for those who were eligible and did not refuse treatment, by age and socio-demographic group (see Additional file 2).

#### Effect of a CCC

As mentioned earlier, we specified that the health gain from a CCC intervention would be via four effects: reducing time from provisional diagnosis to surgery, reducing time from surgery to chemotherapy, improving the coverage of chemotherapy (there was little room for improvement in coverage of surgery) and reducing patients’ anxiety during diagnosis and treatment (thus improving patients’ quality of life).

We undertook systematic literature searches to determine the impact a CCC might have on increasing chemotherapy coverage, reducing waiting times (to surgery and from surgery to chemotherapy), and improving quality of life for cancer patients (see Additional file 3). Evidence for most of these was sparse. The only available source for estimating the impact of a CCC on improving receipt of chemotherapy was a study by Goodwin et al., assessing a CCC-type intervention in older patients with breast cancer in the United States [25]. Similarly, in order to estimate the impact of a CCC on reducing time from diagnosis to surgery as well as from surgery to chemotherapy, we drew on a retrospective case series analysis by Haideri et al., [10] again assessing the effect of a CCC-type intervention in women with breast cancer. The impact of a CCC on anxiety reduction and thus quality of life was estimated based on a study by Ferrante et al., [8] looking at the effect of a patient navigator on reducing anxiety after an abnormal mammogram. The available evidence was complemented by consultation with health professionals from different cancer centres (medical oncologist, colorectal surgeon, oncology nurses) in order to source estimates and specify distributions.

The key parameters for effect of a CCC are showed in Table 1, with more detail provided in Additional file 2. We deliberately specified generous uncertainty for each of these input parameters given the lack of robust published evidence. For example, we estimated that a CCC would improve the coverage of chemotherapy by 33 % (but with the 95 % uncertainty interval ranging from 9 to 65 %), and reduce the time between surgery and chemotherapy by 20 % (ranging from 3 to 48 %). For practical purposes, more effort was invested in estimating those input parameters where the uncertainty contributed significantly to overall ICER uncertainty (see Additional file 2 and Figs. 3 and 4 for such ‘key’ parameters).

**Fig. 2:**
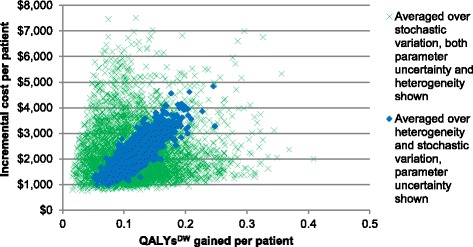
Main model analysis cost-effectiveness plane for CCC intervention compared to business-as-usual. Wider scatterplot showing results reflecting input parameter uncertainty and heterogeneity. Narrower scatterplot reflecting only input parameter uncertainty

**Fig. 3:**
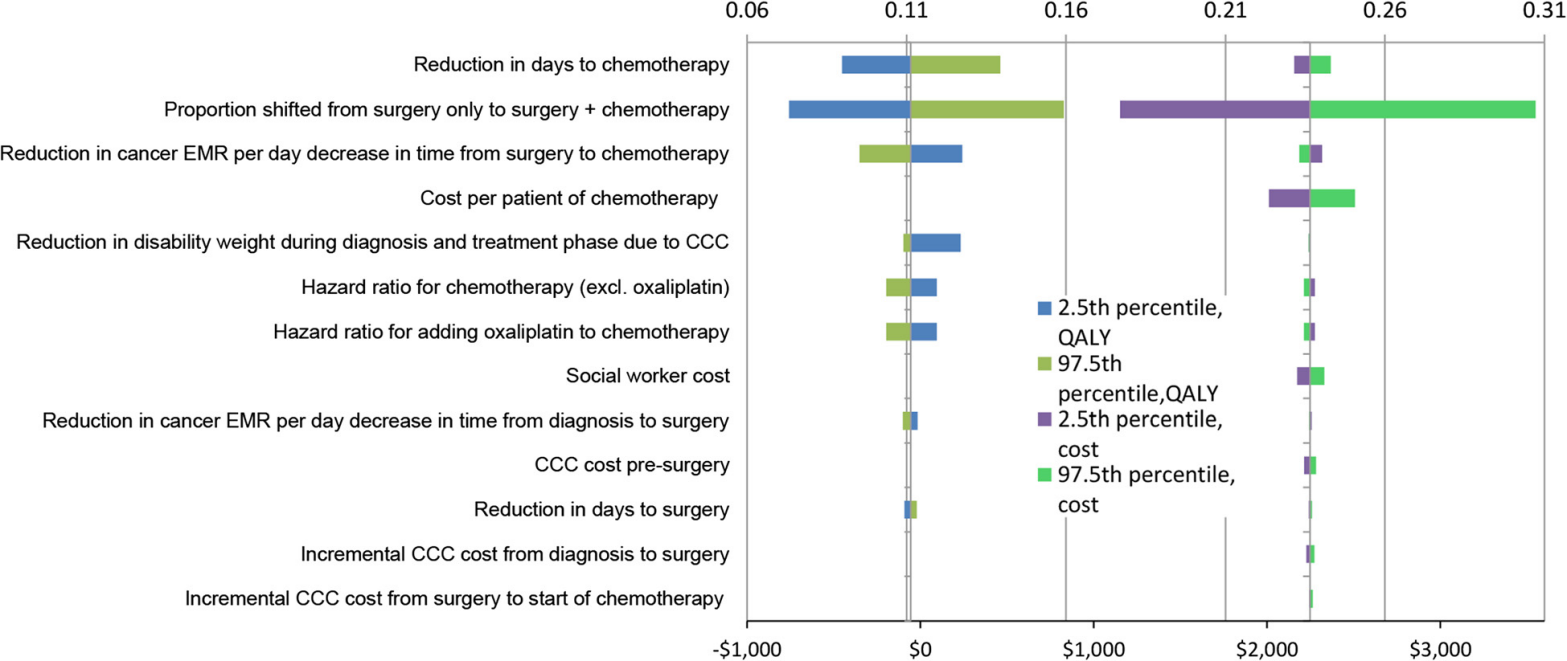
Tornado plot for QALYs gained (top axis) and cost (bottom axis) for 2.5^th^ and 97.5^th^ percentile values of input parameters. Above values are for single parameter values only. That is, the 2.5th (or 97.5th) percentile value of the parameter itself, and the mean expected value of all remaining parameters in the table, are modelled. There is no modelled parameter uncertainty. The estimates are averaged over heterogeneity and stochastic variation. EMR = excess mortality rate (due to cancer); CCC = cancer care coordinator

**Fig. 4:**
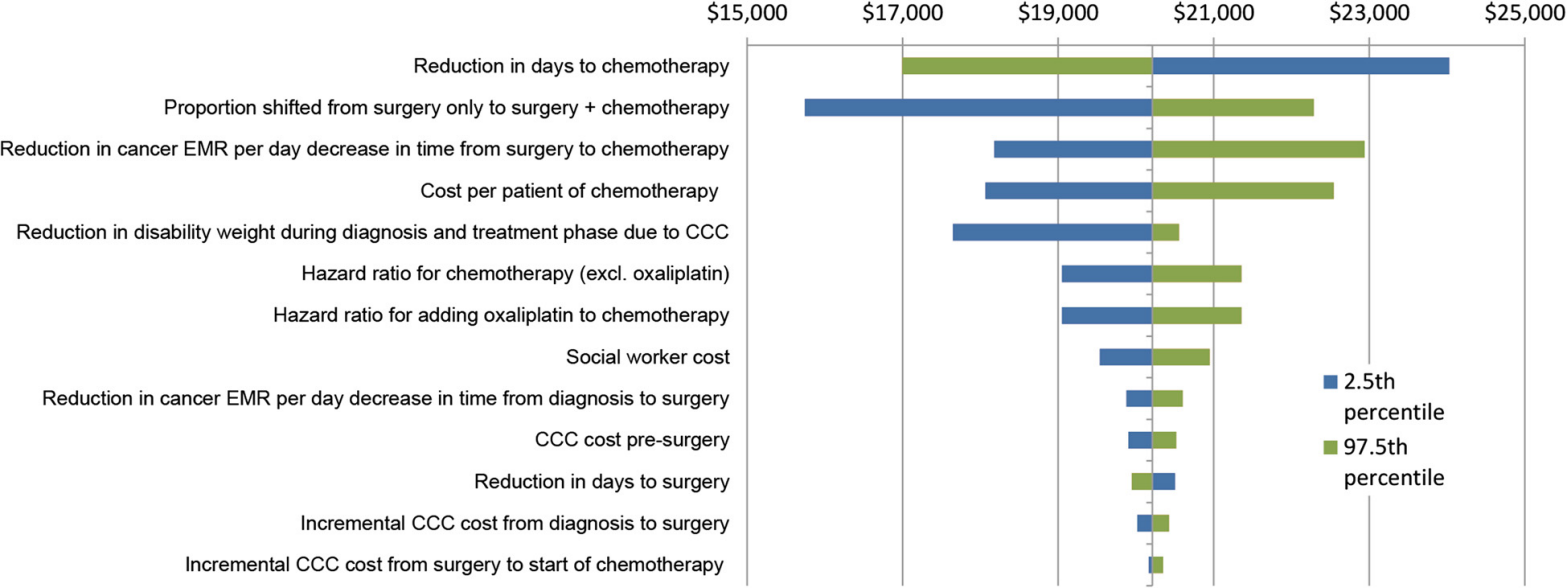
Tornado plot for ICER for 2.5^th^ and 97.5^th^ percentile values of input parameters. EMR = excess mortality rate (due to cancer); CCC = cancer care coordinator

#### Quality-adjusted life-years (QALYs) and disability weights

QALYs use many different health status valuation methods e.g. EuroQol (EQ5D) and the Health Utilities Index questionnaire. We used disability weights (DWs, where 0 is perfect health and 1.0 is equivalent to death) adapted from the Global Burden of Disease 2010 [26, 27]. Accordingly, we use the term QALY^DW^ in the Methods and Results section of this paper (but default to QALYs elsewhere).

Disability weights (DWs) were applied separately to four phases of colon cancer disease, by merging the recent Global Burden of Disease 2010 DWs (given for cancers overall) [27] with the relative difference in DW by cancer in the Australian BDS colorectal cancer model [26]. The phases were diagnosis and treatment, remission, pre-terminal and terminal, and the durations and attendant DWs are shown in Additional file 2. We also allow for sex- and age-specific background morbidity, using the expected level of background morbidity for all diseases combined from a recent New Zealand burden of disease study [28]. For example, for a 70–74 year old with an expected background morbidity equivalent to a DW of 0.3 and who was in the diagnosis and treatment (DT) phase of colon cancer (DW of DT phase = 0.288), their QALY^DW^ per annum was (1–0.3)X (1–0.288) = 0.498. QALYs^DW^ continued to be tallied in the model beyond the cure time (eight years post diagnosis) to death from other causes, or age 110 if still alive.

#### Health system costs

The health system costs were determined by strata of sex and age using a dataset of (nearly) all New Zealanders with their health system events (e.g. hospitalisation, lab test) all ascribed a cost. Following Van Baal et al. [29], we separately determined expected costs for those in the last six months of life. A critical health system cost for our model was that for chemotherapy, as many people moved from having surgery only to receiving chemotherapy post-operatively. We estimated the cost of chemotherapy at $NZ 17,812 per six month treatment per patient using a bottom-up costing approach including the cost of the pharmaceuticals, outpatient attendance and overheads (see Additional file 4). As with QALYs^DW^, health system costs continued to be tallied in the model beyond the cure time (eight years post diagnosis) to death from other causes or age 110 if still alive.

#### Incremental cost of CCC

The CCC intervention pathway was specified following review of the literature and consultation with local health care professionals, then costed (Additional files 1 and 5). The incremental cost of the CCC intervention is the cost of the care pathway with a CCC in place minus the cost of the care pathway in the business-as-usual comparator. To determine the time spent on coordinating activities where no CCC exists in the business-as-usual comparator, we calculated the cost per minute of coordinating activity being carried out based on the average salaries (plus 50 % overheads) of the personnel carrying out the activity. The incremental cost of the CCC programme itself is modest (e.g. expected values of $64.03 and $5.00 from provisional diagnosis to surgery and between surgery and chemotherapy, respectively). This is because whilst a CCC is introduced, other resources such as surgeon and nurse time previously spent on coordinating activities are released for use elsewhere. We also included the costs for increased allied health referrals precipitated by the CCC.

### Analyses

There is often considerable uncertainty in estimates of cost, health gain, and cost-effectiveness. This uncertainty comes from two sources: input parameter uncertainty (uncertainty about input parameters due to lack of perfect knowledge) and model structure uncertainty (uncertainty about the assumptions implicit in the model structure). Additionally, results also vary due to heterogeneity (differences by patient or population characteristics such as age, sex, ethnicity, and socio-economic status) [30]. We conducted a range of analyses to address input parameter uncertainty, heterogeneity, and model structure uncertainty. The types of analyses we conducted are described briefly below:

#### Main model analysis

Our main or full model analysis incorporates input parameter uncertainty and heterogeneity. As mentioned earlier, for input parameters where there is considerable uncertainty, a probability distribution around the best estimate is defined. Our main analysis captures input parameter uncertainty through a ‘looped’ or nested Monte Carlo simulation approach, involving millions of simulations in total. This approach is described in detail by Koerkamp et al. [30]. The effect of this input parameter uncertainty is then presented as 95 % uncertainty intervals (UIs) for QALYs^DW^, incremental costs, and ICERs. These main results’ can be presented for all stage III colon cancer patients combined, but also separately by heterogeneous patient types (e.g. different ethnic groups, young and old) – so called ‘heterogeneity analyses’.

#### Expected value analysis

This analysis uses only expected or central values for each input parameter; it does not allow for input parameter uncertainty. Such analyses are much quicker to run. We used this approach for a number of sensitivity and scenario analyses.

#### Ethnic equity analysis

The main analysis allows for variation in survival, morbidity and baseline times to event by ethnic group. While this approach uses the best available data, it also means that we value a life saved for Māori less than that for non-Māori. This is due to the fact that the higher expected background morbidity and lower life expectancy for Māori limits the QALYs^DW^ that can be gained. Therefore, we also undertook an ‘equity analysis’ where we applied the non-Māori least deprived background mortality rates and the non-Māori average background morbidity to Māori.

### Scenario analyses

We re-ran models for a range of scenarios to assess the impact of changing various model structure assumptions, such as the discount rate.

#### One-way sensitivity analyses

We also undertook a range of one-way sensitivity analyses and Tornado plots [31], using the 2.5^th^ and 97.5^th^ percentile values of input parameters, to assess which input parameters contributed the most to uncertainty in the model outputs (i.e. QALY^DW^, cost and ICER).

### Ethics

This analysis was conducted within the Burden of Disease Epidemiology, Equity, and Cost-Effectiveness (BODE^3^) research programme. The BODE^3^ programme is in compliance with the Helsinki Declaration. The Health and Disability Ethics Committee confirmed ethical approval was not required for BODE^3^ under section 11.8 and 11.9 of the New Zealand National Ethics Advisory Committee (NEAC) Guidelines for Observational Studies, as ethical approval is not required for secondary use of data for the purpose of quality assurance or outcome analysis when undertaken by those employed by the health service provider holding the information.

## Results

### Main model analysis

Figure 2 and Table 3 present the findings from our main model analysis. They depict uncertainty in incremental costs and QALYs^DW^ gained, due to input parameter uncertainty (model 1 in Table 3 and solid markers in Fig. 2). They additionally depict variation due to heterogeneity across sex, age, ethnicity and deprivation (model 2 in Table 3 and crosses in Fig. 2). We focus on the former here, and consider heterogeneity below.

**Table 3:** Main model analysis: incremental costs, QALYs^DW^ gained and ICERs for the CCC intervention compared to business-as-usual

	Incremental costs per patient (NZ$)	QALYs^DW^) gained per patient	ICER (NZ$ per QALY^DW^)
Model 1: Averaged over heterogeneity and stochastic variation; input parameter uncertainty only			
Mean	$ 2,271	0.121	$ 18,881
2.5 % percentile	$ 1,225	0.070	$ 13,442
Median	$ 2,226	0.119	$ 18,786
97.5 % percentile	$ 3,641	0.185	$ 24,610
Model 2: Averaged over stochastic variation; both heterogeneity and input parameter uncertainty included in distribution of outputs			
Mean	$ 2,239	0.120	$ 23,393
2.5 % percentile	$ 992	0.036	$ 6,290
Median	$ 1,972	0.113	$ 17,864
97.5 % percentile	$ 5,007	0.252	$ 72,041

Dollars are NZ$, for the year 2011. All costs and benefits discounted at 3 % per annum

The mean estimated QALYs^DW^ gain per patient was 0.121 (95 % UI 0.070 to 0.185), and the incremental cost $NZ 2,271 (95 % UI $1,225 to $3,641). Accordingly, the ICER was $NZ 18,881 per QALYs^DW^ gained (≈ $US 15,600, ≈ £UK 10,300) with relatively narrow uncertainty ($NZ 13,442 to $24,610) compared to the uncertainty in both QALYs^DW^ and cost due the strong correlation of increasing cost with increasing QALYs^DW^ (depicted graphically as the eclipse cloud in Fig. 2). This is because a key health benefit of CCC is increased coverage of chemotherapy, which is also a key cost driver.

### Heterogeneity analysis

There is a much larger scatter of points on the cost-effectiveness plane in Fig. 2 when variation by patient heterogeneity (sex, age, ethnicity and deprivation) in addition to input parameter uncertainty is depicted. Model outputs vary by these socio-demographics due to differences in inputs of time to event (surgery, chemotherapy, death from cancer and death from other causes) and expected population morbidity. Table 4 shows the expected value analysis outputs by socio-demographics. Variation by sex is modest. However, variation by age is large with decreased cost and (modestly) increased QALYs^DW^ gains among younger patients, leading to a more favourable ICER ($9,400). By socio-economic deprivation, the cost is greater for more deprived people, but the QALYs^DW^ gains less leading to a worse ICER ($22,800).

**Table 4:** Main model analysis by sex, age, ethnicity, and deprivation: incremental costs, QALYs^DW^ gained and ICERs for the CCC intervention compared to business-as-usual

Population	Incremental costs per patient (NZ$)	QALYs^DW^ gained per patient	ICER (NZ$ per QALY^DW^)
Total (expected value analysis)^a^	$2250	0.111	$20,200
*By sex*			
Males	$2050	0.118	$17,400
Females	$2520	0.121	$20,800
*By age*			
< 65 years	$1620	0.172	$9,400
≥ 65 years	$2490	0.106	$23,600
*By ethnicity by age*			
Māori	$3420	0.171	$20,000
< 65 years	$2810	0.223	$12,600
≥ 65 years	$3730	0.147	$25,300
Non-Māori	$2220	0.118	$18,800
< 65 years	$1510	0.167	$9,000
≥ 65 years	$2420	0.104	$23,300
*By deprivation*			
Least deprived tertile	$1880	0.125	$15,000
Most deprived tertile	$2620	0.115	$22,800
Equity analysis: using non-Māori least deprived mortality and non-Māori average background morbidity			
*By ethnicity by age (percentage variation from equivalent above standard analysis)*			
Māori	$3,780 (11 %)	0.251 (47 %)	$15,100 (−25 %)
< 65 years	$3,250 (16 %)	0.317 (42 %)	$10,300 (−18 %)
≥ 65 years	$4,070 (9 %)	0.222 (51 %)	$18,400 (−27 %)
Non-Māori	$2,240 (1 %)	0.121 (3 %)	$18,500 (−2 %)
< 65 years	$1,540 (2 %)	0.171 (2 %)	$9,000 (0 %)
≥ 65 years	$2,430 (0 %)	0.107 (3 %)	$22,800 (−2 %)

All models are expected value only; there is no parameter uncertainty

Dollars are NZ$, for the year 2011. All costs and benefits discounted at 3 % per annum. All values rounded to three meaningful decimal places

^a^Note that these results differ slightly from those in Table 3 due to not including uncertainty about parameters (due to long run time of models). Results in Table 3 are the preferred results, but the results in this table should be compared to this expected value analysis result which used the same modelling strategy

There are large differences in age structure by ethnicity in New Zealand, so we focus on the ethnicity by age comparisons. Within young and old, the incremental cost and QALYs^DW^ gained are both estimated to be larger for Māori and the ICER modestly higher for Māori (e.g. $25,300 for Māori ≥ 65 years compared to $23,300 for non-Māori ≥ 65 years).

### Scenario analyses

Table 5 shows how the findings vary for scenario analyses about model assumptions and structure. As mentioned earlier, for practical purposes the scenario analyses use expected values of the input parameters, not the full uncertainty about the input parameters.

**Table 5:** Scenario analyses: incremental costs, QALYs^DW^ gained and ICERs for the CCC intervention compared to business-as-usual (percentage difference to expected value analysis in parentheses)

Scenario	Incremental costs per patient (NZ$)	QALYs^DW^ gained per patient	ICER (NZ$ per QALY^DW^)
Expected value analysis^a^	$2,250	0.111	$20,200
*Varying phases of CCC intervention*			
a. CCC from diagnosis to surgery only	$80 (−96 %)	0.009 (−92 %)	$9,100 (−55 %)
b. CCC from surgery to chemotherapy only	$2,170 (−4 %)	0.104 (−6 %)	$20,900 (3 %)
*Variations to discount rate*			
c. 0 % per annum discount rate	$2,520 (12 %)	0.148 (33 %)	$17,100 (−15 %)
d. 6 % per annum discount rate	$2,080 (−8 %)	0.088 (−21 %)	$23,600 (17 %)
*Variation to epidemiological parameters*			
e. Set all DWs (incl pYLDs) to zero (= ‘life years’ gained)	$2,250 (0 %)	0.150 (35 %)	$15,000 (−26 %)
f. Exclude improved quality of life impact of CCC	$2,250 (0 %)	0.100 (−10 %)	$22,400 (11 %)
g. Exclude improved survival due to quicker to surgery	$2,240 (0 %)	0.107 (−4 %)	$21,000 (4 %)
h. Exclude improved survival due to quicker to chemotherapy	$2,130 (−5 %)	0.084 (−24 %)	$25,200 (25 %)
i. Exclude increasing % of patients getting chemotherapy	$800 (−64 %)	0.061 (−45 %)	$13,000 (−36 %)
j. Exclude oxaliplatin	$2,020 (−10 %)	0.095 (−14 %)	$21,300 (5 %)
*Variation to cost parameters*			
k. Scale all health system costs up 20 %	$2330 (4 %)	0.111 (0 %)	$20900 (3 %)
l. Scale all health system costs down 20 %	$2170 (−4 %)	0.111 (0 %)	$19500 (−3 %)
m. Exclude dietician and social worker intervention costs	$1730 (−23 %)	0.111 (0 %)	$15600 (−23 %)
n. Exclude unrelated health system costs (i.e. include costs up to cure time only)	$1780 (−21 %)	0.111 (0 %)	$16000 (−21 %)

All models are expected value only; there is no parameter uncertainty

Dollars are NZ$, for the year 2011. Unless stated otherwise, all costs and benefits discounted at 3 % per annum. All values rounded to three meaningful decimal places

*pYLDs* prevalent years of life lived with disability, which is used as the ‘expected’ amount of morbidity by sex, age and ethnicity

^a^Note that these results differ slightly from those in Table 3 due to not including uncertainty about parameters (due to long run time of models). Results in Table 3 are the preferred results, but the results in this table should be compared to this expected value analysis which used the same modelling strategy

The impact of the CCC intervention on quality of life (by reducing the anxiety of patients) and improved survival due to getting to surgery quicker were modest drivers of our analysis. Conversely, increased coverage of chemotherapy and getting to chemotherapy quicker were major drivers of model outputs. For example, excluding any effect on the number of patients getting chemotherapy reduced the incremental cost by 64 %.

The majority of health gain and cost from a CCC occurred in the post-surgical phase, due to increased coverage of chemotherapy and decreased time to chemotherapy. The ICER was less ($9,100) for the provisional diagnosis to surgery component of the intervention (less health gain and also less cost). Varying the discount rate had a moderate impact in an expected manner.

If we excluded the expected population morbidity, the QALYs^DW^ gained increased due to no assumed loss of quality of life in survivors, and therefore the ICER reduced by 27 %. A life-years gained analysis resulted in a similar ICER.

Scaling up and down the health system costs (excluding chemotherapy) had a modest impact only, but excluding the assumed increased referrals to dieticians and social workers reduced costs by 23 %. Finally, our most comprehensive model included unrelated health system costs into the future, and if these are excluded the (discounted at 3 %) costs decrease by 21 %.

### Ethnic equity analysis

Applying non-Māori least deprived mortality and non-Māori average background morbidity to both Māori and non-Māori resulted in an increase in the QALYs^DW^ gained by 47 % for Māori and a reduction in the ICER by 25 % such that it becomes lower for Māori compared to non-Māori ($15,100 compared to $18,500; Table 4).

### One-way sensitivity analyses

Uncertainty in the following four input parameters has the biggest impact on uncertainty in model outputs (Figs. 3 and 4): the proportionate reduction in days to chemotherapy (QALYs^DW^ and ICER), increased coverage of chemotherapy (QALYs^DW^, cost and ICER), improved survival from getting chemotherapy quicker (QALYs^DW^ and ICER), and cost per patient of chemotherapy (cost and ICER).

## Discussion

We find that CCCs, for colon cancer stage III at least, are cost-effective for a willingness to pay of NZ$20,000 (about US$16,500; using mean value) or NZ$25,000 (about US$ 21,000; using the upper uncertainty limit). The major drivers of health gain come from increased coverage of effective treatments and reduced time to effective treatments via better coordination of care, a conclusion that should be generalisable to other CCC programmes. Impacts through changes in quality of life, if just during the delivery of the programme itself, are unlikely to be a major driver of health gains. We found substantial heterogeneity, in that health gains were greater and costs less for younger patients resulting in better cost-effectiveness. However, this cost variation by age may not be generalisable to other CCC interventions, as in our evaluation young people in the ‘business-as-usual’ comparator were already almost all receiving chemotherapy – the key cost driver. Social inequalities in health are a major policy concern worldwide [32]. There are large health inequalities between Māori and non-Māori in New Zealand [33], including for colon cancer survival [20]. Our evaluation suggests that CCC would achieve greater health gains for Māori patients due to lower receipt of chemotherapy and longer wait times in the business-as-usual arm. However, this finding is contingent on our assumption that the proportionate increase in chemotherapy coverage, and proportionate reduction in time to chemotherapy from CCC, is constant across ethnic groups. When we conduct an equity analysis with background mortality and morbidity rates held constant by ethnic group, we find greater ‘equity weighted’ health gains for Māori and a comparable ICER between Māori and non-Māori. Incorporating equity in cost-effectiveness evaluations is an under-developed area. Previous researchers have also attempted incorporating equity [34]; our approach should be taken as one possible method to consider and subject to further scrutiny.

Health economic decision models are conducted to assist prioritisation of resources. Questions that decision-makers want answered often require collating data that is uncertain, and making justified assumptions. Whilst we have high quality New Zealand data for many of the baseline parameters in our model (e.g. survival by socio-demographics), our evaluation still has many uncertain input parameters (e.g. the effect of a CCC). Despite that, we believe that we have demonstrated that with careful parameter specification and most particularly inclusion of (appropriately) wide uncertainty can still lead to useful conclusions. Through our scenario (Table 5) and sensitivity analyses (Figs. 3 and 4), we think we have provided enough alternative analyses for interested readers who disagree with our parameterisation and assumptions to find something that accords with their ‘prior’. Regarding our own best estimates and’prior’, we conclude that CCC for stage III colon cancer at least is probably cost-effective and pro-equity.

We are aware of three cost-effectiveness analyses (CEAs; two for breast cancer and one for any terminal cancer patient) [15–17] and one cost-benefit analysis [35] of CCC-type interventions. It is difficult to compare our results with these previous evaluations due to the different cancers, variation in where the CCC intervention was provided in the cancer care pathway, and different approaches in the economic analyses. In short, one study found the CCC-type intervention was no different in cost to usual care and did improve quality of life indicators [17], another found no improvement in quality of life outcomes but a reduction in costs with the intervention [15], and a third showed an incremental cost per life year gained of $US 95,625 (from abnormal screening to diagnostic follow-up for breast cancer patients). Unlike our model, one of the CEAs assessed the effect of patient navigators in a specific population (low-income, ethnic minority and 40 years or older) [15]; a strength of our evaluation is the explicit incorporation of heterogeneity by sex, age, ethnicity and deprivation permitting evaluations by type of patient (Table 4). Other relative strengths of our evaluation include the systematic reviews for each key input parameter (although the studies found were often poor quality), input obtained from experts where there was a lack of published data, clear explanations for assumptions where they were made (Additional file 2), inclusion of both morbidity and mortality, and probabilistic sensitivity analysis. In contrast, two of the CEAs [15, 17] were conducted alongside randomised controlled trials with access to raw data for quality of life measures and cost data; we relied on data collected from a resource use survey for our cost data.

We cannot directly generalise our evaluation for stage III colon cancer patients to other settings, but we may be able to extrapolate in terms of principle. For example, we would expect health gains to be less if there is already good coordination in place through means other than CCC such as well functioning multi-disciplinary meetings and good IT systems for tracking patient care. However, costs would also be less, meaning the ICER may not be too different. Second, our evaluation demonstrates that increasing receipt of effective treatments and reducing time to effective treatments is the major benefit of a CCC programme, not the quality of life impacts per se.

Whilst decision-makers need information now, and our study responds to that imperative, there is considerable uncertainty in the input parameters (as shown in Figs. 2, 3 and 4). Regarding stage III colon cancer per se, it is the uncertainty in these three intervention effects that matters most: how much the CCC reduces time to chemotherapy, what proportion of those eligible for chemotherapy but not receiving it pre-CCC receive it post-CCC; and the improvement in survival from getting chemotherapy quicker. Research, such as randomised trials of CCC, to estimate these parameters with greater accuracy would increase the accuracy of modelling such as our study. Second, undertaking similar evaluations for different phases of the cancer patient journey (e.g. support during remission) and different cancers (e.g. lung cancer where treatment efficacy is less, or breast cancer where survival is better and reducing time to treatment is perhaps not so critical) is necessary to have a greater understanding of where CCC should be prioritised within the full range of cancer services.

## Conclusions

For stage III colon cancer, we estimate that CCCs do improve health outcomes, and is a cost-effective intervention for younger patients at least. It also appears – in the New Zealand context at least – to afford as much if not more benefit to Māori patients who usually have worse outcomes, with approximately the same cost-effectiveness as the general population. Thus, the intervention is probably pro-equity. Generalising to CCC for other phases of the cancer patient journey, and other cancers, is difficult due to differing baseline patient journeys, survival rates and treatment efficacy. However, our study suggests that CCCs that increase receipt of, and minimise time to, effective treatments should be prioritised.

## Additional files

Additional file 1:
**Provides additional details on definition of intervention and comparator.** (PDF 419 kb) Additional file 2:
**Provides additional details on the model and input parameters within the model.** (PDF 1334 kb) Additional file 3:
**Documents the literature search strategies used.** (PDF 764 kb) Additional file 4:
**Provides additional details on baseline health system costs by disease state.** (PDF 827 kb) Additional file 5:
**Contains the event pathway and details of costing the baseline and intervention.** (PDF 752 kb)

## Abbreviations

BDS: Burden of Disease Study
BODE^3^: Burden of Disease Epidemiology, Equity and Cost-Effectiveness Programme
CEA: Cost-effectiveness analyses
CCC: Cancer care coordinators
CNS: Clinical nurse specialist
DES: Discrete event simulation
DT: Diagnosis and treatment
DWs: Disability weights
EMR: Excess mortality rate
HR: Hazard ratio
ICER: Incremental cost-effectiveness ratio
NICE: National Institute for Health and Care Excellence
UIs: Uncertainty intervals
QALY: Quality-adjusted life-year
QALY^DW^: Quality-adjusted life-year utilising disability weights for health status valuation
