# Supplementary material for: Cancer care coordinators in stage III colon cancer: a cost-utility analysis
Source: BMC Health Serv Res. 2015 Aug 5;15:306. doi: 10.1186/s12913-015-0970-5 (PMC4523949; doi:10.1186/s12913-015-0970-5)
Supplement: Additional file 1: — Provides additional details on definition of intervention and comparator. (PDF 419 kb) [file 12913_2015_970_MOESM1_ESM.pdf]

Additional File 1: Intervention and comparator definition

For ‘Cancer care coordinators in Stage III colon cancer: a cost-utility analysis’

Tony Blakely[1‡], Lucie Collinson[1], Giorgi Kvizhinadze[1], Nisha Nair[1], Rachel Foster[1], Elizabeth Dennett[2], Diana Sarfati[1]

[1]Burden of Disease Epidemiology, Equity, and Cost-Effectiveness (BODE[3]) Programme, Department of Public Health, University of Otago Wellington, PO Box 7343, Wellington, New Zealand

[2]Department of Surgery, University of Otago Wellington, PO Box 7343, Wellington, New Zealand

[‡]corresponding author

Contents

ADDITIONAL FILE 1: INTERVENTION AND COMPARATOR DEFINED 2

Specification of the CCC intervention 3

Specification of the comparator coordination services 5

Differences in non-coordination services between intervention and comparator 6

Additional File 1: Intervention and comparator defined

It is useful to conceptualise three scenarios (Figure 1): intervention – cancer care coordinator (CCC) programme; comparator – approximation of current ad hoc business-as-usual provision of coordination services; and a hypothetical partial null scenario where no coordination services are provided. The substantive comparison of interest is between the intervention and the comparator. Ideally, costs and effect sizes are estimated for this comparison. Using costs as an example, it may often be more sensible to estimate the full costs of the intervention compared to the partial null, the full costs of the comparator compared to the partial null, and then take as the direct cost of the programme the difference between the two. Regardless of the approach, a careful specification of exactly what is included in the care coordinator programme and its comparator is required – and is the objective of this Appendix.

Figure 1: Intervention, comparator, and partial null

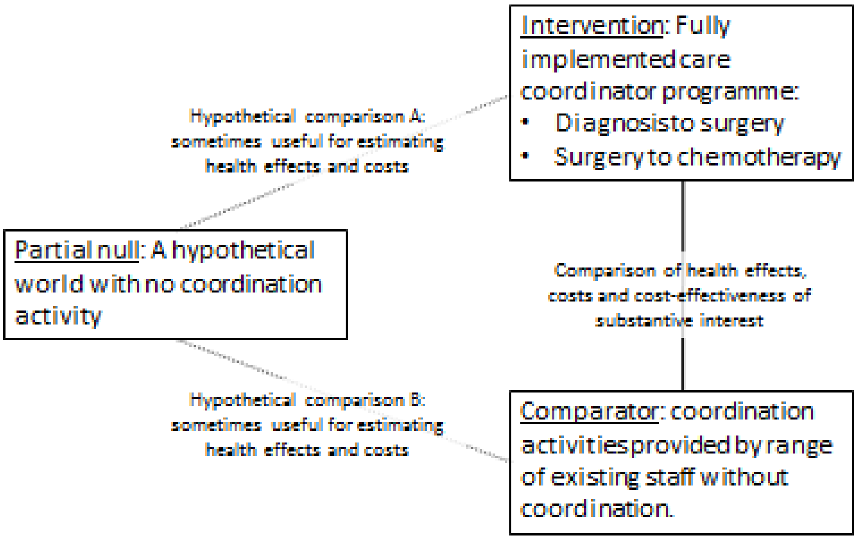

Our specification of the comparator approximates the practice of some hospital boards in New Zealand at 2012, but it is not necessarily the average level of ad hoc coordination. Further, we have 'tuned' and simplified some of the specifications of the comparator so as to align with previous research that provides estimates of effect size (e.g. the increase in uptake of chemotherapy with CCC versus the comparator).

Specification of the CCC intervention

The CCC intervention we are modelling begins at the point of provisional diagnosis of colon cancer (from imaging or more commonly colonoscopy) and continues until initiation of chemotherapy for patients with confirmed stage III colon cancer. In order to model the same pathway of care emergency presentations are excluded.

The CCC is a clinical nurse specialist (CNS) with clinical experience in colorectal surgery. The CCC requires the expert knowledge, skillset and experience of a CNS in order to be able to firstly conduct a comprehensive nursing assessment and provide necessary care, secondly to provide the patient with sufficient information with regards to their health and treatment plan and answer their questions and thirdly to be able to detect symptoms which may require referral to other specialties or senior surgical involvement and if not addressed may have a negative effect on the patient's outcome and /or affect the timeliness of care.

They are hospital-based and coordinate care for patients with a provisional (and later histologically confirmed) diagnosis of colon cancer stage III. They make initial contact with patients at the time of provisional diagnosis (which could be in the surgical outpatient clinic, gastroenterology outpatient clinic, colonoscopy suite, short-stay unit, surgical or medical ward or other setting).

They provide additional informational and emotional support after the consultation with the surgeon and coordinate arrangements for pre-operative assessments such as blood tests, imaging

and clinical examination and try to reduce the number of individual trips to the hospital by the patient (and carer) for each of these.

At the point of provisional diagnosis (or another preferred time for the patient in hospital) the CCC assesses the patient for barriers of access to care such as transport, financial or social. Where necessary they will be in communication with other specialties or their general practitioner to obtain information with regards to the patient’s comorbidities, their severity and on-going management.

The CCC will then liaise with the patient with regards to attending pre-operative assessment appointments and investigations and attempt to reduce any potential barriers to accessing care or those that may cause delay. Once pre-operative assessments and investigations are complete and results are available the CCC will communicate with the anaesthetic team and surgical team to ensure they are still planning to go ahead with surgery (decisions regarding patients listed for surgery are usually discussed at multi-disciplinary meetings (if they exist) which the CCC would attend). Assuming the patient, the surgical team and the anaesthetic team agree to go ahead with the surgery, the CCC will act as a point of contact for both teams, the patient and their GP to ensure timely provision of surgery.

Post-operatively the CCC will liaise with the ward staff to estimate the patient’s discharge date (depending on post-op recovery), repeat a social needs assessment and coordinate referrals to allied health or other medical specialties to ensure timely and safe discharge. Where necessary the CCC will ensure timely and adequate referrals are made to the GP and other specialities with regards to optimising the care of the patients’ comorbidities post-operatively.

Once the patient is discharged from hospital the CCC will continue to act as a point of contact for the patient (and healthcare team) and provide support during this period of high anxiety as the patient waits for the lymph node biopsy results (taken during surgery). The CCC tracks the histology report and ensures the follow-up appointment is booked with the surgical team for the patient to be given

the results. The CCC also ensures the possibility of adjuvant chemotherapy is discussed by the surgical team (ideally at the multi-disciplinary team meeting if this exists) and the referral is received by the oncology team. The CCC will ensure timely access to any post-operative investigations such as blood tests so as not to delay the initiation of chemotherapy treatment.

The CCC will act as a patient advocate and liaise with the GP and community health care services to optimise any additional post-operative pain relief, bowel function care and physical mobility (such as pharmacists, physiotherapists and occupational therapists). The CCC will have the clinical experience to pick up on any concerning symptoms which may extend the post-operative recovery period and communicate these concerns with the relevant health professional and coordinate referrals as required.

During the six month course of adjuvant chemotherapy the community cancer nurses take over from the CCC as the point of contact for patients and provide the informational and emotional support required during this period and coordinate any necessary investigations and referrals. We are therefore not modelling the CCC intervention during the six month period patients are receiving chemotherapy. Other studies have shown CCC-type programmes as being able to reduce treatment interruptions but on discussion with local clinical experts we chose not to model this as few patients experience treatment interruptions during adjuvant chemotherapy in colon cancer and those that occur are unlikely to be able to be prevented by a CCC.

Specification of the comparator coordination services

Coordination activity occurs in the absence of a specific CCC programme. Table 1 describes who we assume to be providing in our comparator – which roughly equates to an average hospital board in New Zealand. We assume that the CCC programme will displace the number of different health professionals currently carrying out these activities, meaning that there are cost savings to be deducted from cost increases from implementing a CCC programme.

Differences in non-coordination services between intervention and comparator

The supplanting of the comparator with a CCC programme will also alter non-coordination health sector activity. An obvious example is the increased coverage of chemotherapy, meaning more people are exposed to the costs and benefits of chemotherapy. These impacts are directly captured in the model. Second, CCC services will increase the use of allied health services such as dietician and social services. As these activities are not captured formally in the baseline costs of the economic decision model, they are included in the calculation of incremental direct costs as described and quantified in Additional File 4.

Table 1: Intervention versus comparator specification of coordination services

	**CANCER CARE COORDINATOR INTERVENTION**<br>Task provided by cancer care coordinator (CCC)	**COMPARATOR**<br>Same task provided ad hoc by one or more of the following healthcare workers
**From provisional diagnosis to surgery**		
1	Explains the pre-operative assessment procedures to the patient at the time of provisional diagnosis of colon cancer	Surgical team (consultant, registrar, registered nurse) and/or stoma therapy nurse (clinical nurse specialist or registered nurse) if it is thought a stoma will be needed
**2**	Conducting a social needs assessment (e.g. support at home, transport access)	Surgical team (consultant, registrar, registered nurse) and/or stoma therapy nurse (clinical nurse specialist or registered nurse) if it is thought a stoma will be needed
**3**	Making and tracking necessary support referrals for pre-operative assessment and admission (e.g. for transport support, social care, allied health etc.)	Nursing staff (registered nurses, clinical nurse specialists, stoma therapy nurses, patient flow coordinator) administration staff, general practitioners and district nurses
**4**	Coordinating and tracking pre-operative medical investigations and assessments required prior to pre-operative physician assessment (ensuring timeliness and correct sequence of these)	Surgical team and pre-assessment team (consultant, registrar, house surgeon, clinical nurse specialist, registered nurse), administration staff
**5**	Acting on delays associated with pre-operative investigations and assessments (e.g. including both transport and social support delays as well as timeliness of investigations)	Nursing staff, (registered nurses, clinical nurse specialists, stoma therapy nurses, patient flow coordinator) administration staff, general practitioners and district nurses and house surgeon
**6**	Following the pre-operative assessment, coordinating and tracking any further necessary investigations and assessments required before surgery	Surgical team and pre-assessment team (consultant, registrar, house surgeon, clinical nurse specialist, registered nurse), administration staff
**7**	Coordinating, tracking and where possible acting on delays for other necessary support	Surgical team and pre-assessment team (consultant, registrar, house surgeon, clinical

	services required for accessing surgery (e.g. for transport support, social care, allied health etc.)	nurse specialist, registered nurse), administration staff
**From surgery to chemotherapy**		
**8**	Post-operatively, repeat social needs assessment and coordinate referrals to necessary allied health and support services to enable timely and successful discharge	Surgical team and ward staff (consultant, registrar, house surgeon, clinical nurse specialist, registered nurse), administration staff
**9**	Co-ordinating oncology referral at multidisciplinary team meeting post-surgery and ensuring patient is discussed	Surgical consultant or registrar
**10**	Tracking referral to oncologist for chemotherapy and ensuring appointment made	Administration staff, nursing staff (although no one individual exclusively responsible thus not always carried out)
**11**	Ensuring post-surgery follow-up appointment with surgeon booked	Administration staff, nursing staff, surgical team (although not always carried out)

94
