# Supplementary material for: Cancer care coordinators in stage III colon cancer: a cost-utility analysis
Source: BMC Health Serv Res. 2015 Aug 5;15:306. doi: 10.1186/s12913-015-0970-5 (PMC4523949; doi:10.1186/s12913-015-0970-5)
Supplement: Additional file 2: — Provides additional details on the model and input parameters within the model. (PDF 1334 kb) [file 12913_2015_970_MOESM2_ESM.pdf]

Additional File 2:
Economic decision model
For 'Cancer care coordinators in Stage III colon cancer: a cost-utility analysis'

Tony Blakely[1‡], Lucie Collinson[1], Giorgi Kvizhinadze[1], Nisha Nair[1], Rachel Foster[1], Elizabeth Dennett[2], Diana Sarfati[1]

[1]Burden of Disease Epidemiology, Equity, and Cost-Effectiveness (BODE[3]) Programme, Department of Public Health, University of Otago Wellington, PO Box 7343, Wellington, New Zealand

[2]Department of Surgery, University of Otago Wellington, PO Box 7343, Wellington, New Zealand

[‡]corresponding author

Contents

ADDITIONAL FILE 2: ECONOMIC DECISION MODEL 3

Overview of model 3

Table of all input parameters used in model 7

Heterogeneity in the baseline model 18

Stage III colon cancer rates by demographic strata 18

Stage III colon cancer rates and counts by socio-demographic and baseline clinical strata 21

Clinical transition time: diagnosis to surgery; surgery to chemotherapy 23

Excess mortality rates by socio-demographic (i) and clinical (j) strata 27

Morbidity 31

Intervention parameters 32

The proportion of people receiving surgery only in the comparator who additionally receive chemotherapy with the intervention 32

Excess mortality rates under intervention: increasing coverage of chemotherapy 36

Proportionate reduction in average days to surgery; Δ[Dx→$Surg_i$] ... 37

Proportionate reduction in average days to chemotherapy; Δ[Surg→$Chemo_i$] ... 39

Reduction in cancer excess mortality per day decrease in time to surgery; EMRR [Dx→Surgery/day] ... 40

Reduction in cancer excess mortality per day decrease in time to chemo; EMRR [Dx→ Chemo/day] ... 43

Proportionate reduction in disability weight ... 44

REFERENCES ... 47

Additional File 2: Economic decision model

This additional file describes in detail the simulation model used in analyses. The description is in four parts:

1. Overview of model
2. Table of all input parameters used in the model (detailed description of complex input parameters covered in parts 3, 4, and 5)
3. Heterogeneity in the baseline model:
    a. generation of colon cancer incidence in 2011 by demographic strata
    b. further stratification of incidence by baseline clinical strata treatment
    c. clinical transition times (e.g. provisional diagnosis to surgery) by socio-demographic and clinical strata.
    d. cancer (excess) mortality rates by socio-demographic and clinical strata
4. Morbidity
5. Intervention parameters

Regarding the input parameters, they come from BODE[3] data (e.g. projected population mortality rates), external data and estimates. All are summarised below (the input parameter tables in the main paper are abbreviated versions of Table 1 in this file). All sources and assumptions are detailed in this file, although the more detailed literature reviews and methods (e.g. costing and input parameter estimation) are in Additional Files 3-5.

Overview of model

Much of the morbidity and mortality impact of the CCC intervention will be through increased timeliness of care – that is shortening transitions through the event pathway. Thus, time to event modelling is preferable. We also place emphasis on modelling population heterogeneity. Thus, we elected to use discrete event simulation (DES) modelling.

Regarding baseline population heterogeneity, we generated estimates from New Zealand data of the following by strata of sex, age, ethnicity and deprivation:

- incidence rates and counts of stage III colon cancer in 2011, which become probability distributions for sampling 'types of people' to model.
- cancer excess mortality rates (EMRs; i.e. death rates among cancer patients beyond that expected given sub-population life tables [1]) for cases diagnosed in 2011, and by time from diagnosis, for the above socio-demographic and baseline clinical strata (i.e. surgery only or surgery and chemotherapy, j=1 and j=2). These rates (and those derived from them based on intervention parameters below) are then converted to cumulative distribution functions (CDF) for later DES modelling.
- population mortality rates from life tables that are also then converted to CDFs.
- several clinical transition times: days from provisional diagnosis to surgery; days from surgery to commencement of chemotherapy. These were again specified as CDFs, given the cumulative proportion of people (of a certain socio-demographic group) that will have transitioned to the next phase by any number of days.

The sources and parameterisation of this baseline heterogeneity is described in more detail in the remainder of this file.

Regarding parameterisation of the intervention effect itself, there are both epidemiological and intervention (incremental) cost parameters. The intervention parameters (all assumed constant across population heterogeneity) are of four types:

- Proportionate reductions in transition times through the treatment pathway. For example, if CCC reduces time to surgery by 20%, then this intervention parameter is applied to all the individual sampled transit times in the DES microsimulation.

- Proportionate reductions in cancer excess mortality rates (EMR), usually due to hastening of transit. For example, there may be 0.5% reduction in the EMR for each day earlier that chemotherapy is commenced. Thus, the actual change in the EMR in a given simulation is a function of both the parameter introduced in this bullet point, and that in the previous bullet point.
- Increased coverage of chemotherapy. For example, the proportion of people receiving surgery only in the baseline who then move to receiving surgery and chemotherapy with the CCC programme.
- And a percentage reduction in the diagnosis and treatment morbidity weight or DW due to improved quality of life with CCC.

Note that whilst all these epidemiological intervention parameters are assumed constant across population heterogeneity, the *absolute* impact (e.g. number of days gained) will vary by socio-demographics given differing baseline parameters. For example, Māori have higher EMR meaning that a constant proportionate reduction in the EMR will incur a greater absolute benefit to Māori. The sources and derivation of these epidemiological parameters is described in greater detail below.

Regarding the economic decision model itself, this involved the following general schema. First, comparator and intervention arms were modelled in parallel. Second, the population heterogeneity, parameter uncertainty and stochastic variability were incorporate [2]. Accordingly, we followed a general schema of selecting the broad population groups to model, randomly sampling from these types of people (i.e. socio-demographic strata i) and from the probability distributions of the intervention parameters as the outer loop in the Monte Carlo simulations. In the next loop, the inner loop, we sampled individuals per clinical strata (surgery vs. surgery and chemotherapy) and their random walks (stochastic variation) were simulated accordingly.

Figure 1: Discrete event simulation (DES) model structure reflecting heterogeneity, parameter uncertainty and stochastic variation of individual level parameters

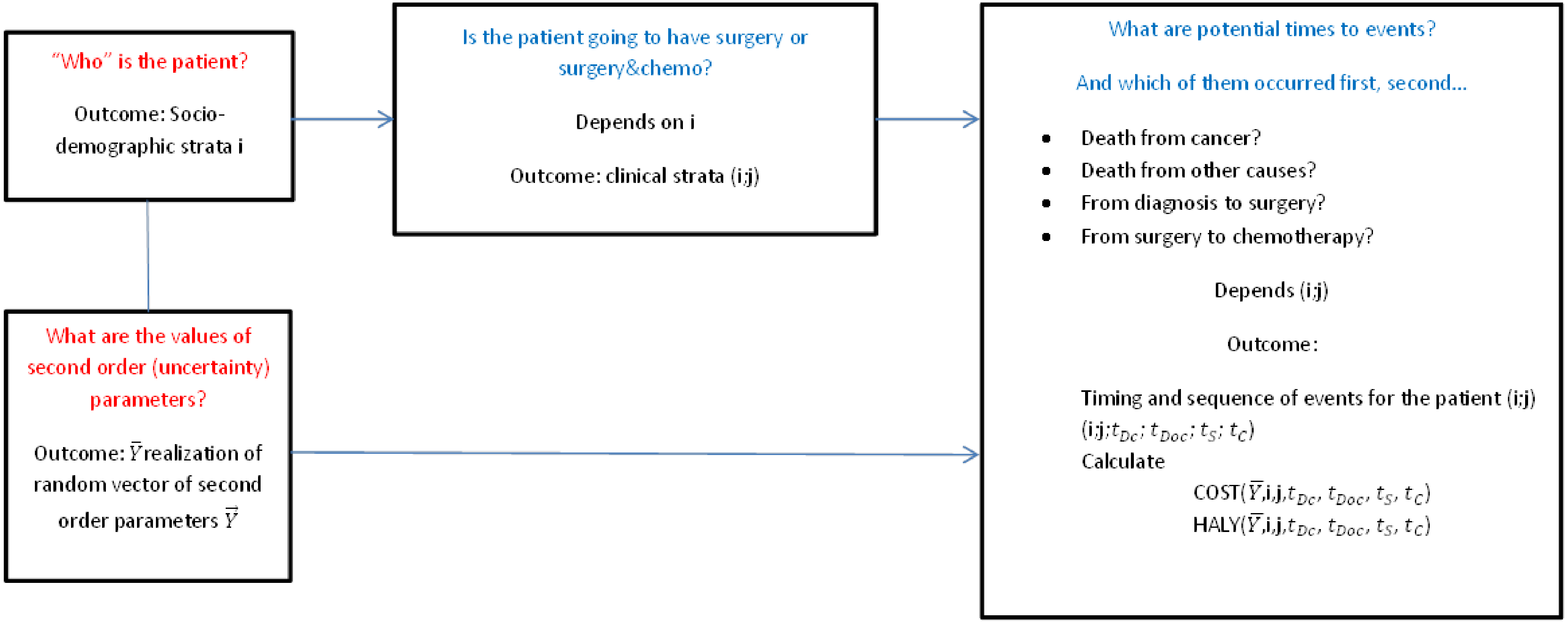

Table of all input parameters used in model

The full list of input parameters used in the model is presented here at tabular form. The text that follows explains in greater detail how complex input parameters were derived and applied to the model.

Table 1: Full Input Parameter Table

VARIABLE NAME	VARIABLE DEFINITION	SOURCE	DERIVATION FROM SOURCE & APPLICATIO N TO MODEL	HETEROGENEITY BY AGE/SEX/ ETHNICITY/ DEPRIVATION	EXPECTED VALUE	UNCERTAINTY INTERVAL (95% UI)	DISTRIBUTION	SENSITIVITY of ICER to this variable[1]
Time to 'Event'								
A. Time to death from colon cancer	Time from provisional diagnosis of colon cancer to death from colon cancer (using colon cancer excess mortality rates for stage III colon cancer by time since diagnosis)	NZ Cancer Registry data linked to mortality data[3]	See text below	Yes (by age, sex, ethnicity, deprivation). Further disaggregated by receipt of surgery alone or surgery plus chemotherapy.	See Figure 5 and Figure 6 below	Nil	Nil	n/a

[1] The extent that uncertainty in an intervention input parameter contributes to overall uncertainty in the ICER (i.e. variation in ICER for the 2.5^{th} to 97.5^{th} percentile values of this input parameter, as a percentage of the 95% UI from the full Monte Carlo analysis). For low percentage values, further improvement in estimation is not warranted. For high percentage values, the input parameter must be examined closely, e.g. in future research, by decision-makers in weighing the assumptions of the model, etc. This column is also presented visually as the tornado plot analysis in the main article.

VARIABLE NAME	VARIABLE DEFINITION	SOURCE	DERIVATION FROM SOURCE & APPLICATIO N TO MODEL	HETEROGENEITY BY AGE/SEX/ ETHNICITY/ DEPRIVATION	EXPECTED VALUE	UNCERTAINTY INTERVAL (95% UI)	DISTRIBUTION	SENSITIVITY of ICER to this variable[1]
B. *Time to death from other causes*	Time from diagnosis of colon cancer to death from other causes (using background or expected population mortality rates).	Projected NZ Life Tables Report[4]	See text below	Yes (by age, sex, ethnicity, deprivation).	Projected NZ Life Tables Report[4]	Nil	Nil	n/a
C. *Time from diagnosi s to surgery in days*	Usual time from provisional diagnosis of colon cancer to surgery without a CCC	Hospital notes review of 600 colon cancer patients 1996-2003[5]	See text below	Yes (by age, sex, ethnicity, deprivation).	22.6% of patients assumed to have zero time to surgery (diagnosed at surgery or emergency presentation).<br><br>For rest: mean time ranged from 13 days for young non-Māori female to 40 days for old Māori male. See Table 6 below.	Nil<br><br>See Figure 4 below.	Nil<br><br>Gamma	n/a<br><br>n/a

VARIABLE NAME	VARIABLE DEFINITION	SOURCE	DERIVATION FROM SOURCE & APPLICATIO N TO MODEL	HETEROGENEITY BY AGE/SEX/ ETHNICITY/ DEPRIVATION	EXPECTED VALUE	UNCERTAINTY INTERVAL (95% UI)	DISTRIBUTION	SENSITIVITY of ICER to this variable[1]
D. *Time from surgery to start of chemoth erapy*	Usual time from surgery to start of chemotherapy without a CCC	Hospital notes review of 600 colon cancer patients 1996-2003[5]	See text below	Yes (by age, sex, ethnicity, deprivation).	Mean time ranged from 51 days for young non-Māori female to 97 days for old Māori male. See Table 6 below.	See Figure 4 below.	Gamma	n/a
E. *Time from beginnin g to end of chemoth erapy*	Usual duration of chemotherapy in stage III colon cancer	Best practice guidance (Des Guetz meta-analysis[6] and Medsafe	Nil	No	6 months	Nil	Nil	n/a
Durations of Cancer Phases								
Duration of PT phase	Duration of pre-terminal (PT) phase	Relies on variable A defined above.	If dying from colon cancer, assumed one month in terminal state and 3 months before in pre-terminal state.	No	4 to 1 months prior to event A (death from colon cancer)	Nil	n/a	n/a

VARIABLE NAME	VARIABLE DEFINITION	SOURCE	DERIVATION FROM SOURCE & APPLICATIO N TO MODEL	HETEROGENEITY BY AGE/SEX/ ETHNICITY/ DEPRIVATION	EXPECTED VALUE	UNCERTAINTY INTERVAL (95% UI)	DISTRIBUTION	SENSITIVITY of ICER to this variable[1]
Duration of T phase	Duration of terminal (T) phase	Relies on variable A defined above.	If dying from colon cancer, assumed one month in terminal state and 3 months before in pre-terminal state	No	1 month prior to event A (death from colon cancer)	Nil	n/a	n/a
Duration of DT phase	Duration of diagnosis and treatment (DT) phase	Relies on variables A-D defined above.	Assumed 2 months to recover from surgery, and 6 months' duration of chemotherapy.	Disaggregated by receipt of surgery alone (k=1) or surgery plus chemotherapy (k=2).	For k=1: minimum of [C + 2 months, A-4 months, B]<br><br>For k=2, minimum of [C+D+6 months, A-4 months, B]	Nil	n/a	n/a
Duration of R phase	Duration of remission (R) phase	Relies on three durations defined above.	Assumed cured if 8 years post-diagnosis of colon cancer.	No	Residual time from 8 years post-diagnosis, minus time in DT phase and any time in PT or T phases.	Nil	n/a	n/a
Cancer Disability Weights (DW)								

VARIABLE NAME	VARIABLE DEFINITION	SOURCE	DERIVATION FROM SOURCE & APPLICATIO N TO MODEL	HETEROGENEITY BY AGE/SEX/ ETHNICITY/ DEPRIVATION	EXPECTED VALUE	UNCERTAINTY INTERVAL (95% UI)	DISTRIBUTION	SENSITIVITY of ICER to this variable[1]
DT DW	Disability weight for the diagnosis and treatment phase	Global Burden of Disease (GBD) 2010,[7] Begg et al 2007[8]	Merged GBD 2010 DWs (given for cancers overall) with the colorectal cancer DWs in Begg 2007. See text below and BODE[3] protocol.[9]	No	0.288 (applies for different time lengths depending on k^{th} strata above)	Nil	n/a	n/a
PT DW	Disability weight for the pre-terminal phase	Global Burden of Disease (GBD) 2010,[7] Begg et al 2007[8]	Merged GBD 2010 DWs (given for cancers overall) with the colorectal cancer DWs in Begg 2007. See text below and BODE[3] protocol.[9]	No	0.539	Nil	n/a	n/a
T DW	Disability weight for the terminal phase	Global Burden of Disease (GBD) 2010,[7] Begg et al 2007[8]	Merged GBD 2010 DWs (given for cancers overall) with the colorectal cancer DWs in Begg 2007. See text below and BODE[3] protocol.[9]	No	0.548	Nil	n/a	n/a

VARIABLE NAME	VARIABLE DEFINITION	SOURCE	DERIVATION FROM SOURCE & APPLICATION TO MODEL	HETEROGENEITY BY AGE/SEX/ ETHNICITY/ DEPRIVATION	EXPECTED VALUE	UNCERTAINTY INTERVAL (95% UI)	DISTRIBUTION	SENSITIVITY of ICER to this variable[1]
R DW	Disability weight for the remission phase	Global Burden of Disease 2010,[7] Begg et al 2007[8]	Merged GBD 2010 DWs (given for cancers overall) with the colorectal cancer DWs in Begg 2007. See text below and BODE[3] protocol.[9]	No	0.167 (discounted at 20% per annum from end of year 1 post-diagnosis)	Nil	n/a	n/a
Effect of CCC on increasing receipt of chemotherapy								
Prop surg only baseline →surg and chemo	Proportion shifted from receiving surgery only to surgery + chemotherapy	Goodwin et al 2003[10] and expert estimates	See text below	No	0.33	0.09 to 0.65	Beta distribution (mean 0.33, s.d. 0.15)	59%

VARIABLE NAME	VARIABLE DEFINITION	SOURCE	DERIVATION FROM SOURCE & APPLICATION TO MODEL	HETEROGENEITY BY AGE/SEX/ ETHNICITY/ DEPRIVATION	EXPECTED VALUE	UNCERTAINTY INTERVAL (95% UI)	DISTRIBUTION	SENSITIVITY of ICER to this variable[1]
HR for chemo	Effect of chemotherapy with oxaliplatin on breast cancer mortality<br><br>(Effect of chemotherapy without oxaliplatin on breast cancer mortality considered as scenario analysis)	Sargent et al 2009[11] De Gramont et al 2007[12] Andre et al 2004[13]	See text below. Product of two HRs: 1: effect of chemo without oxaliplatin compared to no chemo multiplied by 2: effect of chemo with oxaliplatin compared to without oxaliplatin	No	1: 0.72<br>2: 0.78	1: 0.61 to 0.85<br>2: 0.63 to 0.98	Log normal<br>1: mean of logs minus 0.33, s.d. of logs 0.05<br><br>2: mean of logs minus 0.2435, s.d. of logs 0.05	21%
Effect of CCC on reducing wait times to treatments								
↓ in days to surgery	Proportionate reduction in days to surgery due to a CCC	Haideri et al 2011[14] and expert estimates	See text below	No	0.20	0.03 to 0.48	Beta distribution (mean 0.20, s.d. 0.121)	5%
↓ in EMR per day ↓in time from diagnosis to surg	Reduction in cancer excess mortality per day decrease in time from diagnosis to surgery (i.e. the effect of getting surgery faster on colon cancer mortality)	No direct evidence. Estimated using protocol[3], Whyte et al 2011[15], Tappenden et al 2007[16]	See text below	No	0.9972 ratio decrease in excess mortality rate per day quicker to surgery	0.9955 to 0.9987	Log normal (mean of logs minus 0.0028, s.d. of logs 0.0008	6%

VARIABLE NAME	VARIABLE DEFINITION	SOURCE	DERIVATION FROM SOURCE & APPLICATIO N TO MODEL	HETEROGENEITY BY AGE/SEX/ ETHNICITY/ DEPRIVATION	EXPECTED VALUE	UNCERTAINTY INTERVAL (95% UI)	DISTRIBUTION	SENSITIVITY of ICER to this variable[1]
↓ *days to chemotherapy*	Proportionate reduction in average days from surgery to chemotherapy due to a CCC	Expert estimates	See text below	No	0.20	0.03 to 0.48	Beta distribution (mean 0.20, s.d. 0.121)	63%
↓ *EMR per day ↓in time from surg to chemo*	Reduction in cancer excess mortality per day decrease in time from surgery to chemotherapy (i.e., the effect of getting chemotherapy faster on colon cancer mortality)	Biagi et al 2011[17]	See text below	No	0.9953 ratio decrease in excess mortality rate per day quicker from diagnosis to initiating chemo	0.9938 to 0.9969	Log normal (mean of logs minus 0.0047, s.d. of logs 0.0008)	43%
Effect of a CCC on reducing colon cancer morbidity								
↓*DW due to CCC*	Reduction in disability weight during diagnosis and treatment phase due to a CCC reducing patient anxiety	Ferrante et al 2008[18]	See text below	No	0.67	0.45 to 1.0	Log normal mean of logs minus 0.04, s.d. of logs 0.05	7%
Costs								

VARIABLE NAME	VARIABLE DEFINITION	SOURCE	DERIVATION FROM SOURCE & APPLICATIO N TO MODEL	HETEROGENEITY BY AGE/SEX/ ETHNICITY/ DEPRIVATION	EXPECTED VALUE	UNCERTAINTY INTERVAL (95% UI)	DISTRIBUTION	SENSITIVITY of ICER to this variable[1]
Baseline health system costs per month	Routine health system costs per month (without a CCC) varying by time	Health Tracker (linked NZ health datasets)	See Additional File 5	Yes by sex and age. Disaggregated by receipt of surgery alone (k=1) or surgery plus chemotherapy (k=2).	See Additional File 5	Nil	n/a	n/a
Incremental CCC cost from diagnosis to surgery	Incremental cost of CCC programme from provisional diagnosis to surgery (difference in costs for pathway of care with CCC minus pathway of care in business-as-usual comparator)	Consultatio n with local health care professiona ls (costed based on average salaries + 50% overheads)	See Additional File 4	No	$64.03 per patient	$29.42 to $98.64	Normal (mean 64.03, s.d. 17.66)	6%

VARIABLE NAME	VARIABLE DEFINITION	SOURCE	DERIVATION FROM SOURCE & APPLICATION TO MODEL	HETEROGENEITY BY AGE/SEX/ ETHNICITY/ DEPRIVATION	EXPECTED VALUE	UNCERTAINTY INTERVAL (95% UI)	DISTRIBUTION	SENSITIVITY of ICER to this variable[1]
Incremental CCC cost from surgery to start of chemotherapy	Incremental cost of CCC programme from surgery to start of chemotherapy (difference in costs for pathway of care with CCC minus pathway of care in business-as-usual comparator)	Consultation with local health care professionals (costed based on average salaries + 50% overheads)	See Additional File 4	No	$5.00 per patient	$-10.39 to $20.39	Normal (mean 5.00, s.d. 7.85)	2%
Cost of chemotherapy per patient	Cost per patient of 12 cycles of chemotherapy with oxaliplatin over 6 months	Bottom-up costing approach including cost of pharmaceuticals, outpatient attendance and overheads.	See Additional File 5	No	$17,811.78 per patient	$14,494.69 to $21,390.41	Gamma (mean 17,811.78, s.d. 1781.18)	40%

VARIABLE NAME	VARIABLE DEFINITION	SOURCE	DERIVATION FROM SOURCE & APPLICATIO N TO MODEL	HETEROGENEITY BY AGE/SEX/ ETHNICITY/ DEPRIVATION	EXPECTED VALUE	UNCERTAINTY INTERVAL (95% UI)	DISTRIBUTION	SENSITIVITY of ICER to this variable[1]
Incremental dietitian costs	Additional costs from dietitian referrals precipitated by a CCC	Expert estimates	Dietitian referrals estimated to increase by 50% (2 contacts per referral. See Additional File 4	No	$115.89 per patient	$81.38 to $141.16	Gamma (mean 115.9, s.d. 11.6)	4%
Incremental social worker costs	Additional costs from social worker referrals precipitated by a CCC	Cancer Institute NSW Report 2011[19] and Expert estimates	Social worker referrals estimated to increase by 42%, 6 contacts per referral. See Additional File 4.	No	$403.10 per patient	$327.95 to $483.97	Gamma (mean 403, s.d. 40.3)	13%

Heterogeneity in the baseline model

Stage III colon cancer rates by demographic strata

Elsewhere we describe how cancer incidence rates by socio-demographic groups were estimated[20], and the SEER stage distribution by sex, deprivation and ethnicity[3]. For this paper, we first estimated the incidence of colon cancer (previous estimates were for colorectal cancer combined) in 2011 by sex, age and ethnic group, by using a logistic regression model on NZCR data (1996 to 2008) to predict the odds (and thence the proportion) of colorectal cancer cases that were colon cancer. The regression model included main effects for sex, age (centred on 62.5, and modelled as a linear term), ethnicity (Māori and non-Māori), deprivation (deciles 1-3, 4-7 and 8-10), and calendar year.

Next, we estimated the proportion of all colon cancer cases that were stage III cancer cases, using data from the Differential Colon Cancer Survival by Ethnicity in New Zealand project (www.uow.otago.ac.nz/coloncancer-info.html) [5]. This was done by using a second logistic regression model on observations with complete staging data. The dependent variable was stage III (compared to 'rest'), and the independent variables were as above. The final model coefficients are also shown in Table 2 below.

Table 2: Logistic regression coefficients (and 95% confidence intervals) for models predicting the odds of being colon cancer and stage III colon cancer

	Model predicting colon cancer among all colorectal cancer cases ‡	Model predicting stage III colon cancer among all colon cancer cases $
**Variable**	**Coefficient (95% CI)**	**Coefficient (95% CI)**
Intercept	0.405 (0.359 to 0.452)	-0.124 (-0.638 to 0.383)
Sex (female = ref)	0.476 (0.438 to 0.513)	-0.374 (-0.728 to -0.021)
Age†	0.012 (0.010 to 0.014)	-0.004 (-0.019 to 0.011)
Ethnicity (non-Māori = ref)	-0.322 (-0.433 to -0.210)	-0.191 (-0.590 to 0.205)
Dep 4-7 (Dep 1-3 = ref)	0.003 (-0.042 to 0.048)	-0.283 (-0.787 to 0.226)
Dep 8-10 (Dep 1-3 = ref)	-0.048 (-0.098 to 0.002)	-0.309 (-0.814 to 0.203)
Diagnosis year*	0.003 (0.000 to 0.005)	0.025 (-0.055 to 0.104)
Sample size	51156	589

Dep = neighbourhood deprivation decile
† Centred at 62.5 years of age. ‡Model used 1996 onwards colorectal cancer cases from the cancer registry.
$ Modelled used data from the Differential Colon Cancer Survival by Ethnicity in New Zealand project where TNM staging was determined by notes review. *Diagnosis year is centred at 2003.

The intercept of the first logistic regression model gives the estimate of the odds of 1.501 (i.e. exp[0.405]) for colon cancer to non-colon cancer among all colorectal cancers for the reference individual, i.e. age 62.5, female, non-Māori, Deprivation deciles 1 to 3, diagnosis year 2003. As a proportion of all colorectal cancers, this is 1.501/ (1+1.501) = 0.600.

The second model then estimates the odds (and hence the proportion) of all colon cancer cases that were stage III in 2003 by joint strata of sex, age and ethnicity (Table 2; we assume this 2003 proportion applies to the 2011 baseline data). These proportions were then multiplied together, then multiplied into 2011 estimated colorectal cancer incidence rates to get 2011 estimated colon cancer rates, and then again into the estimated population counts in 2011 to give counts for 2011 (Table 3).

Table 3: Estimated stratum specific proportions of colorectal cancer cases that are colon cancer, and colon cancer cases that are stage III, and estimated rates and counts of stage III colon cancer (estimates for deprivation deciles 4-7 presented only)

Age groups	Males		Females	
	**Māori**	**Non-Māori**	**Māori**	**Non-Māori**
Proportions of colorectal cancer that are colon cancer				
45-49	0.593	0.668	0.476	0.556
50-54	0.608	0.681	0.490	0.570
55-59	0.622	0.694	0.505	0.585
60-64	0.636	0.706	0.520	0.599
65-69	0.649	0.719	0.535	0.613
70-74	0.663	0.730	0.550	0.627
75-79	0.676	0.742	0.564	0.641
80-84	0.689	0.753	0.579	0.655
85-89	0.701	0.764	0.593	0.668
90+	0.713	0.774	0.607	0.681
Proportions of colon cancer that are stage III				
45-49	0.288	0.329	0.37	0.416
50-54	0.284	0.324	0.365	0.410
55-59	0.279	0.319	0.360	0.405
60-64	0.275	0.315	0.355	0.400
65-69	0.271	0.310	0.350	0.395
70-74	0.267	0.306	0.345	0.390
75-79	0.262	0.301	0.341	0.385
80-84	0.258	0.297	0.336	0.380
85-89	0.254	0.292	0.331	0.375
90+	0.250	0.288	0.326	0.370
Rates (per 100,000)				
45-49	4.2	4.8	3.7	5.5
50-54	7.8	9.1	6.4	9.6
55-59	14.9	17.7	11.3	17.0
60-64	27.8	33.5	19.3	29.5
65-69	48.9	59.6	32.2	50.1
70-74	77.9	96.3	51.3	80.9
75-79	102.1	128.4	66.3	106.0
80-84	115.6	147.8	79.5	128.8
85-89	115.8	147.4	80.2	129.6
90+	116.0	147.3	80.8	130.4
Counts (rates applied to projected 2011 census population)				
45-49	0.26	2.64	0.25	3.19
50-54	0.42	4.68	0.38	5.21
55-59	0.59	8.04	0.48	8.15
60-64	0.85	14.50	0.61	13.54
65-69	0.96	20.28	0.69	18.15
70-74	1.14	26.66	0.82	24.74
75-79	0.81	25.86	0.65	25.07
80-84	0.50	22.58	0.44	26.04
85-89	0.16	11.97	0.19	17.79
90+	0.03	3.05	0.06	6.58

The cross-classified data in Table 3 was used to specify the heterogeneity distribution, of any sex by age by ethnic group by deprivation.

Stage III colon cancer rates and counts by socio-demographic and baseline clinical strata

Having estimated the stage III colon cancer rates and counts by demographic strata, there is one more step to establish the baseline (i.e. pre-intervention) set of rates, namely to disaggregate further by receipt of surgery and chemotherapy.[2]

Regarding pre-existing data, Hill et al found that 95.8% of Māori with colon cancer (95% CI 93.1-98.5) were offered surgery versus 96.2% (95%CI 94.0-98.5) of non-Māori. They also found that 0.7% (95% CI 0.2-2.3) of Māori and 0.8% (95% CI 0.3-2.2) of non-Māori decline surgery[21]. Given the small numbers of people not receiving surgery, we simply assume that everyone received surgery.

Determining the distribution of surgery only and both surgery and chemotherapy receipt was conducted by fitting a logistic regression model to just the stage III colon cancer data set. The same specification of independent variables was used as above for the logistic regression model predicting the odds of stage III colon cancer. The output is shown in Table 4 below.

[2] Consideration was given to including comorbidity as a predictor of receipt, and indeed as separate strata in the model. However, this was deemed unnecessary; the method we outline in the sections below captures the mortality difference between the receipt categories below due to comorbidities (in addition to the treatment effect per se), but only models the treatment effect of either surgery or chemotherapy as part of the intervention. Thus it was possible to not explicitly include comorbidity in the model, improving model parsimony.

Table 4: Logistic regression coefficients (and 95% confidence intervals) for model predicting the odds of being either surgery, compared to both surgery and chemotherapy.

**Variable**	**Surgery only cf both surgery and chemotherapy**
Intercept	-1.938 (-3.019 to -0.949)
Sex (female = ref)	-0.122 (-0.800 to 0.551)
Age†	0.112 (0.077 to 0.152)
Ethnicity (non-Māori = ref)	0.991 (0.216 to 1.808)
Dep 4-7 (Dep 1-3 = ref)	0.363 (-0.611 to 1.356)
Dep 8-10 (Dep 1-3 = ref)	0.412 (-0.564 to 1.41)
Diagnosis year*	-0.220 (-0.388 to -0.061)
Sample size	189

Model used data from the Differential Colon Cancer Survival by Ethnicity in New Zealand project where TNM staging was determined by notes review.
† Centred at 62.5 years of age
*Diagnosis year is centred at 2003.

Exponentiating the intercept of the model gives the odds of surgery, to surgery and chemotherapy of 0.144 for the reference patient, or a proportion of 0.144/1.144=0.126. This regression output was then used to estimate the stratum specific proportions, and multiplied into the rates and counts in Table 3 to generate rates and counts by baseline clinical strata as shown in Table 5.

Table 5: Estimated stratum specific proportions of baseline-receipt of treatment (strata j: 1=surgery, 2=surgery and chemo) among stage III colon cancer patients (for base year 2011; estimates for deprivation deciles 4-7 presented only)

	**Males**				**Females**			
	**Māori**		**Non-Māori**		**Māori**		**Non-Māori**	
**Age groups**	**j=1**	**j=2**	**j=1**	**j=2**	**j=1**	**j=2**	**j=1**	**j=2**
Proportions								
45-49	0.0804	0.920	0.0314	0.969	0.089	0.910	0.0353	0.965
50-54	0.133	0.867	0.0536	0.946	0.147	0.853	0.0602	0.940
55-59	0.211	0.789	0.0902	0.910	0.232	0.768	0.101	0.899
60-64	0.318	0.682	0.148	0.852	0.345	0.655	0.164	0.836
65-69	0.449	0.551	0.232	0.768	0.480	0.520	0.255	0.745
70-74	0.588	0.412	0.346	0.654	0.617	0.383	0.374	0.626
75-79	0.714	0.286	0.481	0.519	0.738	0.262	0.511	0.489
80-84	0.813	0.187	0.618	0.382	0.831	0.169	0.646	0.354
85-89	0.884	0.116	0.739	0.261	0.896	0.104	0.762	0.238
90+	0.930	0.070	0.832	0.168	0.938	0.062	0.848	0.152

The cross-classified data in Table 5 was used to sample in the Monte Carlo inner loop whether patients had surgery only or surgery and chemotherapy.

Clinical transition time: diagnosis to surgery; surgery to chemotherapy

For the micro-simulation trials of individual variability, it is necessary to have a range of times to sample from for each of: diagnosis to surgery; and surgery to beginning of chemotherapy. These are estimable from the colon cancer study data (http://www.otago.ac.nz/wellington/research/cancercontrol/projects/otago019908.html) [5].

Figure 2: Histogram of time from diagnosis to surgery (excluding those with zero days (22.6%) due to diagnosis at surgery or emergency presentation) for 146 stage III colon cancer patients

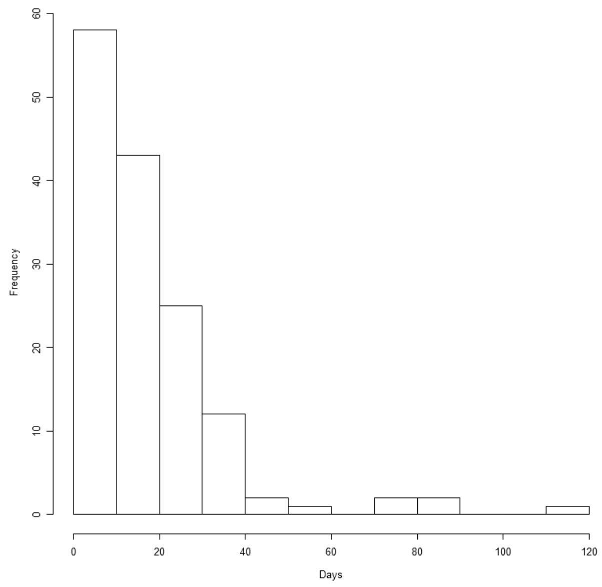

Source: data from the Differential Colon Cancer Survival by Ethnicity in New Zealand

Figure 2 above shows a simple histogram of time to surgery from the colon cancer dataset; the distribution is highly right-skewed. A logistic regression was run on those 22.6% with zero days from diagnosis to surgery (prior to excluding them) and no statistical significance was found for age, sex, ethnicity, deprivation or diagnosis year.

Figure 3 below shows a simple histogram of time from surgery to receipt of chemotherapy; the distribution is also skewed, but with a clearer mode rather than a near-exponential distribution of the time to surgery.

Figure 3: Histogram of time from surgery to chemotherapy for 98 stage III colon cancer patients

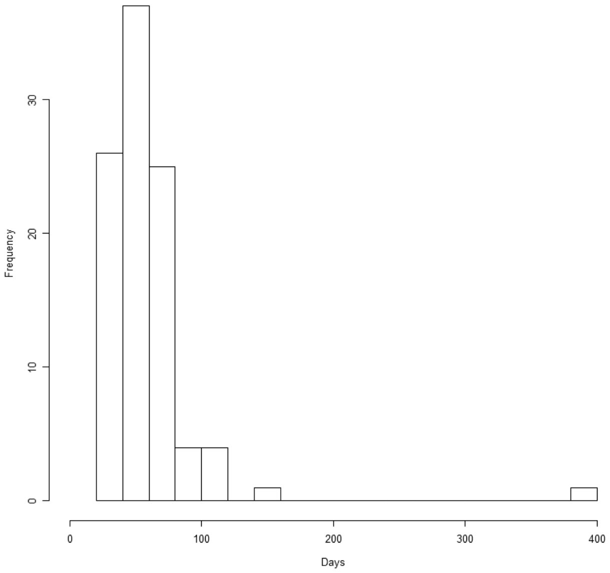

Source: data from the Differential Colon Cancer Survival by Ethnicity in New Zealand

Two gamma regression models were run for time to surgery and then time from surgery to chemotherapy, with predictors of sex, age group and ethnicity. (Deprivation was not included as it was not significant. We also tried including a coefficient for the number of days from diagnosis to surgery in the second model, so that dependencies can be explicitly modelled. However, it was non-significant and dropped from the final model.) Results are shown in Table 6 below.

Table 6: Gamma regression coefficients (and 95% confidence intervals) for a log-link function model predicting the number of days from diagnosis to surgery, and from surgery to receipt of chemotherapy.

	Coefficient (95% CI)	
**Variable**	**Days from diagnosis to surgery**	**Days from surgery to chemotherapy**
Intercept	2.714 (2.368 to 3.079)	3.995 (3.708 to 4.293)
Sex	0.271 (-0.026 to 0.57)	0.099 (-0.154 to 0.354)
Age†	0.014 (0.000 to 0.027)	0.006 (-0.006 to 0.018)
Ethnicity (Māori cf non-Māori)	0.303 (-0.011 to 0.62)	0.293 (0.024 to 0.565)
Diagnosis year *	0.065 (-0.006 to 0.136)	0.036 (-0.03 to 0.10)
Sample size	146	98
Random error variance	0.826	0.400

The model used data from the Differential Colon Cancer Survival by Ethnicity in New Zealand project where TNM staging was determined by notes review, and excludes those with missing data on any covariates and no receipt of surgery and no receipt of chemotherapy (respectively).

† Centred at 62.5 years of age

*Diagnosis year is centred at 2003.

Time to surgery and time to chemotherapy were modelled as stochastic uncertainty in the economic decision model, and in order to retain heterogeneity by socio-demographic strata we created CDFs for each possible socio-demographic group. It was simplest to operationalise in TreeAge Pro using parametrically specified gamma distributions for each socio-demographic group. Therefore, we conducted simulations in R using the above regression equations to produce 10000 predictions for each possible combination of sex by age by ethnicity. (Note that these simulations included random draws from the error term distribution as well as the distribution about each coefficient, generating an individual-level distribution of times to event – not 'just' the average or expected value in each socio-demographic strata.) The alpha and beta parameters, and average days, are shown in Table 7 below.

Table 7: Estimated stratum specific gamma distribution for time to surgery and time from surgery to chemotherapy (alphas, beta (average days))

Age group	Males		Females	
	Non-Māori	Māori	Non-Māori	Māori
Time to surgery from diagnosis				
45-49	1.08, 15.3 (17)	1.16, 19.2 (22)	1.09, 11.5 (13)	1.15, 14.6 (17)
50-54	1.15, 15.1 (17)	1.16, 20.5 (24)	1.15, 11.4 (13)	1.17, 15.3 (18)
55-59	1.14, 16.5 (19)	1.16, 22.0 (26)	1.15, 12.2 (14)	1.14, 16.6 (19)
60-64	1.14, 17.5 (20)	1.15, 23.5 (27)	1.18, 13.0 (15)	1.22, 16.9 (21)
65-69	1.17, 18.4 (22)	1.12, 25.6 (29)	1.13, 14.5 (16)	1.16, 19.2 (22)
70-74	1.14, 19.9 (23)	1.17, 26.6 (31)	1.18, 15.2 (18)	1.16, 20.5 (24)
75-79	1.13, 21.7 (25)	1.20, 27.3 (33)	1.14, 16.4 (19)	1.12, 22.6 (25)
80-84	1.16, 23.1 (27)	1.11, 32.3 (36)	1.13, 17.8 (20)	1.12, 24.2 (27)
85-89	1.12, 25.3 (28)	1.05, 36.5 (38)	1.14, 19.3 (22)	1.12, 26.1 (29)
90+	1.11, 26.7 (30)	1.10, 36.2 (40)	1.13, 19.7 (22)	1.13, 26.7 (30)
Time from surgery to chemotherapy				
45-49	4.13, 13.4 (55)	4.38, 16.9 (74)	4.25, 11.9 (51)	4.47, 15.1 (67)
50-54	4.31, 13.2 (57)	4.38, 17.7 (78)	4.37, 11.8 (52)	4.53, 15.2 (69)
55-59	4.42, 13.4 (59)	4.42, 17.9 (79)	4.48, 11.8 (53)	4.73, 15.1 (71)
60-64	4.54, 13.4 (61)	4.40, 18.3 (81)	4.53, 12.1 (55)	4.55, 16.1 (73)
65-69	4.56, 13.7 (62)	4.44, 18.7 (83)	4.46, 12.6 (56)	4.58, 16.2 (74)
70-74	4.50, 14.3 (64)	4.35, 20.0 (87)	4.58, 12.7 (58)	4.52, 17.3 (78)
75-79	4.47, 15.0 (67)	4.18, 21.6 (90)	4.51, 13.2 (60)	4.35, 18.6 (81)
80-84	4.29, 15.9 (68)	4.07, 22.5 (92)	4.20, 14.8 (62)	4.19, 20.0 (84)
85-89	4.13, 17.2 (71)	4.04, 23.6 (95)	4.14, 15.6 (65)	3.92, 21.9 (86)
90+	4.12, 17.6 (73)	3.82, 25.4 (97)	3.96, 16.7 (66)	3.85, 23.0 (89)

By way of visualising these distributions, four examples using the above alpha and beta values are shown in Figure 4. These gamma distributions were then converted to CDFs for sampling from in the DES.

Figure 4: Estimated gamma distributions using output in Table 7 for:

Time to surgery, non-Māori 55-59 yr. female

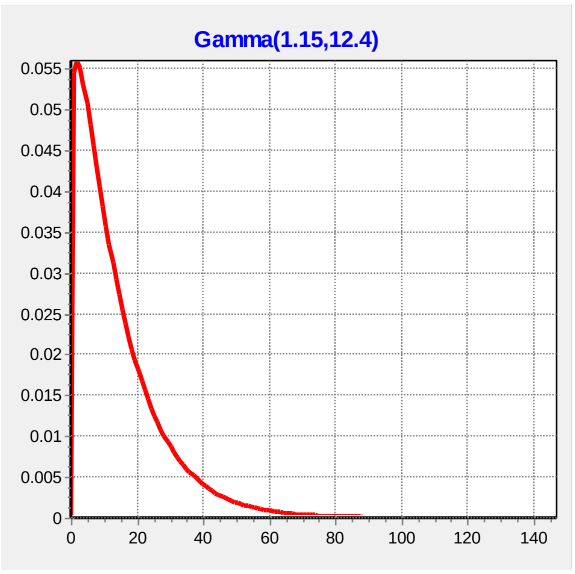

Time to surgery, Māori 70-74 yr. male

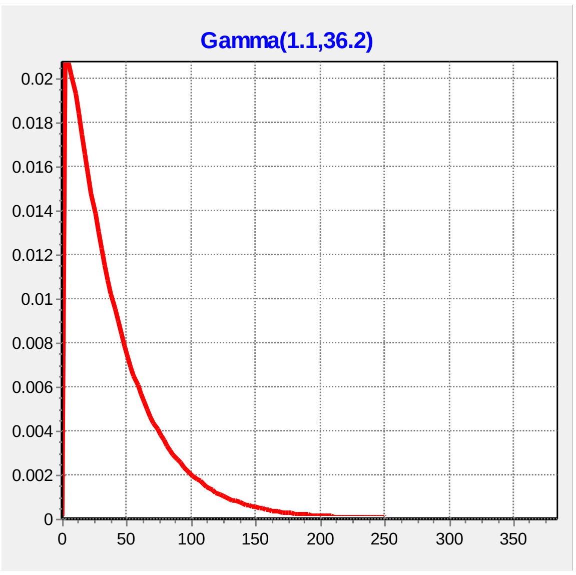

Surg to chemo, non-Māori 55-59 yr. female

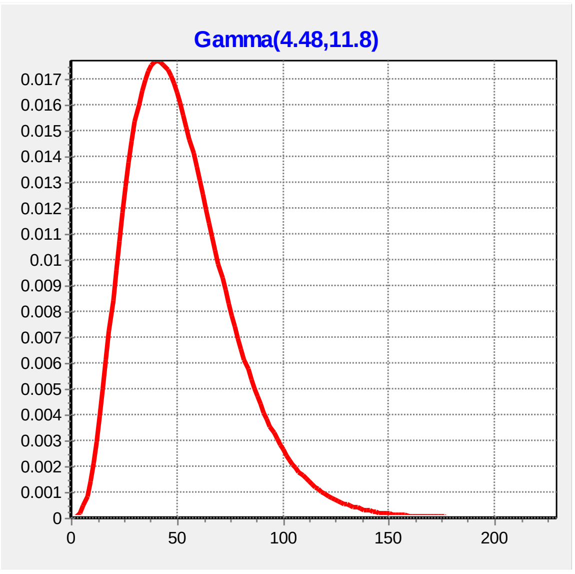

Surgery to chemo, Māori 70-74 yr. male

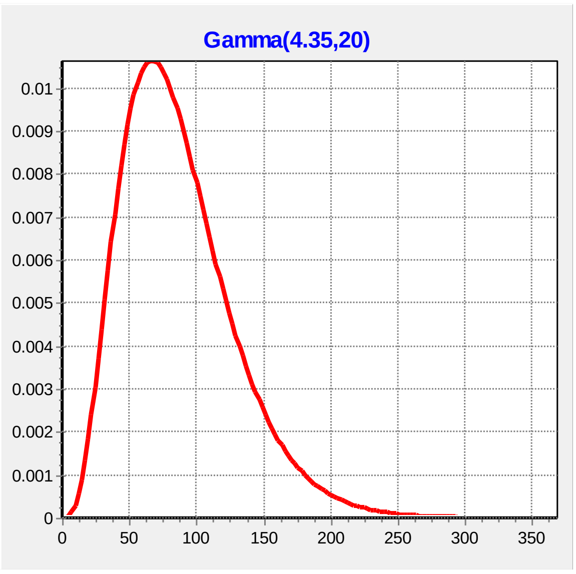

Excess mortality rates by socio-demographic (i) and clinical (j) strata

We first estimated the excess mortality rate (EMR; cancer consequent mortality rate[22]) for stage III colon cancer by the socio-demographic strata (but not yet the clinical strata). Elsewhere, we have used excess mortality rate modelling, with cubic splines to model mortality by month post-diagnosis,

for SEER regional cancer, by socio-demographics for cases diagnosed from 1994-2008 with mortality follow-up up to December 2010[3]. This modelling also included a term for calendar year that predicted a 3.5% per annum reduction in EMRs (presumably due to improved management and treatment); we assumed this trend continued to our base-year of 2011.

For the microsimulation, we converted the EMR functions into cumulative EMR (CEMR) functions (see Figure 5 below). Where the CEMR asymptotes give the fraction of cases dying from their cancer (or one minus the proportion cured). For example, over 60% of Māori females aged 75 years and older die from their colon cancer (allowing for competing mortality risk), but fewer New Zealand European do. In the microsimulation, each trial involves a random draw from a uniform 0/1 distribution. Using older Māori females, a random draw of 0.70 means she survived the colon cancer (if surviving the competing other causes of death), and a random draw of 0.2 means that she will die within the first year of colon cancer – at a given number of days post diagnosis in the baseline at least.

Figure 5: Cumulative distribution of time of death from cancer, P(T<t), used to sample time to death from cancer in the discrete event simulation, for females, NZDep 4-7.

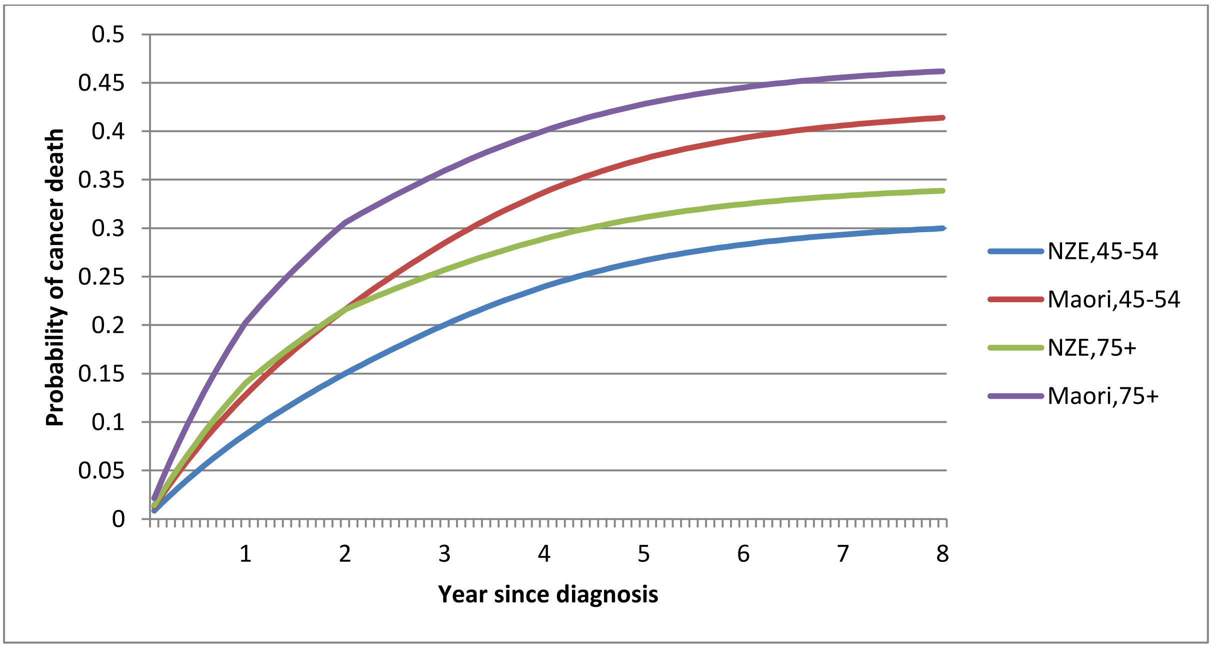

NZE = New Zealand European

The *shape* of the EMR curve (being the slope of the above CDF curves) post-diagnosis varies by stage. Among those with distant or advanced stage the EMR is high at the outset, but among those with regional or local cancer it is initially low then increases. Ideally, we would like to know the shape of the EMR curve for the stage III subset of SEER regional cancers. However, it would be challenging to do so, and probably not particularly influential on final outputs – so we assume that the shape of the EMR curve for SEER regional cases is the same for stage III. Second, analyses on the colon cancer study data (http://www.otago.ac.nz/wellington/research/cancercontrol/projects/otago019908.html)[5] failed to show any statistically significant differences in hazard ratios between stages IIa, IIb and III (which are all subsumed in the SEER regional category). Thus, we simply use the EMRs for SEER regional as direct estimates for stage III.

Thus, we now have EMR for stage III by socio-demographic strata, i. We then disaggregated further by clinical strata, j, using the following approach. A Cox proportional hazards model on the New Zealand colon cancer data was run, but now restricted to just stage III and adjusting for sex, age, ethnicity and deprivation (deciles 1-3, 4-7 and 8-10 grouped), to determine hazard ratios for surgery only (j=1) compared to both surgery and chemotherapy (j=2). Then the EMR for each socio-demographic i acted as a weighted average of the EMRs for each clinical strata j, where the weights are the proportion of colon cancer cases in each j stratum:

$$EMR[III]_i^t = EMR[III]_{i,j=2}^t \times HR[j=1:j=2] \times P[j=1|III]_i + EMR[III]_{i,j=2}^t \times P[j=2|III]_i$$

$$\xrightarrow{yields} EMR[III]_{i,j=2}^t = \frac{EMR[III]_i^t}{P[j=2|III]_i + (HR[j=1:j=2] \times P[j=1|III]_i)}$$

Where:

$EMR[III]^t_{i,j}$	= the excess mortality rate for clinical strata j within socio-demographic strata i at time t post diagnosis
$HR[j = 1 : j = 2]$	= hazard ratio for clinical stratum j=1 compared to j=2 (from Cox proportional hazards model), assumed uniform across socio-demographic stratum as 2.30 (95%CI 1.37 ‑ 3.86); note, however, that we do not model uncertainty in the HR).
$P[j = 1,2 \| III]_i$	= proportion of colon cases in socio-demographic stratum i that are in one of clinical strata j=1 or 2 (see Table 5)

Figure 6: Kaplan Meier curves showing survival function for patients who received surgery and chemotherapy (red line; j=2) and surgery only (green; j=1) with 95% confidence intervals.

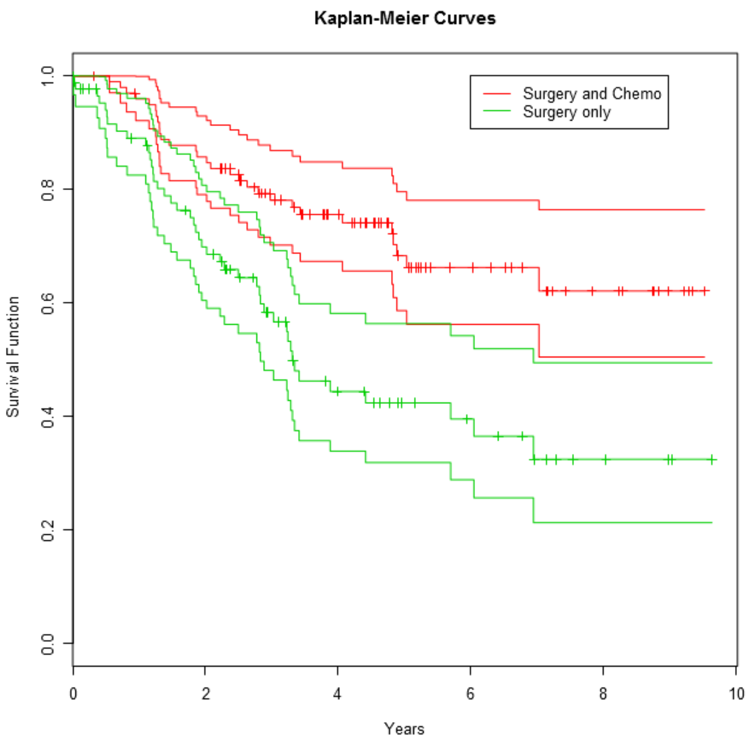

Having calculated $EMR[III]^t_{i,j=2}$ it is then straight forward to calculate $EMR[III]^t_{i,j=1}$ using the hazard ratios.

Morbidity

We utilised morbidity or disability weights (DW) from the Global Burden of Disease 2010 study[7] , and mathematically integrated these into our cancer models (i.e. the average of DWs across all cancers was forced to be that in the GBD)[9]. These assume a morbidity weight of 0.288 for a 9 month diagnosis and treatment (DT) state, and 0.167 for remission for colorectal cancer (see Table 1 in main paper). Note, however, that for this economic decision model that explicitly models an intervention that reduces time through phases, the duration of the DT phase will be that predicted for time to surgery plus time to start of chemotherapy plus six months (the latter fixed as the time for course of chemotherapy). If dying from colon cancer, the person is assumed to have a month in a terminal state (DW=0.548) and three months prior to that in a pre-terminal state (DW=0.539). Note that we do not explicitly and mathematically model transitions into these states, but rather we model time to death from cancer (and then allow for these fixed times in pre-terminal and terminal states in the mathematical calculation of QALYs^{DW}).

In addition to the disease model morbidity weights, we also allow for expected background population morbidity by taking the average total disability in the population at each sex, age and ethnic combination from the New Zealand BDS (personal communication, Dr Martin Tobias, Ministry of Health, 2012). This value or 'average morbidity weight' is sometimes referred to as the prevalent years of live with disability (pYLD); we will use this nomenclature. If, for example, the pYLD was 0.10 for a given sex, age and ethnic group, and a person was in a terminal state, then the total disability is neither 0.10 + 0.548 = 0.648, nor 'just' 0.548. Rather, it is 1 – (1-0.548) × (1-.01) = 0.5932. The derivation and values of these pYLDs are described elsewhere.[9]

Intervention parameters

The proportion of people receiving surgery only in the comparator who additionally receive chemotherapy with the intervention

Regarding receipt of adjuvant chemotherapy, the oxaliplatin-based regimen FOLFOX (folinic acid (leucovorin) + fluorouracil (5-FU) + oxaliplatin) is recommended by the New Zealand Guidelines Group for stage III colon cancer[23]. However, it is likely there is a moving trend to prescribing capecitabine with oxaliplatin using the CAPOX (also known as XELOX) regimen over FOLFOX due to its predominantly oral route of administration (personal communication with clinical nurse specialist, medical oncologist, and oncology pharmacist separately). Both CAPOX and FOLFOX have the same efficacy as both are fluorouracil based however their toxicity profiles differ[24].

The only contraindications to receiving FOLFOX are: known hypersensitivity, pregnant patients, debilitated patients and patients with severe renal impairment (creatinine clearance <30mL/min) [25-28]. Precautions for prescribing FOLFOX include: mild renal impairment, hepatic impairment, pernicious anaemia and being 70 years of age or older and female. Factors that will delay treatment with chemotherapy post-operatively include: post-operative complications such as sepsis (8.9% occurrence in Māori vs 6.7% in non-Māori), pneumonia (7.2% occurrence in Māori vs 4.9% in non-Māori)[21] and bone marrow suppression which is unlikely unless the patient is receiving immunosuppressant therapy (or previous chemotherapy recently).

Therefore, comorbidities may be a valid reason for some people to not receive chemotherapy, but not to the extent of the reported low coverage[29]. Note this is the most recent estimate available on coverage of chemotherapy for colon cancer patients in New Zealand however we expect coverage to have improved since the implementation of multi-disciplinary team meetings. Based on the information provided by Medsafe for each chemotherapy agent and discussions with one of our clinical advisors (medical oncologist), we assume that an excess of 95% of people aged up to 65 who

receive surgery should be eligible for chemotherapy, with eligibility falling to 90% for those aged 65-69, 85% for those aged 70-74, 80% for those aged 75-79 and 75% for those aged 80 and above.

This places a limit on the potential increase in people receiving chemotherapy with the intervention. We accommodate this in the model by first calculating the number of people in stratum j=1 that remain as eligible. For example, consider 80-84 year old Māori males in Table 5. In business-as-usual, 81.3% do not receive chemotherapy and 18.7% do. The assumptions in the above paragraph are that 75% are actually eligible, or 25% ineligible. Therefore, 81.3% - 25% = 56.3% of Māori males aged 80-84 are estimated to be eligible for chemotherapy, but did not receive it. We return to this example below, after considering the evidence of the effect of CCC on increasing uptake among this 56.3%.

In order to estimate the effect size of a CCC programme on increasing coverage of chemotherapy in cancer patients a systematic literature search was conducted in Ovid MEDLINE (R), the Database of Abstracts of Reviews of Effects, the HTA (health technology assessment) database, the Cochrane Library, the New Zealand Ministry of Health publications and NHS EED (NHS Economic Evaluation Database). The explicit search strategy, including keywords, MeSH terms and the inclusion and exclusion criteria are documented in Additional File 3. Of the 317 systematic reviews retrieved from databases, 293 were excluded after relevance screening and the remaining 24 were excluded for not meeting our inclusion criteria as they did not have suitable outcomes to answer our research question. One paper [10] was retrieved from 'snowballing' from a systematic review (not produced by our systematic search) on case management[30]. This paper is not a systematic review and therefore did not meet our inclusion criteria in this regard however it meets our inclusion criteria in all other aspects (i.e. a suitable intervention to address our research question).

We are unable to find any other studies that have looked at a nurse-led intervention changing coverage of chemotherapy following surgery. We have found studies measuring impact of nurse-led care on coverage of palliative care services and hospices but these do not meet our inclusion criteria as they are evaluating a different point in the care pathway.

The Goodwin et al study [10] measured the effect of a CCC type intervention on improving receipt of chemotherapy for patients with breast cancer. The intervention in this study is similar to the intervention we have specified (although more intensive) and had no other system-wide intervention being introduced simultaneously. The study was of high internal validity although the results were borderline statistically significant. It is a randomised prospective trial for women aged 65 or older, newly diagnosed with breast cancer, who received nurse case management for 12 months post diagnosis in the intervention arm compared to usual care in the control arm. The study was conducted in two public hospitals and 13 community hospitals in Texas, North America.

Women with stage 3 and 4 breast cancer were more likely to receive chemotherapy in the nurse case management group compared to the control group (72.7% versus 30%, p-value 0.056)[10]. In other words, this is 61% of the woman not receiving chemotherapy in the absence of care coordinators actually receiving chemotherapy with nurse case management [1- (27.3/70)]. Overall for women of all stages of breast cancer those in the intervention arm were less likely to receive inappropriate treatment than the control arm (16.9% versus 26.2%, p-value 0.061) [10].

As study participants were over the age of 65 and predominantly of low income status we would expect to see less of an improvement in chemotherapy coverage with the nurse case management intervention at a general population level. The case management intervention was also more intensive than the CCC intervention we are modelling as it included home visits and monthly telephone calls to patients.

Taking into account the chemotherapy referral process in New Zealand (including centres with and without multi-disciplinary team meetings) and discussions with local health care professionals (medical oncologist, colorectal surgeon, colorectal nurses and oncology nurses from different district health boards in New Zealand) our best estimate is an increase in 10-70% of those eligible to receive adjuvant chemotherapy receiving it with the CCC programme than would not otherwise. We estimate the effect size to be between 10-50% for most patients and only up to 70% in a minority of

patients. This as shown in Figure 7 using a beta distribution with alpha=3 and beta=6, giving a mean of 0.33, standard deviation of 0.15 (95% UI 0.09 to 0.65).

We assumed that this parameterisation was the same across all socio-demographic strata, as the role of the CCC here is to ensure patients are discussed at the multi-disciplinary team meeting (where these exist) post operatively and that referrals are appropriately completed (in order to minimise delays) and reach the oncology department in a timely manner.

Figure 7: Beta distribution (assigned by authors following review of evidence and discussion with experts) for effect size of improved coverage of chemotherapy among eligible non-refusers.

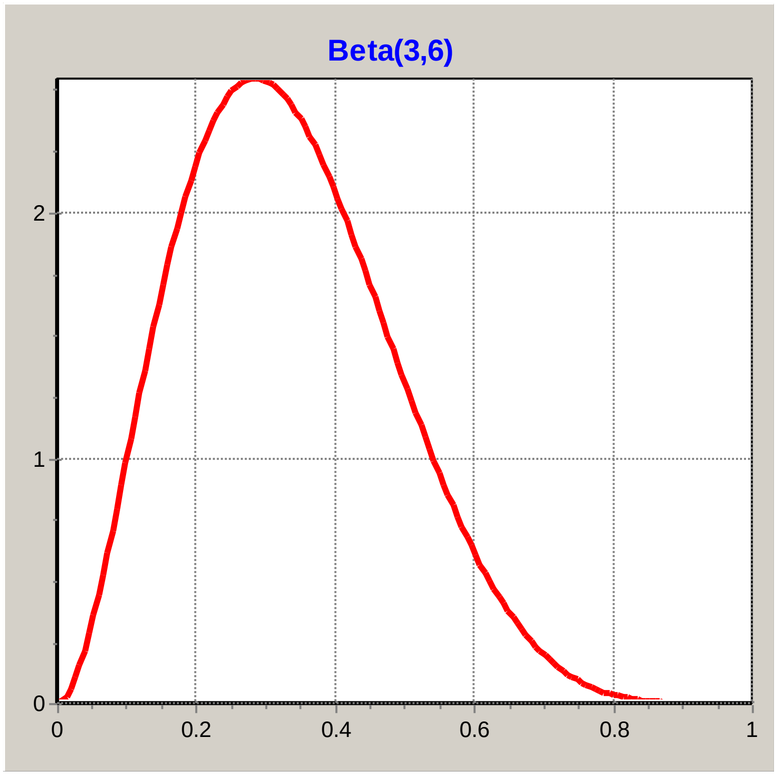

Returning to our example above of 80-84 year old male Māori, this (best estimate; will actually be parameter with uncertainty as above) 33% increase in the uptake of chemotherapy applies to the 56.3% who did not receive chemotherapy in the baseline, but we estimate were actually eligible. Thus, our best estimate is that 33% × 56.3% = 18.8% of all 80-84 year old male Māori are shifted from surgery only, to surgery and chemotherapy (i.e. P[k=2|i=Māori/male/80-84] = 0.188). Or

conditional on already being in the j=1 strata only receiving surgery, P[k=2|i=Māori/male/80-84 ,j=1] = 0.188 / 0.813 = 0.231.

Excess mortality rates under intervention: increasing coverage of chemotherapy

Part of the CCC effect is by increasing coverage of chemotherapy, which lowers the EMR (or increases survival). However, the gain from receiving chemotherapy will probably not be as large as the observed difference in EMRs between j=1 and j=2, as this also includes some difference in EMR due to background characteristics that influenced treatment decisions in the absence of the intervention. (That is, the observed difference in EMR between those people receiving surgery only and people receiving both surgery and chemotherapy will be due to *both* the 'true' treatment effect of chemotherapy and confounding whereby people with (say) higher comorbidities were both less likely to receive chemotherapy and had worse cancer survival.) Therefore, we could not use existing data to estimate$EMR[III]^{t}_{i,j=1,k=2}$. Rather, we used the best estimate from the literature of the hazard ratio reduction in mortality for chemotherapy treatment among stage III colon cancer patients (assumed constant across socio-demographic strata and time following receipt of chemotherapy), and multiplied this by $EMR[III]^{t}_{i,j=1}$ to generate $EMR[III]^{t}_{i,j=1,k=2}$. The best estimate from the literature (to our knowledge) is given by Sargent et al[11]. Pooling 18 randomised trials, they estimate a hazard ratio of *overall survival* of 0.74 (no confidence interval given in the paper, but estimated as 0.64 to 0.86*).*

However this study only analysed trials testing fluorouracil-based adjuvant therapy (predominantly fluorouracil plus leucovorin) without the addition of oxaliplatin as present in the FOLFOX regimen currently used in New Zealand (and costed in this analysis). We thus incorporated the additional gain in overall survival by using oxaliplatin in the FOLFOX4 regimen[12] in overall sensitivity analysis. The MOSAIC trial gave a hazard ratio for recurrence (at the median follow-up of 37.9 months) in the group given fluorouracil plus leucovorin (FL) plus oxaliplatin, as compared to the FL group, as 0.77 (95%CI 0.65 to 0.91; p=0.002) corresponding to a 23% reduction in the risk of relapse [13]. Patients

in the MOSAIC trial followed beyond the 3 year cut-off showed the probability of surviving at 6 years with FOLFOX versus LV as 68.3% versus 72.9% with a hazard ratio of 0.80 (0.66, 0.98) [12].

We model excess and other mortality rates (and consequent transition probabilities) separately – the EMRR for receipt of chemotherapy will therefore be less again. For example, by the end of the first year of follow-up the EMR for colon cancer is about 0.10 per person per year for a 60-64 year old. Averaging across sexes and ethnic groups, the background annual mortality rate at this age is about 0.01 – a total mortality rate of 0.11. Assume about two thirds of colon cancer patients were receiving chemotherapy, and disregard variations in background mortality for now by receipt of chemotherapy. Let 'X' be the EMR of those not receiving chemotherapy. Therefore, $0.11 = 0.33 (X+0.01) + 0.67\times0.74 (X+0.01)$, meaning $X = 0.123$. For an overall survival hazard ratio of 0.74 (i.e. the Sargent et al estimate, excluding oxaliplatin), then $0.74 = (0.01 + EMRR \times 0.123) / (0.01 + 0.123)$, meaning the EMRR is $0.719 \approx 0.72$. Thus, we estimate the EMRR for receipt of chemotherapy (excluding oxaliplatin) as 0.72 with a shifted 95% confidence interval of 0.61 to 0.85 using the same formulas. Likewise, we estimated the additional EMRR for oxaliplatin as $0.784 \approx 0.78$, with a shifted confidence interval of 0.63 to 0.98.

Proportionate reduction in average days to surgery; $\Delta[Dx \rightarrow Surg_i]$

In order to find the effect size of a CCC intervention in improving timeliness of care from provisional diagnosis to surgery and from surgery to chemotherapy for cancer patients a systematic literature search was conducted in Ovid MEDLINE (R), the Database of Abstracts of Reviews of Effects, the HTA database, the Cochrane Library, the New Zealand Ministry of Health publications and NHS EED. The search strategy and findings are documented in Additional File 3. Of the 511 records retrieved from the six databases, 476 were excluded after relevance screening and a further 43 were excluded for not meeting our remaining inclusion criteria.

Fourteen studies were found via 'snowballing' and 'information foraging' that measured the impact of nurse-led interventions on improving timeliness of care; two from systematic reviews produced

by our systematic search and the remaining from 'information foraging'. Ten of these 14 were excluded for not meeting our inclusion criteria. Of the remaining four, three were identified as having poor internal validity[31-33], leaving only one study to inform our effect size estimate[14].

The remaining study was a retrospective case series analysis using historical controls showing a 21% reduction in the median days from initial presentation to first treatment [14]. The intervention evaluated was similar to our specified intervention including the typical supportive and coordination roles, however study participants were also provided with cell phones, taxi passes and child care services.

The study participants were a targeted population not generalisable to the general population as they were identified by health care providers as being most in need of patient navigation and thus more likely to have more complex clinical pictures or experiencing barriers of access to care. In this regard the improvement in timeliness of care for this population is likely an underestimate for the general population.

Based on this study, an understanding of the functioning of the New Zealand healthcare system, and discussions with health professionals from different district health boards (medical oncologist, colorectal surgeon, oncology nurses) we estimate that the proportionate reduction in average days from diagnosis to surgery is 20%. We parameterised this conservative best estimate as a beta distribution (Figure 8) with alpha=2 and beta=8, giving a mean of 0.2 and SD of 0.121 (95% UI 0.03 to 0.48). We have deliberately specified this with considerable uncertainty due to the lack of robust published evidence to support our estimate. We assumed this parameter to be the same across socio-demographic strata.

Figure 8: Beta distribution assigned by authors to effect size of: decreasing time from provisional diagnosis to surgery; decreasing time from surgery to chemotherapy

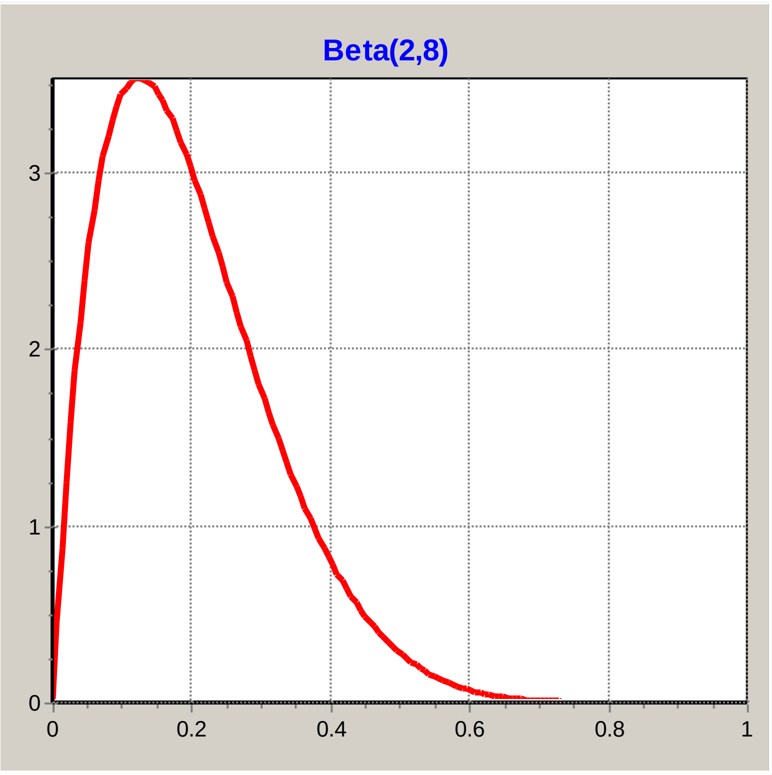

Proportionate reduction in average days to chemotherapy; $\Delta[Surg \rightarrow Chemo_i]$

A systematic literature search was conducted in Ovid MEDLINE (R), the Database of Abstracts of Reviews of Effects, the HTA database, the Cochrane Library, the New Zealand Ministry of Health publications and NHS EED to find CCC interventions that improve timeliness of care across the care pathway. The search strategy is documented in Additional File 3. From this search no studies were found to measure the effect of a CCC type intervention on the time from surgery to chemotherapy.

One study was found via ‘snowballing’ to measure the effect of a CCC intervention on the timeliness of care from surgery to adjuvant therapy in cancer patients. This study was the same retrospective case series analysis discussed earlier under the parameter ‘proportionate reduction in average days to surgery’[14]. The patient navigator intervention in this study showed no reduction in time from surgery to adjuvant therapy for breast cancer patients.

After discussion with local clinical experts we do not estimate a null effect of CCCs on reducing the time from surgery to adjuvant chemotherapy for colon cancer patients in the New Zealand context. Discussions with key informants working within the surgical and oncology public healthcare services of New Zealand suggests CCCs could play a major role in improving the efficiency of the referral process between the surgical and oncology teams postoperatively. This would be achieved by the CCC ensuring no patients are missed out at the surgical multi-disciplinary team meeting discussions, ensuring timely discussions for all patients at these meeting (as soon after their lymph node biopsy results as possible) as well as timely and appropriately completing referrals to oncology and acting on any delays to referrals being received. In hospitals where no multi-disciplinary team meetings exist or other organised triaging and referral system, CCCs are likely to have more of an impact on improving timely initiation of adjuvant chemotherapy post-operatively. The CCC role will be unable to improve current waiting lists for chemotherapy.

With the current time period from surgery to initiating chemotherapy in New Zealand being on average 4-8 weeks [21, 34] we estimate that the proportionate reduction in average days from surgery to start of chemotherapy with the CCC intervention to be 20%. We parameterised our best estimate as the same beta distribution as for time to surgery **Error! Reference source not found.**with alpha=2 and beta=8, giving a mean of 0.2 and SD of 0.121 (95% UI 0.03 to 0.48). As above, we have deliberately specified this with considerable uncertainty due to the lack of strong evidence available. We assumed this parameter to be the same across socio-demographic strata.

Reduction in cancer excess mortality per day decrease in time to surgery; EMRR [Dx→Surgery/day]

A structured literature search was carried out in Ovid MEDLINE (R) 1946 to April 2012 and Google Scholar to identify studies that investigated whether a change in the time from diagnosis to surgery impacted on survival. The following Medical Subject Headings were used: ‘neoplasms’, ‘colon’, ‘colorectal neoplasms’, ‘general surgery’, ‘time factors’, ‘survival’, ‘survival analysis’ and ‘mortality’.

Other keyword search terms used were: ‘cancer’, ‘colorectal’, ‘treatment’, ‘surgery’, ‘timeliness’, ‘timeframe’, ‘delay’ and ‘clinical outcomes’.

It is well established that diagnosing cancer earlier improves survival; however this is dependent on diagnosing the cancer at an earlier stage. Results from observational studies looking at the relationship between earlier treatment and survival in cancer are often counter-intuitive showing either no improvement or a negative association. As Neal describes this is likely to be due to studies failing to account for the speed of growth of tumours; more aggressive tumours present more quickly, are treated more quickly however endure worse outcomes[35]. Stapley confirms this notion by observing that in studies which adjust for emergency admissions as a confounding factor the negative associations seen between decreasing time to treatment and impact on survival in colorectal cancer cease to exist.[36]

In the absence of valid direct evidence of how survival improves with earlier surgery, we instead used the differences in EMRs between stages and the average number of days in a natural history model in each stage to calculate the relative increase in EMR with each extra day until diagnosed. Next, we assumed this relative increase in EMR with each subsequent day’s delay in diagnosis was the same as the relative increase in EMR for each subsequent day’s delay to imitating definitive treatment, namely surgery. This is described in more detail below.

1. As published elsewhere, the EMRRs in New Zealand comparing SEER extent of disease regional to local colon cancer is 4.26, and distant compared to local is 26.8[3].
2. Whyte et al (2011)[15] have published 95% confidence limits for annual transition probabilities from Duke stage A to B (0.73 to 0.93; assuming a logit normal distribution this gives 0.90 as the central estimate), from Dukes B to C (0.72 to 0.94; 0.86), and from Dukes C to D (0.61 to 0.92; 0.81). Tappenden et al (2007) in forerunner work to the Whyte et al paper also give the annual transition probability from Dukes D to death from cancer of 0.39[16].

For the central estimates, these correspond to rates per person year of 2.34, 1.99, 1.66 and 0.49, respectively.

3. The average duration in each stage, assuming an exponential distribution of times, is 1/exit rate, or 0.43, 0.50, 0.60 and 2.05 years in each of Dukes stage A, B, C and D, respectively.
4. Dukes A is roughly equivalent to SEER extent of disease 'local', B and C to Regional, and D to distant. Therefore average times in local, regional and distant SEER stages (in a natural disease history model) might be 0.43, 1.10 and 2.05 years, respectively.
5. Using these average times, one can plot them against the ln(EMRR) by stage, and calculate the slope of the regression line joining the three points. For the central estimates, this slope is a 0.0028 unit increase in ln(EMRR) per day of the natural history progression through the stages. Using the lower confidence limits of the days in each stage this slope estimate was 0.0011, and using the upper confidence limits 0.0041. These regression estimates using the lower and upper confidence limits on the one hand are probably overestimates (by assuming 1.0 correlation in uncertainty in the average days in each stage), and on the other hand underestimates due to not including uncertainty in the EMRRs themselves. Thus, and given the (necessary) assumptions in this method, we simply scale up this uncertainty interval by 50%, giving our final estimates of 0.0028 (95% UI 0.0011 to 0.0045) for the ln(EMRR) per day of delayed diagnosis. Exponentiated, this is a 1.0028 ratio increase in the EMR per day delay in diagnosis. We extrapolate this to also apply to delays in days to surgery and conversely it's inverse to days quicker to surgery (ending up with EMRR per day quicker to surgery of 0.9972, with 95% UI 0.9955 to 0.9987).

Are these estimates plausible? Given the relatively rapid growth and transit times between stages, and the very large increases in EMR with stage, yes. However, it must be emphasised that these are not empirical estimates, and assume an exponential distribution of the rate between stages. This may be adequate for calculations about the average duration times. However, it is much more likely that the actual distribution of rates over time is Gamma, but we have insufficient data from the two

source papers to parameterise that. (However, for a range of possible Gamma distributions that generated a cumulative probability after one year of a similar magnitude to those reported by Whyte et al, we found that the average number of days in each stage was usually within +/-10% to 20% of that from an exponential distribution assumption.)

Reduction in cancer excess mortality per day decrease in time to chemo; EMRR [Dx→ Chemo/day]

A structured literature search was carried out in Ovid MEDLINE (R) 1946 to April 2012 and Google Scholar to investigate the relationship between reducing the time to initiating chemotherapy following surgery in stage III colon cancer and survival. The following Medical Subject Headings were used: ‘neoplasms’, ‘colon’, ‘colorectal neoplasms’, ‘therapeutics’, ‘time factors’, ‘survival’, ‘survival analysis’ and ‘mortality’. Other keyword search terms used were: ‘cancer’, ‘colorectal’, ‘treatment’, ‘therapy’, ‘timeliness’, ‘timeframe’, delay’, ‘interruption’ and ‘clinical outcomes’.

The highest quality evidence found measuring the impact of timeliness of chemotherapy on survival is a meta-analysis of ten studies showing statistically significant decreases in overall survival and disease-free survival associated with an increase in time between surgery and chemotherapy initiation in colon cancer patients stage II and III[17]. A decrease in overall survival (HR 1.14 95% CI 1.10-1.17) and disease-free survival (HR 1.14 95% CI 1.10-1.18) were associated with an increase in four weeks between surgery and initiation of adjuvant chemotherapy. The unclear inclusion criteria and assessment of studies to be included in the meta-analysis and the potential for bias in their search strategy warrants the outcomes of this study to be treated with caution, however the evidence provided is the best available to use for this input parameter in our model.

This study has also been criticised for being biased by confounding factors such as age and comorbidities and that poorer survival associated with starting chemotherapy later could be due to those with comorbidities recovering more slowly from surgery[37], however studies were only included in the meta-analysis that adjusted for prognostic factors. The authors defend their findings by demonstrating that the overall and cancer specific survival reveal a similar effect size.

Based on this increase in overall mortality of 14% for every four week delay in initiation of adjuvant chemotherapy we estimate the excess mortality rate ratio (EMRR) per day less from diagnosis to initiating chemotherapy to be 0.9953 (i.e. (1/1.14)^(1/28) with a 95% UI 0.9938 to 0.9969. On the log normal scale, the estimates are -0.0047, -0.0062 to -0.0031. We assumed this parameter to be the same across socio-demographic strata.

Proportionate reduction in disability weight

A systematic literature search was conducted in Ovid MEDLINE (R), the Database of Abstracts of Reviews of Effects, the HTA database, the Cochrane Library, the New Zealand Ministry of Health publications and NHS EED to identify studies that measured the impact of CCC type interventions on quality of life. The search strategy and findings are documented in Additional File 3. Of the 381 systematic reviews retrieved from databases, 361 were excluded after relevance screening and a further 20 were excluded for not meeting our remaining inclusion criteria (predominantly as they measured patient satisfaction rather than a recognised quality of life indicator and thus had unsuitable outcomes to address our research question). This left no relevant papers from our systematic search of systematic reviews that met our inclusion criteria.

22 papers were retrieved from ‘snowballing’ and ‘information foraging’ and of these three were excluded on relevance and a further 15 were excluded for not meeting our inclusion criteria, predominantly for the intervention not being similar enough to the one we are modelling, particularly the point in the patient care pathway. Of the four remaining papers that did meet our inclusion criteria; one study showed a significant improvement in quality of life with a CCC type intervention, the other three showed improvements in quality of life measures but none statistically significant. All the studies used internationally recognised quality of life measurement tools however only one of these was compatible to converting the change in quality of life to a change in disability weight using the EQ-5D[18]. This study also happened to be evaluating an intervention most similar to that being modelled here.

The Ferrante et al study (which was identified, from hand searching the reference list of one of the systematic reviews excluded) measured changes in anxiety on the Zung Anxiety Self-Assessment Scale at the time of an abnormal mammogram and one month after final resolution of diagnosis[18]. The mean anxiety index in the control arm of breast cancer patients started within normal range (mean 36.5, SD 6.9) and then increased to the mild anxiety range (mean 50.3, SD 15.6) in the follow-up stage. For the breast cancer patients who had patient navigation their baseline anxiety score was higher initially in the mild anxiety range (mean 41.9, SD 14.7) which reduced to within the normal anxiety range in the follow-up phase (mean 32.9, SD 12.2). Thus the apparent effect of care coordinators was 50.3 – 32.9 = 17.4 for a cross-sectional post-randomisation comparison, or ([41.9-32.9] - [36.5-50.3]) = 22.8 for a difference in pre-post changes in control and interventions arms. The average effect size is therefore about 20 points.

Previous work has shown composite scores of the EQ-5D anxiety domain were significantly correlated with the composite scores of the Zung anxiety scale (r=0.590; p<0.001).[38] Similarly the scores on the EQ-5D visual analogue scale were significantly correlated with the composite scores of the Zung' anxiety scale (r=0.564; p<0.001). Therefore, we next assume that a one category shift on the Zung Anxiety scale is equivalent to a one point shift in the anxiety domain of the EuroQol (EQ-5D) scale i.e. EQ-5D index score 11111 (perfect health) to 11112 (moderate anxiety but otherwise perfect health) or from 11112 to 11113 (severe anxiety but otherwise perfect health).

We assume the Zung categories 'normal' and 'mild' anxiety equate to the EQ-5D anxiety categories 'normal' and 'moderate' and that the Zung categories 'severe' and 'extreme' anxiety equate to the EQ-5D category of 'severe' anxiety.

The utility score of colon cancer stage III treated with resection and chemotherapy without significant side effects has been found to be 0.7 (0.63 to 0.77) [39].

Valuations of EQ-5D health states for the New Zealand population have previously been constructed for 245 EQ-5D states[40]. The adequacy of EQ-5D for Māori and non-Māori New Zealanders has been investigated showing that the EQ-5D takes into account how Māori perceive health as much as non-Māori[41]. Using the publicly available EQ-5D index calculator online for New Zealand showed that a change in anxiety state from one category to another with all other EQ-5D domains remaining unchanged equates to about a 0.1 change in utility index score. Therefore, we equate the effect of CCC to a 0.1 shift in EQ-5D score.

Following Higashi and Barendregt [42], we set the 'effect' of CCC on quality of life as:

$$Effect^{Care\ Coord} = \frac{1 - colon\ utility + 0.1}{1 - colon\ utlity} = \frac{1 - (0.7 + 0.1)}{1 - 0.7} = 0.67$$

We have no empirical data upon which to directly calculate an uncertainty interval about this effect. We assume that a zero effect (i.e. RR 1.0) is possible, but unlikely, and actually sets the 97.5th percentile. Therefore, the s.d. on the ln[RR] scale is (ln[1]-ln0.67])/1.96 = 0.20, giving a 95% uncertainty interval of 0.45 to 1.0. We allow a wide uncertainty interval to take into consideration the three other studies which found non-significant improvements in quality of life.

References

1. Sarfati D, Blakely T, Pearce N: **Measuring cancer survival in populations: relative survival vs cancer-specific survival**. *Int J Epidemiol* 2010:dyp392.
2. Koerkamp BG, Stijnen T, Weinstein MC, Hunink MGM: **The Combined Analysis of Uncertainty and Patient Heterogeneity in Medical Decision Models**. *Medical Decision Making* 2011, **31**(4):650-661.
3. Blakely T, Costilla R, Soeberg M: **Cancer Excess Mortality Rates Over 2006-2026 for ABC-CBA**. In: *Burden of Disease Epidemiology, Equity, and Cost-Effectiveness Programme (BODE^3) Technical Report: Number 4* Wellington: Department of Public Health, University of Otago; 2012.
4. Kvizhinadze G, Blakely T: **Projected NZ Life Tables** In: *Burden of Disease Epidemiology, Equity and Cost-Effectiveness Programme (BODE3).* Wellington: University of Otago Wellington 2011.
5. **Differential Colon Cancer Survival by Ethnicity in New Zealand** [http://www.otago.ac.nz/wellington/research/cancercontrol/projects/otago019908.html]
6. Des Guetz G, Uzzan B, Morere J-F, Perret G, Nicolas P: **Duration of adjuvant chemotherapy for patients with non-metastatic colorectal cancer**. *Cochrane database of systematic reviews* (1):CD007046.
7. Salomon JA, Vos T, Hogan DR, Gagnon M, Naghavi M, Mokdad A, Begum N, Shah R, Karyana M, Kosen S, Farje M, Moncada G, Dutta A, Sazawal S, Dyer A, Seiler J, Aboyans V, Baker L, Baxter A, Benjamin E, Bhalla K, Bin Abdulhak A, Blyth F, Bourne R, Braithwaite T, Brooks P, Brugha T, Bryan-Hancock C, Buchbinder R, Burney P, Calabria B, Chen H, al E: **Common values in assessing health outcomes from disease and injury: disability weights measurement study for the Global Burden of Disease Study 2010.** . *Lancet* 2012, **380**(9859):2129-2143.
8. Begg S, Vos T, Barker B, Stevenson C, Stanley L, Lopez A: **The burden of disease and injury in Australia 2003.** In. Canberra: AIHW; 2007.
9. Blakely T, Foster R, Wilson N: **Burden of Disease Epidemiology, Equity and Cost-Effectiveness (BODE3) Study Protocol** In: *Burden of Disease Epidemiology, Equity and Cost-Effectiveness Programme.* Wellington: University of Otago Wellington 2012.
10. Goodwin JS, Satish S, Anderson ET, Nattinger AB, Freeman JL: **Effect of nurse case management on the treatment of older women with breast cancer**. *J Am Geriatr Soc* 2003, **51**(9):1252-1259.
11. Sargent D, Sobrero A, Grothey A, O'Connell MJ, Buyse M, Andre T, Zheng Y, Green E, Labianca R, O'Callaghan C, Seitz JF, Francini G, Haller D, Yothers G, Goldberg R, de Gramont A: **Evidence for cure by adjuvant therapy in colon cancer: observations based on individual patient data from 20,898 patients on 18 randomized trials**. *Journal of clinical oncology : official journal of the American Society of Clinical Oncology* 2009, **27**(6):872-877.
12. de Gramont A, Boni C, Navarro M, Tabernero J, Hickish T, Topham C, Bonetti A, Clingan P, Lorenzato C, André T: **Oxaliplatin/5FU/LV in adjuvant colon cancer: Updated efficacy results of the MOSAIC trial, including survival, with a median follow-up of six years.** *Journal of Clinical Oncology* 2007, **25**(18S).
13. Andre T, Boni C, Mounedji-Boudiaf L, Navarro M, Tabernero J, Hickish T, Topham C, Zaninelli M, Clingan P, Bridgewater J, Tabah-Fisch I, de Gramont A: **Oxaliplatin, fluorouracil, and leucovorin as adjuvant treatment for colon cancer**. *N Engl J Med* 2004, **350**(23):2343-2351.
14. Haideri N, Moormeier J: **Impact of Patient Navigation from Diagnosis to Treatment in an Urban Safety Net Breast Cancer Population**. *Journal of Cancer* 2011, **2**(467-473).

15. Whyte S, Walsh C, Chilcott J: **Bayesian Calibration of a Natural History Model with Application to a Population Model for Colorectal Cancer.** *Medical decision making : an international journal of the Society for Medical Decision Making* 2011, **31**:625-641.
16. Tappenden P, Chilcott J, Eggington S, Patnick J, Sakai H, Karnon J: **Option appraisal of population-based colorectal cancer screening programmes in England**. *Gut* 2007, **56**(5):677-684.
17. Biagi JJ, Raphael MJ, Mackillop WJ, Kong W, King WD, Booth CM: **Association between time to initiation of adjuvant chemotherapy and survival in colorectal cancer: a systematic review and meta-analysis**. *Jama* 2011, **305**(22):2335-2342.
18. Ferrante JM, Chen PH, Kim S: **The effect of patient navigation on time to diagnosis, anxiety, and satisfaction in urban minority women with abnormal mammograms: a randomized controlled trial**. *J Urban Health* 2008, **85**(1):114-124.
19. Cancer Institute NSW: **Evaluation of the role of Cancer Care Coordinator. Sydney: Cancer Institute NSW**. In. New South Wales Cancer Institute NSW; 2011.
20. Costilla R, Atkinson J, Blakely T: **Incorporating Ethnic and Deprivation Variation to Cancer Incidence Estimates over 2006-2026 for ABC-CBA.** In. Wellington: Department of Public Health, University of Otago; 2011.
21. Hill S, Sarfati D, Blakely T, Robson B, Purdie G, Dennett E, Cormack D, Dew K, Ayanian JZ, Kawachi I: **Ethnicity and management of colon cancer in New Zealand: do indigenous patients get a worse deal?** *Cancer* 2010, **116**(13):3205-3214.
22. Sarfati D, Blakely T, Pearce N: **Measuring cancer survival in populations: relative survival vs cancer-specific survival.** *International Journal of Epidemiology* 2010(dyp392).
23. New Zealand Guidelines Group: **Management of early colorectal cancer. Evidence-based Best Practice Guideline.** In. Wellington Ministry of Health; 2011.
24. Ducreux M, Bennouna J, Hebbar M, al e: **Capecitabine plus oxaliplatin (XELOX) versus 5-fluorouracil/leucovorin plus oxaliplatin (FOLFOX-6) as first-line treatment for metastatic colorectal cancer.** *International Journal of Cancer* 2011, **128**(3):682-690.
25. Medsafe: **Xeloda**. In. Edited by Medsafe: Medsafe; 2011.
26. Medsafe.: **DBL Leucovorin Calcium Injection USP ad Tablets** In. Edited by Medsafe: Medsafe 2007.
27. Medsafe.: **DBL Oxaliplatin for Injection**. In. Edited by Medsafe: Medsafe; 2008.
28. Medsafe.: **Fluorouracil: Fluorouracil Injection BP 25 mg/ml**. In. Edited by Medsafe: Medsafe; 2011.
29. Cunningham R, Sarfati D, Hill S, al e: **Colon cancer management in New Zealand: 1996-2003.** *New Zealand Medical Journal* 2009, **122**(1294):51-60.
30. Wulff CN, Thygesen M, Sondergaard J, al e: **Case management used to optimize cancer care pathways: a systematic review.** *BMC Health Services Research* 2008, **8**(227).
31. Donelan K, Mailhot JR, Dutwin D, Barnicle K, Oo SA, Hobrecker K, Percac-Lima S, Chabner BA: **Patient perspectives of clinical care and patient navigation in follow-up of abnormal mammography**. *J Gen Intern Med* 2011, **26**(2):116-122.
32. Koh C, Nelson JM, Cook PF: **Evaluation of a patient navigation program.** *Clinical Journal of Oncology Nursing* 2011, **15**(1):41-48.
33. Schwaderer KA, Proctor JW, Martz EF, Slack RJ, Ricci E: **Evaluation of Patient Navigation in a Community Radiation Oncology Center Involved in Disparities Studies: A Time-to-Completion-of-Treatment Study**. *Journal of Oncology Practice* 2008, **4**(5):220-224.
34. Hill S, Sarfati D, Blakely T, Robson B, Purdie G, Chen J, Dennett E, Cormack D, Cunningham R, Dew K, McCreanor T, Kawachi I: **Survival disparities in Indigenous and non-Indigenous New Zealanders with colon cancer: the role of patient comorbidity, treatment and health service factors**. *Journal of epidemiology and community health* 2010, **64**(2):117-123.
35. Neal RD: **Do diagnostic delays in cancer matter?** *British journal of cancer* 2009, **101**:S9-S12.

36. Stapley S, Peters TJ, Sharp D, Hamilton W: **The mortality of colorectal cancer in relation to the initial symptom at presentation to primary care and to the duration of symptoms: a cohort study using medical records**. *British journal of cancer* 2006, **95**(10):1321-1325.
37. Sargent D, Grothey A, Gray R: **Time to initiation of adjuvant chemotherapy and survival in colorectal cancer**. *Jama* 2011, **306**(11):1199; author reply 1200.
38. Klemenc-Ketis Z, Kersnik J, Eder K, al e: **Factors associated with health-related quality of life among university students.** *Srpski arhiv za celokupno lekarstvo* 2011, **139**(3-4):197-202.
39. Ness RM, Holmes AM, Klein R, Dittus R: **Utility valuations for outcome states of colorectal cancer**. *Am J Gastroenterol* 1999, **94**(6):1650-1657.
40. Devlin NJ, Hansen P, Kind P, Williams AH: **The health state preferences and logical inconsistencies of New Zealanders: A tale of two tariffs**. In: *Centre for Health Economics Discussion Paper 180.* Dunedin, New Zealand The University of York, Centre for Health Economics, and University of Otago, New Zealand; 2000.
41. Perkins MR, Devlin NJ, Hansen P: **Māori health state preferences: a pilot study** In: *Economics Discussion Papers Series.* Dunedin, New Zealand: Department of Economics

University of Otago; 2001.

42. Higashi H, Barendregt JJ: **Cost-effectiveness of total hip and knee replacements for the Australian population with osteoarthritis: discrete-event simulation model**. *PLoS One* 2011, **6**(9):e25403.
