# Supplementary material for: Cancer care coordinators in stage III colon cancer: a cost-utility analysis
Source: BMC Health Serv Res. 2015 Aug 5;15:306. doi: 10.1186/s12913-015-0970-5 (PMC4523949; doi:10.1186/s12913-015-0970-5)
Supplement: Additional file 3: — Documents the literature search strategies used. (PDF 764 kb) [file 12913_2015_970_MOESM3_ESM.pdf]

Additional File 3:
Literature search strategies
For ‘Cancer care coordinators in Stage III colon cancer: a cost-utility analysis’

Tony Blakely[1‡], Lucie Collinson[1], Giorgi Kvizhinadze[1], Nisha Nair[1], Rachel Foster[1], Elizabeth Dennett[2], Diana Sarfati[1]

[1]Burden of Disease Epidemiology, Equity, and Cost-Effectiveness (BODE[3]) Programme, Department of Public Health, University of Otago Wellington, PO Box 7343, Wellington, New Zealand

[2]Department of Surgery, University of Otago Wellington, PO Box 7343, Wellington, New Zealand

[‡]corresponding author

Additional File 3: Systematic literature search strategies for effect sizes of the cancer care coordinator (CCC) intervention

This file documents our systematic literature search strategies and findings for the three key input parameters for the effect size of the intervention; timeliness of care, coverage of chemotherapy and change in quality of life.

Table 1: 'Timeliness of care' systematic literature search for systematic reviews

**Literature Search Summary Form**	
**Date:** 02/08/2012	
**1. Research question defined**	
Do cancer care coordinators improve timeliness of care?	
P (population) = patients with cancer	
I (intervention/exposure and setting) = nurse-led care coordination intervention	
C (Comparisons) = baseline care with no care coordinator roles in place	
O (outcome) = improved timeliness of care	
T (timeframe) = from diagnosis of cancer to receiving treatment	
**2. Defined Inclusion and Exclusion Criteria**	
Inclusion	Exclusion
Nurse-led patient-level coordination intervention	Systems-level coordination intervention
Hospital based intervention	Community based intervention
Intervention matches our care coordinator intervention specification i.e. face to face contact with patient	Solely telephone-based interventions or other non-face to face patient contact interventions.
**Systematic reviews**	
All systematic reviews relevant to the research question published from 1946 onwards	Opinion pieces, non-systematic reviews
All other specific inclusion and exclusion criteria as defined above	
**3. Explicit search strategy**	
Keywords	Medical Subject Headings (MeSH terms)
coordinat* OR care coordinator* OR patient navigatio* OR nurse case manag* OR clinical nurse specialist OR nurse specialist OR nurse practitioner* OR case manage* OR key worker OR liaison officer	case management OR nurse clinicians OR nurse practitioners
Cancer	Neoplasms
Treatment OR therapy OR surgery OR chemotherapy	therapeutics OR general surgery OR drug therapy

time OR delay	time OR time factors
<u>Limits</u>	
English language. Humans. Reviews (maximises specificity)	
**4. Sources used**	
Medline	Cochrane library
DARE and HTA databases	Ministry of Health Publications
**Economic papers**	
NHS EED	
**5. Summary of search**	
**Systematic reviews**	
Number of records retrieved from databases	511
Number of additional records received from other sources	14
Total number of records retrieved after removal of duplicates	523
Number of records excluded by relevance screening	476
Number of articles excluded by exclusion criteria (i.e. one or more of the following):	43
o Unsuitable article type, e.g. non-systematic review or editorial	
o Unsuitable study population to address our research question	
o Unsuitable intervention/exposure to address our research question	
o Unsuitable outcome to address our research question	
o Updated or repetition of other similar review by same authors	
o Other (specify)	
Number of articles excluded by critical appraisal	3
Reasons: All three excluded by critical appraisal are non-randomised studies. Donelan et al presented patient reported outcomes (and thus likely to be biased as reliant on memory). Koh et al used historical controls with a small sample size (n=55) of predominantly employed patients with private health insurance. Schwaderer et al did not clearly describe baseline characteristics of the participants and the results lack internal validity, it is unclear whether the differences were influenced by a patient navigator or not.	
Total number of articles retained for inclusion	1

7

Search Results Table										
Database	Search string	No. of papers								
		Initial no. retrieved	Duplicates[a]	Total no. excl duplicates	Relevance screening of records		Application of Inclusion/Exclusion Criteria to paper		Critical appraisal (final selection)	
					Exclude	Retain	Exclude	Retain	Exclude	**Final**
**Systematic reviews**										
Medline 1946 to present (02/08/12)	1. coordinat*.mp. OR care coordinator*.mp. OR patient navigatio*.mp. OR nurse case manag*.mp. OR clinical nurse specialist.mp. OR nurse practitioner.mp. OR nurse specialist.mp. OR case manage*.mp. OR key worker.mp OR liaison officer.mp. OR Case Management/cl, ec, og [Classification, Economics, Organization & Administration] OR Nurse Clinicians/cl, ec, ed, og, st, sd [Classification, Economics, Education, Organization & Administration, Standards, Supply & Distribution OR Nurse Practitioners/cl, ec, ed, st [Classification, Economics, Education, Standards] [138176]<br>2. cancer.mp. or Neoplasms/ [977742]<br>3. Treatment.mp. or Therapeutics/ OR therapy.mp. **OR** *General Surgery/su [Surgery] OR chemotherapy.mp. or Drug Therapy/ [3659908]<br>4. Time/ or Time Factors/ or time.mp. OR delay.mp. [2463736]<br>5. 1 and 2 and 3 and 4 [452]<br>6. limit 5 to (English language and humans) [382]									

	7. limit 6 to “reviews (maximises specificity)” [5]	5	0	5	4	1	1	0	0	**0**
	8. limit 6 to "reviews (best balance of sensitivity and specificity)" [4]	4	0	4	3	1	1	0	0	**0**
	9. limit 6 to "review articles" [82]<br>10. remove duplicates from 9 [2]	82	2	80	72	8	6	2	1	**1**
NZ MoH (05/09/12)	Manual search: cancer care coordinators	286	0	286	267	19	19	0	0	**0**
Cochrane Library: Cochrane Database of Systematic Reviews, DARE, HTA Database and NHS EED 1946 -2012 (05/09/12)	1. (coordinat*) or (care coordinat*) or (patient navigation*) or (nurse case manage*) or (clinical nurse specialist) or (nurse specialist) or (nurse practitioner*) or (case manage*) or (key worker) or (liaison officer) [7122] *[Title, Abstract of Keywords]*<br>2. MeSH descriptor **Case Management,** this term only or MeSH descriptor **Nurse Clinicians,** this term only or MeSH descriptor **Nurse Practitioners,** this term only [969]<br>3. #1 or #2 [7198]<br>4. (cancer)[4198] *[Title, Abstract of Keywords]*<br>5. MeSH descriptor **Neoplasms,** this term only [4058]<br>6. #4 or #5 [47766]<br>7. (treatment) or (therapy) or (surgery) or (chemotherapy) [29956] *[Title, Abstract of Keywords]*<br>8. MeSH descriptor **Therapeutics**, this term only or MeSH descriptor **General Surgery**, this term only or MeSH descriptor **Drug Therapy**, this term only [738]<br>9. #7 or #8 [434231]<br>10. (time) or (delay) [4028] *[Title, Abstract of Keywords]*<br>11. MeSH descriptor **Time**, this term only or MeSH	128 (13 Reviews, 105 Trials, 10 Methods Studies)	0	128	126	2	2	0	0	**0**

	descriptor **Time Factors,** this term only [45052]<br>12. #10 or #11 [170595]<br>13. #3 and #6 and #9 and #12 [128]									
Snowballin g		0	0	0	0	0	0	0	0	**0**
Informatio n Foraging (google scholar and google)		6	0	6	0	6	4	2	0	**2**
**Total**		**511**	**2**	**509**	**472**	**37**	**33**	**4**	**1**	**3**
a Refers to duplicate detection of the same citation, either within the same database, or in different databases; ensure that one citation is retained, and other duplicates discarded. This does not include duplicate publishing where the same or very similar results have been published more than once in different papers, which would be excluded after application of inclusion/exclusion criteria.										

8 **Table 2: ‘Coverage of treatment’ systematic literature search for systematic reviews**

**Literature Search Summary Form**	
**Date:** 12/09/2012	
**1. Research question defined**	
Do cancer care coordinators improve coverage of treatment for eligible patients?	
P (population) = patients with cancer	
I (intervention/exposure and setting) = nurse-led care coordination intervention	
C (Comparisons) = baseline care with no care coordinator roles in place	
O (outcome) = improved coverage of treatment for eligible patients	
T (timeframe) = following cancer diagnosis	
**2. Defined Inclusion and Exclusion Criteria**	
Inclusion	Exclusion
Nurse-led patient-level coordination intervention	Systems-level coordination intervention
Hospital based intervention	Community based intervention
Intervention matches our care coordinator intervention specification i.e. face to face contact with patient	Solely telephone-based interventions or other non-face to face patient contact interventions.
**Systematic reviews**	
All systematic reviews relevant to the research question published from 1946 onwards	Opinion pieces, non-systematic reviews
All other specific inclusion and exclusion criteria as defined above	
**3. Explicit search strategy**	
Keywords	Medical Subject Headings (MeSH terms)
coordinat* OR care coordinat* OR patient navigat* OR nurse case manag* OR clinical nurse specialist OR nurse specialist OR nurse practitioner OR case manage* OR key worker OR liaison officer	case management OR nurse clinicians OR nurse practitioners
cancer	Neoplasms
Treatment OR therapy OR surgery OR chemotherapy	therapeutics OR general surgery OR colorectal surgery OR drug therapy
Coverage OR access	
Limits	
English language. Humans. Reviews (maximises specificity)	
**4. Sources used**	
Medline	Cochrane library
DARE and HTA databases	Ministry of Health Publications
**Economic papers**	
NHS EED	
**5. Summary of search**	

**Systematic reviews**	
Number of records retrieved from databases	317
Number of additional records received from other sources	1
Total number of records retrieved after removal of duplicates	318
Number of records excluded by relevance screening	293
Number of articles excluded by exclusion criteria:	
○ Unsuitable article type, e.g. non-systematic review or editorial	
○ Unsuitable study population to address our research question	
○ Unsuitable intervention/exposure to address our research question	
○ Unsuitable outcome to address our research question	24
○ Updated or repetition of other similar review by same authors	
○ Other (specify)	
Number of articles excluded by critical appraisal	0
Total number of articles retained for inclusion	1

Search Results Table: MASTER											
Database	Search string	No. of papers									
		Initial no. retrieved	Duplicates[a]	Total no. excl duplicates	Relevance screening of records		Application of Inclusion/Exclusion Criteria to paper		Critical appraisal (final selection)		
					Exclude	Retain	Exclude	Retain	Exclude	**Final**	
**Systematic reviews**											
Medline 1946 to present (02/08/12)	11. coordinat*.mp. OR care coordinat*.mp. OR patient navigat*.mp. OR nurse case manag*.mp. OR clinical nurse specialist.mp. OR nurse practitioner.mp. OR nurse specialist.mp. OR case manage*.mp. OR key worker.mp OR liaison officer.mp. OR Case Management/cl, ec, og [Classification, Economics, Organization & Administration] OR Nurse Clinicians/cl, ec, ed, og, st, sd [Classification, Economics, Education, Organization & Administration, Standards, Supply & Distribution OR Nurse Practitioners/cl, ec, ed, st [Classification, Economics, Education, Standards] [137656]<br>12. cancer.mp. or Neoplasms/ [987792]<br>13. Treatment.mp. or Therapeutics/ OR therapy.mp. OR surgery or *general Surgery/ or *colorectal surgery OR chemotherapy.mp. or Drug Therapy/ [4108650]<br>14. Coverage or access [193319]<br>15. 1 and 2 and 3 and 4 [130]<br>16. limit 5 to (English language and humans) [116]										

	17. limit 6 to “reviews (maximises specificity)” [0]									
	18. limit 6 to "reviews (best balance of sensitivity and specificity)" [0]									
	19. limit 6 to "review articles" [18]<br>20. remove duplicates from 9 [0]	18	0	18	13	5	5	0	0	**0**
NZ MoH (05/09/12)	Manual search: cancer care coordinators	286	0	286	267	19	19	0	0	**0**
Cochrane Library: Cochrane Database of Systematic Reviews, DARE, HTA Database and NHS EED 1946 -2012 (05/09/12)	14. (coordinat*) or (care coordinat*) or (patient navigation*) or (nurse case manage*) or (clinical nurse specialist) or (nurse specialist) or (nurse practitioner*) or (case manage*) or (key worker) or (liaison officer) [7135] *[Title, Abstract of Keywords]*<br>15. MeSH descriptor **Case Management,** this term only or MeSH descriptor **Nurse Clinicians,** this term only or MeSH descriptor **Nurse Practitioners,** this term only [969]<br>16. #1 or #2 [7211]<br>17. (cancer)[4203] *[Title, Abstract of Keywords]*<br>18. MeSH descriptor **Neoplasms,** this term only [4061]<br>19. #4 or #5 [47900]<br>20. (treatment) or (therapy) or (surgery) or (chemotherapy) [30017] *[Title, Abstract of Keywords]*<br>21. MeSH descriptor **Therapeutics**, this term only or MeSH descriptor **General Surgery**, this term only or MeSH descriptor **Drug Therapy**, this term only [740]<br>22. #7 or #8 [435146]<br>23. (coverage) or (access) [360] *[Title, Abstract of*	13 (1 review, 11 trials, 1 methods study)	0	13	13	0	0	0	0	**0**

	Keywords]<br>24. #3 and #6 and #9 and #10 [13]									
Snowballin g		0	0	0	0	0	0	0	0	**0**
Information Foraging (google scholar and google)	*Goodwin et al*	1	0	1	0	1	0	1	0	**1**
**Total**		**318**	**0**	**318**	**293**	**25**	**24**	**1**	**0**	**1**

9 **Table 3: 'Quality of life' systematic literature search for systematic reviews**

**Literature Search Summary Form**	
**Date:** 12/09/2012	
**1. Research question defined**	
Do cancer care coordinators improve quality of life for patients?	
P (population) = patients with cancer	
I (intervention/exposure and setting) = nurse-led care coordination intervention	
C (Comparisons) = baseline care with no care coordinator roles in place	
O (outcome) = improved patients' quality of life	
T (timeframe) = following cancer diagnosis	
**2. Defined Inclusion and Exclusion Criteria**	
<u>Inclusion</u>	<u>Exclusion</u>
Nurse-led patient-level coordination intervention	Systems-level coordination intervention
Hospital based intervention	Community based intervention
Intervention matches our care coordinator intervention specification i.e. face to face contact with patient	Solely telephone-based interventions or other non-face to face patient contact interventions.
Studies that measure quality of life on an internationally recognised scale	
**Systematic reviews**	
All systematic reviews relevant to the research question published from 1946 onwards	Opinion pieces, non-systematic reviews
All other specific inclusion and exclusion criteria as defined above	
**3. Explicit search strategy**	
<u>Keywords</u>	<u>Medical Subject Headings (MeSH terms)</u>
coordinat* OR care coordinat* OR patient navigat* OR nurse case manag* OR clinical nurse specialist OR nurse specialist OR nurse practitioner OR case manage* OR key worker OR liaison officer	case management OR nurse clinicians OR nurse practitioners
Cancer	Neoplasms
Quality of life	
<u>Limits</u>	
English language. Humans. Reviews (maximises specificity)	
**4. Sources used**	
Medline	Cochrane library
DARE and HTA databases	Ministry of Health Publications
**Economic papers**	
NHS EED	
**5. Summary of search**	

**Systematic reviews**	
Number of records retrieved from databases	381
Number of additional records received from other sources	22
Total number of records retrieved after removal of duplicates	403
Number of records excluded by relevance screening	364
Number of articles excluded by exclusion criteria:	35
o Unsuitable article type, e.g. non-systematic review or editorial	
o Unsuitable study population to address our research question	
o Unsuitable intervention/exposure to address our research question	
o Unsuitable outcome to address our research question	
o Updated or repetition of other similar review by same authors	
o Other (specify)	
Number of articles excluded by critical appraisal	0
Total number of articles retained for inclusion	4

Search Results Table: MASTER										
Database	Search string	No. of papers								
		Initial no. retrieved	Duplicates[a]	Total no. excl duplicates	Relevance screening of records		Application of Inclusion/Exclusion Criteria to paper		Critical appraisal (final selection)	
					Exclude	Retain	Exclude	Retain	Exclude	**Final**
**Systematic reviews**										
Medline 1946 to present (02/08/12)	21. coordinat*.mp. OR care coordinat*.mp. OR patient navigat*.mp. OR nurse case manag*.mp. OR clinical nurse specialist.mp. OR nurse practitioner.mp. OR nurse specialist.mp. OR case manage*.mp. OR key worker.mp OR liaison officer.mp. OR Case Management/cl, ec, og [Classification, Economics, Organization & Administration] OR Nurse Clinicians/cl, ec, ed, og, st, sd [Classification, Economics, Education, Organization & Administration, Standards, Supply & Distribution OR Nurse Practitioners/cl, ec, ed, st [Classification, Economics, Education, Standards] [137656]<br>22. cancer.mp. or Neoplasms/ [987792]									

	23. quality of life.mp or *"Quality of Life"/ [156298<br>24. 1 and 2 and 3 [243]<br>25. limit 4 to (English language and humans) [212]									
	26. limit 5 to "reviews "reviews (best balance of sensitivity and specificity)"[3]<br>27. remove duplicates from 6 [0]	3	0	3	3	0	0	0	0	**0**
NZ MoH (05/09/12)	Manual search: cancer care coordinators	286	0	286	267	19	19	0	0	**0**
Cochrane Library: Cochrane Database of Systematic Reviews, DARE, HTA Database	25. (coordinat*) or (care coordinat*) or (patient navigation*) or (nurse case manage*) or (clinical nurse specialist) or (nurse specialist) or (nurse practitioner*) or (case manage*) or (key worker) or (liaison officer) [7122] *[Title, Abstract of Keywords]*<br>26. MeSH descriptor **Case Management,** this term only or MeSH descriptor **Nurse Clinicians,** this term only or MeSH descriptor **Nurse Practitioners,** this term only [969]	92 (15 reviews, 72 Trials, 4 Methods studies, 1 Economi c evaluatio	0	92	91	1	1	0	0	**0**

and NHS EED 1946 -2012 (14/09/12)	27. #1 or #2 [7198]<br>28. (cancer)[4198] *[Title, Abstract of Keywords]*<br>29. MeSH descriptor **Neoplasms,** this term only [4058]<br>30. #4 or #5 [47766]<br>31. (quality of life) [3198] *[Title, Abstract of Keywords]*<br>32. MeSH descriptor **Quality of Life**, this term only [11993]<br>33. #7 or #8 [23358]<br>34. #3 and #6 and #9 [92]	n)								
Snowballing and Information Foraging (google scholar and google)		22	0	22	3	19	15	4	0	**4**
**Total**		**403**	**0**	**403**	**364**	**39**	**35**	**4**	**0**	**4**
