# Supplementary material for: Cancer care coordinators in stage III colon cancer: a cost-utility analysis
Source: BMC Health Serv Res. 2015 Aug 5;15:306. doi: 10.1186/s12913-015-0970-5 (PMC4523949; doi:10.1186/s12913-015-0970-5)
Supplement: Additional file 4: — Provides additional details on baseline health system costs by disease state. (PDF 827 kb) [file 12913_2015_970_MOESM4_ESM.pdf]

Additional File 5:
Baseline health system costs by disease state

For 'Cancer care coordinators in Stage III colon cancer: a cost-utility analysis'

Tony Blakely[1‡], Lucie Collinson[1], Giorgi Kvizhinadze[1], Nisha Nair[1], Rachel Foster[1], Elizabeth Dennett[2], Diana Sarfati[1]

[1]Burden of Disease Epidemiology, Equity, and Cost-Effectiveness (BODE[3]) Programme, Department of Public Health, University of Otago Wellington, PO Box 7343, Wellington, New Zealand

[2]Department of Surgery, University of Otago Wellington, PO Box 7343, Wellington, New Zealand

[‡]corresponding author

Contents

Additional File 5: Baseline health system costs by disease state 2

Population health system costs 3

Colon cancer stage III health system costs 4

Chemotherapy costs 6

References 9

Additional File 5: Baseline health system costs by disease state

Baseline health system costs (except receipt of chemotherapy; see below) were determined using data from the Ministry of Health that through a national unique identifier brings together all hospital, outpatient, pharmaceutical and laboratory events, and some primary care event and private hospital event data (so-called HealthTracker costs). This data has become reliable from about 2006. Each event is then assigned a unit cost, making it possible to determine annual, monthly or other costs per person, and type of person (e.g. cancer patient in certain phase of cancer journey). About 80% of all Government Vote Health expenditure can be attributed to separate events by individual New Zealand residents. New Zealand has a capitated funding formula for primary care, so an average cost is assigned to all residents based on their sex, age, ethnicity and deprivation (variables used in the capitation funding formula). Thus the primary care costs are 'expectations' and will (say) underestimate resource use for a cancer patient also seeing their general practitioner more frequently that than the average for someone of the same demographic group.

At the time of writing this paper, private or out of pocket health expenditure has not been included in the baseline health system costs. This will result in an underestimate for primary care and private hospital or physician utilisation. However, it should not be too severe for colon cancer costs which are largely public and hospital-based.

Costs are calculated separately by sex and age – but not by ethnicity and deprivation. There are two reasons for this decision. First, due to different usage of private health care (and out of pocket) by ethnicity and deprivation, any differences by ethnicity and deprivation in costs observed with HealthTracker are likely to be (in part at least) of these unobserved differences at the margin. Second, it is a moot point whether current differences in health service utilisations by ethnicity and deprivation are appropriate or fair.

The remainder of this file is in three sections:

- Population health system costs. That is health system costs of an average New Zealander by sex and age, regardless of disease status. We use these costs for two purposes: for health system costs beyond the eight year cure time for colon cancer (i.e. unrelated future health system costs); to assist calculating excess health system costs due to colon cancer.
- Excess colon cancer health system costs.
- Costs of receipt of chemotherapy.

Population health system costs

Two datasets were created: one for costs and person time occurring not in the last six months of life, and one for its complement of costs and person time in the last six months of life. This follows the recommendations of van Baal to specify costs near the end of life separately[1] , and as described in the BODE[3] protocol[2]. For each dataset, averaged health system costs per person-month for the whole population were calculated for all cross-classified strata of sex by age (5-year age-groups) by ethnicity (Māori, non-Māori) by deprivation (three categories) by calendar year (2007, 08, 09, 10, 11). Gamma regression was then used to generate smoothed estimates of health system costs. For reasons explained above, ethnicity and deprivation were not included as covariates in this model, although the input data was disaggregated by these strata. Each age group was assigned its mid-point age in years, minus 62.5 so as to centre age on the 60-64 year age group. The dependent variable itself was the logarithm of average cost per person-month in each strata. As the input data to the regressions was grouped, the regressions were weighted by the total number of people contributing person-time. The estimated costs per person month are shown in Table 1 below, and the regression coefficients given in footnotes.

Table 1: Population health system baseline costs ($NZ) per person-month, by sex and age, and by whether in the last six months of life or not. Adults aged 45 years and older only.

Age	Not in last six months of life		In last six months of life	
	Males	Females	Males	Females
45-49 yrs.	110	124	1070	1296
50-54 yrs.	136	153	1148	1391
55-59 yrs.	168	189	1248	1511
60-64 yrs.	208	234	1369	1658
65-69 yrs.	255	287	1514	1834
70-74 yrs.	307	345	1682	2037
75-79 yrs.	359	404	1873	2268
80-84 yrs.	404	454	2085	2525
85-89 yrs.	433	488	2314	2803
90-94 yrs.	440	495	2554	3094

Gamma regression model coefficients (s.e.).

- Not in last six months of life: Intercept 5.372 (7.572e-02), age 4.201e-02 (2.814e-03), males 1.183e-01 (5.979e-02), year 4.172e-02 (2.112e-02), age squared 2.015e-04 (1.452e-04). age cubed 1.228e-05 (2.223e-06)
- Last 6 month of life: Intercept 7.425 (2.657e-02), age 1.944e-02 (1.117e-03), males 1.916e-01 (2.059e-02), year 5.941e-03 (7.636e-03), age squared 1.486e-04 (2.584e-05), age cubed 3.456e-06 (7.367e-07).

Colon cancer stage III health system costs

The HealthTracker data was restricted to people diagnosed with SEER regional extent of disease stage colon cancer – a reasonable match with stage III. A period window of July 2006 to June 2010 was used. Cancer patients diagnosed since 1998 were included, meaning that there was a full range of times since diagnosis (up to the eight year statistical cure time). The data was cross-classified as above, but rather than using 'not within 6 months of death' and 'within 6 months of death' the cancer disease model categories were used: diagnosis and treatment (DT), remission (R), pre-terminal (PT) and terminal (T). Thus all person-time within nine months of diagnosis contributed to the average DT costs, and likewise all costs in the last month of life for those dying of colon cancer contributed to T and within the fourth to second to last month of life if dying of colon cancer contributed to PT. Regarding R costs, they are any residual time from the 10th month post diagnosis to the 96th month (8 years) if a survivor, or from the 10th month post-diagnosis to the 5th (inclusive) to last month of life if dying from colon cancer.

Within each stratum, the observed average cost per month was calculated (top panel, Table 2). Then the 'excess cost' was calculated by subtracting the matching population costs for the population 'not in the last six months of life' (Table 1 above). Any negative values were replaced with $1 per person month (i.e. due to the population cost by chance being greater than any of the cancer strata costs (due to sparse data), which occurred in one instance). These excess costs were then submitted to a gamma regression model to calculate smoothed excess costs by sex by age by clinical phase. Forced main effects included: sex; age (continuous variable using mid-point of each category, centred on 62.5 years), and cancer state (DT, R, PT, and T). Age (centred) squared and an interaction of age with cancer state, were included. The regression-based estimates are shown in the bottom panel of Table 2, and the regression equation coefficients and standard errors are shown in Table 3.

Table 2: Observed total (averaged over 2006/07 to 2009/10) and regression estimated excess costs per person month for stage III colon cancer

	**Males**				**Females**			
**Age**	**DT**	**R**	**PT**	**T**	**DT**	**R**	**PT**	**T**
Observed total average costs per person month								
45-49 yrs.	5254	1000	6031	5715	5242	409	2039	138193
50-54 yrs.	4105	533	6215	15733	4414	477	8426	6448
55-59 yrs.	4419	437	3756	14463	3778	353	9871	12889
60-64 yrs.	4811	606	3479	10655	4115	304	3788	8792
65-69 yrs.	4733	621	4466	12277	3942	179	6499	15511
70-74 yrs.	4475	440	4798	11125	4054	238	3701	20690
75-79 yrs.	3913	745	3148	12744	3475	51	15205	20783
80-84 yrs.	3695	515	2054	17668	3211	114	2969	15058
85-89 yrs.	3770	414	1983	21700	2909	186	2946	17181
90-94 yrs.	2754	662	2102	30254	2241	337	504	36690
Gamma regression modelled excess cancer costs								
45-49 yrs.	7293	689	9846	18386	6121	579	8264	15433
50-54 yrs.	6004	515	7574	16554	5040	433	6357	13895
55-59 yrs.	5118	399	6032	15432	4296	335	5063	12954
60-64 yrs.	4517	320	4975	14896	3792	268	4176	12504
65-69 yrs.	4128	265	4248	14888	3465	223	3566	12497
70-74 yrs.	3906	228	3756	15407	3279	191	3153	12932
75-79 yrs.	3827	203	3439	16509	3213	170	2886	13857
80-84 yrs.	3883	187	3260	18317	3259	157	2736	15375
85-89 yrs.	4079	178	3199	21042	3424	149	2686	17662
90-94 yrs.	4436	176	3252	25029	3724	147	2729	21008

Table 3: Gamma regression output for model of excess costs

	Estimate	Std.error	P-value
Intercept	8.24	0.12	<0.01
Females (males referent)	0.18	0.09	0.05
Remission (R) †	-2.65	0.13	<0.01
Pre-terminal (PT) †	0.10	0.28	0.73
Terminal (T) †	1.19	0.29	<0.01
Age	-0.021	0.01	0.03
Age squared	0.00070	0.00034	0.04
Age*R	-0.019	0.010	0.05
Age*PT	-0.014	0.019	0.49
Age*T	0.018	0.020	0.37

† Referent category DT

Beyond the cure time of 96 months (8 years), survivors are assigned the average HealthTracker cost per month for someone of the same sex and age group, i.e. those in Table 1. That is, we include unrelated health care costs in the model beyond the colon cancer disease.

Chemotherapy costs

It was not possible to accurately identify, and determine baseline costs, for people receiving chemotherapy from HealthTracker. Therefore, we undertook a bottom-up costing of chemotherapy as follows: cost of chemotherapy itself; cost of staff time and out-patient's attendances. We chose the FOLFOX4 chemotherapy regimen (see Box 1)[3] as:

- this is a commonly prescribed regimen in New Zealand for colon cancer patients stage III along with other FOLFOX regimens.
- it is recommend by New Zealand Guidelines Group[4]
- it concords with the treatment regimen from in the literature used for intervention input parameters as described in Table 1 of the main paper.

We calculated the costs for the FOLFOX4 regimen for the total six month cost and per cycle. Each cycle lasts two days and is every two weeks so there are 12 cycles overall. The acquisition cost of the drugs was sought from the Pharmaceutical Schedule August 2011. The outpatient chemotherapy costs were from National Pricing Outpatient Purchase Unit for DHBs (2011). The number of

outpatient oncology appointments with an oncologist was provided by a local key informant (consultant oncologist) as 6-12 (every 2-4 weeks ;patients are usually seen by an oncologist at the start of every cycle but there is a moving trend to seeing patients every other cycle). We chose to cost six outpatient appointments with an oncologist assuming that each appointment at the start of each new cycle would be shorter in length than an average oncology appointment costed by the National Pricing Outpatient Purchase Unit for DHBs (2011).

We then recalculated the DT costs for months 4 to 9 (inclusive) such that the average was maintained (using the baseline proportion of people receiving chemotherapy), but costs for those people receiving chemotherapy were $17811.78 greater (per patient) than those not receiving chemotherapy.

We did not include knock on costs of complications secondary to chemotherapy as the incidence of these is low. The incidence of febrile neutropenia with FOLFOX4 is 1.8%, the incidence of gastrointestinal adverse effects is low and the incidence of grade 3 sensory neuropathy was 12.4 % during treatment, decreasing to 1.1 % at one year of follow-up[3]. In addition, there is minimal treatment available for sensory neuropathy which gave us further reason not to cost it.

Box 1: Chemotherapy treatment outline FOLFOX4

Treatment Outline FOLFOX 4 [3]

Oxaliplatin 85mg/m^2 IV infusion over 2 hours - **day 1**

Calcium folinate (folinic acid) 200 mg/m^2 IV infusion over 2 hours - **days 1 & 2**

5-Fluorouracil 400mg/m^2 IV slow push - **days 1 & 2**

5-Fluorouracil 600 mg/m^2 IV infusion over 22 hours via Folfusor device – **days 1 & 2**

Table 4: Baseline costs for chemotherapy FOLFOX4 regimen per patient

Acquisition cost of drugs									
**Item***	**Mean dose of pharmaceutical agent for adult cancer patient**	**No. of units per day**		**Total no. units**	**Unit cost****	**Cost per day**		**Total cost (12 cycles)**	**Source and comments**
		**Day 1**	**Day 2**	**Per cycle**		**Day 1**	**Day 2**		
Oxaliplatin (Eloxatin)	85mgx1.8m^2 = 153mg	153mg	0mg	153mg	$165 (Inj 50mg = $55.00, Inj 100mg = $110.00)	$165.00	$0	$165 x 12 = $1980.00	Pharmaceutical Schedule August 2011
Folinic acid (leucovorin, calcium folinate)	200 x 1.8m^2 = 360mg 360x2	360mg	360mg	720mg	$39.75 (Inj 300mg = $30, Inj 100mg = $9.75)	$39.75	$39.75	$79.50 x 12 = $954.00	Pharmaceutical Schedule August 2011
Fluorouracil (5-FU) IV slow push	400 x 1.8m^2 =720mg	720mg	720mg	1440mg	$7.50 (Inj 50mg per ml, 20ml = $7.50, i.e. 1000mg)	$7.50	$7.50	$15 x 12 = $180.00	Pharmaceutical Schedule August 2011
Fluorouracil (5-FU) IV infusion	600 x 1.8m^2 = 1080	1080mg	1080mg	2160mg	$13.55 (Inj 25mg per ml, 100ml = $13.55 i.e. 2500mg)	$13.55	$13.55	$27.10 x 12 = $325.20	Pharmaceutical Schedule August 2011
**Total drug acquisition cost**								$3439.20	
**Cost of staff time and out-patient's attendances to receive chemotherapy**									
**Outpatient chemotherapy**	An attendance where the purpose is to receive intravenous chemotherapy. Includes all pharmaceuticals administered during the attendance excluding chemotherapy drug cost recovery from Sector Services. Includes day case treatment.				$493.83	$493.83	$493.83	$987.66 x 12 **=** $11851.92 per cycle	National Pricing Outpatient Purchase Unit for DHBs (2011)
**Outpatient appointments with oncologist during chemotherapy and follow-up**									
**Oncology outpatient attendance**					$420.11 per appointment			6 x $420.11= $2520.66	National Pricing Outpatient Purchase Unit for DHBs (2011)
**Total cost of drugs, plus outpatient attendances, plus appointments with oncologist =** $3439.20 + 11851.92 +$2520.66 **=** $17811.78 (per patient)									

*IV fluids given with the chemo agent (e.g. 5% glucose) are not costed separately as they are included in the outpatient chemotherapy per attendance cost.
**We have rounded up the dose of each chemotherapy agent to the nearest vial size as suggested by Dooley et al whose work showed by doing this had minimal impact on the cost of chemotherapy agents.[5].

References

1. van Baal P, Feenstra T, Polder J, Hoogenveen R, Brouwer W: **Economic evaluation and the postponement of health care costs.** *Health economics* 2011, **20**(4):432-445.
2. Blakely T, Foster R, Wilson N: **Burden of Disease Epidemiology, Equity and Cost-Effectiveness (BODE3) Study Protocol** In: *Burden of Disease Epidemiology, Equity and Cost-Effectiveness Programme.* Wellington: University of Otago Wellington 2012.
3. Andre T, Boni C, Mounedji-Boudiaf L, Navarro M, Tabernero J, Hickish T, Topham C, Zaninelli M, Clingan P, Bridgewater J, Tabah-Fisch I, de Gramont A: **Oxaliplatin, fluorouracil, and leucovorin as adjuvant treatment for colon cancer**. *N Engl J Med* 2004, **350**(23):2343-2351.
4. New Zealand Guidelines Group: **Management of early colorectal cancer. Evidence-based Best Practice Guideline.** In. Wellington Ministry of Health; 2011.
5. Pollock N, Langtont E, Stowell K, Simpson C, McDonnell N: **Safe duration of postoperative monitoring for malignant hyperthermia susceptible patients**. *Anaesthesia and intensive care* 2004, **32**(4):502-509.
