# Supplementary material for: Cancer care coordinators in stage III colon cancer: a cost-utility analysis
Source: BMC Health Serv Res. 2015 Aug 5;15:306. doi: 10.1186/s12913-015-0970-5 (PMC4523949; doi:10.1186/s12913-015-0970-5)
Supplement: Additional file 5: — Contains the event pathway and details of costing the baseline and intervention. (PDF 752 kb) [file 12913_2015_970_MOESM5_ESM.pdf]

Additional File 4:
Event pathway and costing of baseline and intervention
For 'Cancer care coordinators in Stage III colon cancer: a cost-utility analysis'

Tony Blakely[1‡], Lucie Collinson[1], Giorgi Kvizhinadze[1], Nisha Nair[1], Rachel Foster[1], Elizabeth Dennett[2], Diana Sarfati[1]

[1]Burden of Disease Epidemiology, Equity, and Cost-Effectiveness (BODE[3]) Programme, Department of Public Health, University of Otago Wellington, PO Box 7343, Wellington, New Zealand

[2]Department of Surgery, University of Otago Wellington, PO Box 7343, Wellington, New Zealand

[‡]corresponding author

Contents

ADDITIONAL FILE 4: EVENT PATHWAY AND COSTING OF BASELINE AND INTERVENTION 2

Event pathway 2

Calculation of intervention costs 5

Resource Use Survey 5

Additional allied health referrals 9

Uncertainty 10

REFERENCES 13

Additional File 4: Event pathway and costing of baseline and intervention

There are two types of cost to ascribe: routine health system costs, and intervention costs. Health system costs are those that routinely occur given the health state a patient is in, regardless of 'direct' costs of the intervention; we derive them from a dataset of all New Zealand residents linked to costs per event and by type of person (so-called HealthTracker data, described in general elsewhere[1], and more specifically for this economic evaluation in Additional File 5. Intervention costs as we model them are incremental costs, whereby costs for the CCC intervention (e.g. salaries and costs arising from direct consequences of the intervention such as increased use of allied professionals) are summed, then the costs of the comparator (i.e. the costs of current cancer care services in the absence of cancer care coordinators) are subtracted. It is these incremental costs that are the focus of this file.

Event pathway

The event pathway is in two sections and shown below in Figure 1 and Figure 2. The clinical nurse specialist (CNS) role is adapted from a description provided by Maria Stapleton, Clinical Nurse Specialist Colorectal Care, MidCentral District Health Board (DHB). Expert advice was provided by Dr Elizabeth Dennett, Colorectal surgeon, CCDHB and Dr Andrew Simpson, Medical Oncologist, CCDHB.

Figure 1: Cancer care coordinator intervention pathway for colon cancer stage III from provisional diagnosis to surgery

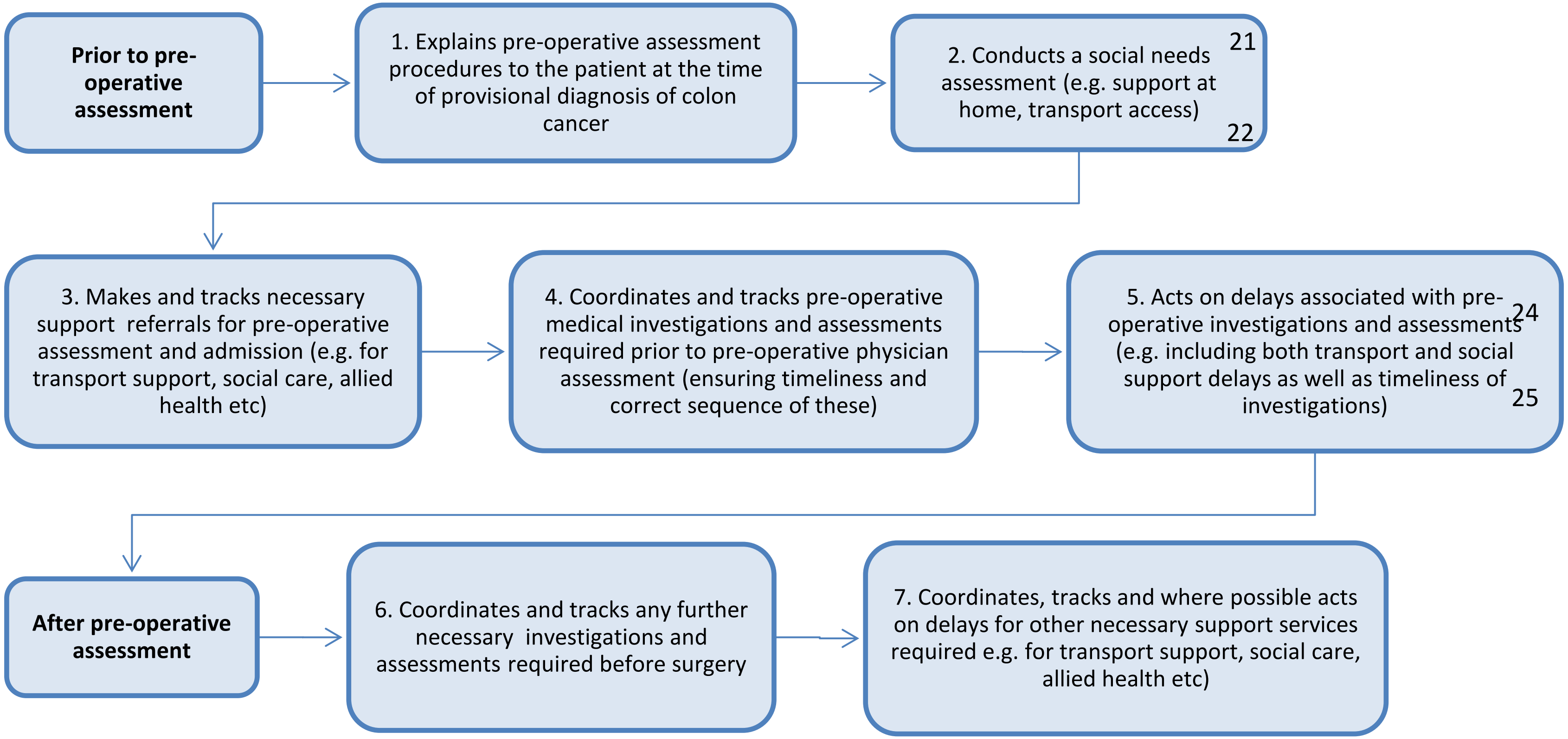

Figure 2: Cancer care coordinator intervention pathway for colon cancer stage III from surgery to chemotherapy

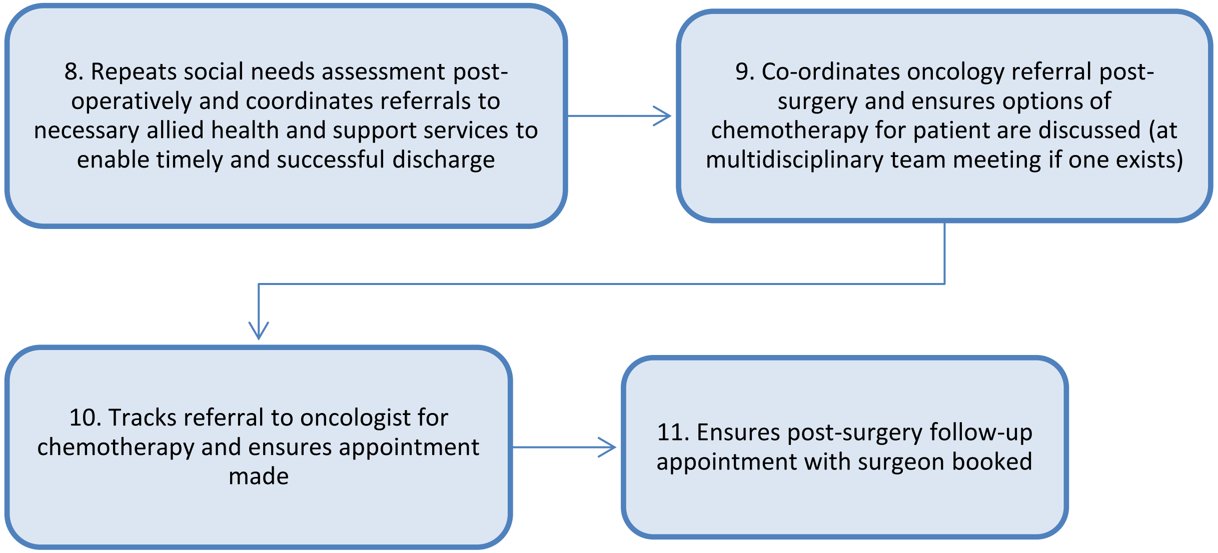

Calculation of intervention costs

Our initial calculation was largely a ‘desktop’ exercise by the authors (RF and LC) using the above event pathway, available datasets in New Zealand on price per resource unit, and a resource use survey. The latter survey of health professionals (including a variety of nursing roles, house surgeons, registrars and consultants; n =16) was to estimate time spent on “coordinating” activities, We considered care in the time periods from diagnosis to surgery (steps 1-7) and surgery to initiation of chemotherapy (steps 8-11). Step 10 (tracking oncology referral) in the intervention pathway was not currently being conducted in any hospital that we surveyed; we estimated a time of 10 minutes for this activity. We didn’t include care during chemotherapy in the intervention as this is already coordinated by community cancer nurses in New Zealand.

Resource Use Survey

Methods

The resource use survey was designed so that the questions corresponded with each step in the event pathway. Survey participants were asked which member(s) of staff (if any) provided each service for colon cancer patients stage III, the time it took to provide this service per patient per member of staff (for the average patient and the range for all patients they see). Of note, we attempted to elicit experts’ estimates of the range about the average patient time (i.e. equivalent to the standard deviation about the mean), but this was too challenging for participants.

Health care workers from three hospitals were asked to take part in the survey; Wellington Hospital and Kenepuru Community Hospital where no specified CCC role currently exists, and Palmerston North Hospital where a specified nursing role matching parts of our CCC intervention is in place. Health care workers invited to participate were identified as likely to be carrying out at least one step in the event pathway either alone or alongside other staff.

In the two hospitals where no specific CCC exists twenty-four members of staff were invited to participate in the survey with twelve completing it (all from Wellington Hospital). Health care workers with the following roles were asked to participate: colorectal consultant (1), registrars (5), house surgeons (6), colorectal surgery clinic nurse (n=5), stomal therapy nurse (n=2), pre-assessment nurse (n=3), patient flow coordinator (n=1) and medical secretary (n=1). The following roles completed the survey: colorectal consultant (1), registrars (3), house surgeons (2), colorectal surgery clinic nurse (n=1), stomal therapy nurse (n=2), pre-assessment nurse (n=3). The survey was completed face to face individually with five participants, face to face collectively with three participants – providing one estimate between them for each question asked and over the phone individually with four participants.

In the hospital where parts of the CCC intervention were being provided four nursing staff were asked to participate with 100% response rate. The survey was completed individually by one colorectal surgery clinic nurse and collectively by three colorectal cancer nurses. We were unable to meet with these participants face to face, but were in correspondence on the telephone and email to discuss queries with regards to the survey.

Analysis

For each activity, we calculated the average of the estimates given, using the midpoint of the range if a point estimate wasn't given.

From the data collected in the survey we determined the average amount of time spent by each type of personnel on each activity. The total time for each phase (provisional diagnosis to surgery; surgery to initiation of chemotherapy) was calculated separately for hospitals where there was ad hoc coordination of patient care (comparator) and those where specified roles existed that carried out some or all of the tasks defined in our CCC programme (intervention).

The cost per hour of activity was then calculated based on an average salary for each type of personnel, assuming that a CCC would be a CNS[2-4]. As described in Additional File 1 the CCC requires the skillset and experience of a CNS in order to know when to seek specialist input and in order to be able to answer patients' questions with regards to their management.

In line with an opportunity cost approach, we determined that each hour spent on coordinating activity was equivalent to the loss of an hour spent on clinical activities directly related to the care of an individual patient; we will refer to the latter as "patient-related activity time". We define "patient-related activity time" as including all contact with and care of the patient, phone calls, emails and other administrative tasks related directly to the care of the individual patient. It excludes lunch, morning and afternoon tea breaks, training, meetings (other than those related to care of individual patients such as multidisciplinary team meetings (MDTs)), and administration related to the professional's organisation rather than individual patients. We applied the salary only over the periods of the individual's work time that were potentially patient-related activity time; we excluded public holidays, annual leave and sick leave, and assumed that 62.5% of each day was patient-related activity time (i.e. 5 hours of an 8 hour day). Overheads of 50% were added to the salaries.

Table 1: Resource use survey results and costs (average per patient) for the time periods: diagnosis to surgery, and; post-surgery to initiation of chemotherapy. Costs include salary and overheads

**Time period**	**Number of units (minutes)**	**Cost per unit ($)[a]**	**Total cost ($)**
**Comparator**			
Provisional Diagnosis to surgery			
• Registered nurse	101.88	$1.36	$138.56
• Clinical nurse specialist	15.00	$1.76	$26.40
• Registrar	25.63	$1.69	$43.31
• House surgeon	15.42	$1.32	$20.35
• Consultant	14.29	$4.03	$57.59
• Ward Clerk	0	$0.89	$0.00
Subtotal	*172.20*		*$286.18*
Post-surgery to initiation of chemotherapy			

• Registered nurse	28.33	$1.36	$38.53
• Clinical nurse specialist	15.00	$1.76	$26.40
• Registrar	8.33	$1.69	$14.08
• House surgeon	24.38	$1.32	$32.18
• Consultant	6.25	$4.03	$25.19
• Ward Clerk	5.00	$0.89	$4.45
Subtotal	87.29		*$140.82*
Total	***259.49***		***$427.00***
**Intervention**			
Diagnosis to surgery			
• Registered nurse	0	$1.36	$0.00
• Clinical nurse specialist	167.50	$1.76	$294.80
• Registrar	7.50	$1.69	$12.68
• House surgeon	0	$1.32	$0.00
• Consultant	9.50	$4.03	$38.29
• Ward Clerk	5	$0.89	$4.45
Subtotal	*189.50*		*$350.20*
Post-surgery to initiation of chemotherapy			
• Registered nurse	0	$1.36	$0.00
• Clinical nurse specialist	67.5	$1.76	$118.80
• Registrar	7.50	$1.69	$12.68
• House surgeon	7.50	$1.32	$9.90
• Consultant	0	$4.03	$0.00
• Ward Clerk	5.00	$0.89	$4.45
Subtotal	87.50		*$145.82*
Total	***277.00***		***$496.02***
**Incremental (i.e. Intervention minus comparator)**			
Diagnosis to surgery			
• Registered nurse	-101.88	$1.36	-$138.56
• Clinical nurse specialist	152.50	$1.76	$268.40
• Registrar	-18.13	$1.69	-$30.64
• House surgeon	-15.42	$1.32	-$20.35
• Consultant	-4.79	$4.03	-$19.30
• Ward Clerk	5	$0.89	$4.45
Subtotal	*17.30*		*$64.03*
Post-surgery to initiation of chemotherapy			
• Registered nurse	-28.33	$1.36	-$38.53
• Clinical nurse specialist	52.50	$1.76	$92.40
• Registrar	-0.83	$1.69	-$1.40
• House surgeon	-16.88	$1.32	-$22.28
• Consultant	-6.25	$4.03	-$25.19
• Ward Clerk	0	$0.89	$0.00
Subtotal	*0.21*		*$5.00*
Total	***17.51***		***$69.03***

a Based on the following annual salaries:[2-4]

- House surgeon/officer (category D, year 2): $74,557
- Registrar (category D, year 5) $95,631
- Consultant (grade 10) $173,349
- Registered nurse (step 5) $61,362
- Clinical nurse specialist (grade 4, step 2) $79,347
- Ward Clerk $40,000

Overheads of 50% were then added to the above salaries.

Additional allied health referrals

Additional allied health referrals associated with the CCC programme were based on data from the New South Wales programme: 83% of those with coordinated care were referred to an allied health professional such as a psychologist or social worker compared to 42 per cent with standard care) [5].

The national price for an outpatient purchase unit for a social worker in New Zealand is $163.86 per contact. Our estimate (based on information from a local key informant) is that those who are referred would have six contacts.

Incremental probability of allied health = 0.83-0.42 = 0.41

Cost for those referred = 6*163.86 = $983.16

Incremental cost averaged across all patients = 0.41* $983.61 = $403.10

We also estimate that CCCs would refer an additional 50% of colon cancer patients to dieticians at $115.89 per contact, with each two contacts per referral.

Probability of dietician = 0.5 (incremental)

Cost for those referred = 2*115.89 = $231.78

Cost averaged across all patients = 0.5 * $231.78= $115.89

Both of these additional costs would be incurred over time, however for simplicity we assumed that each patient in the intervention arm incurred these costs (we also conducted a scenario analysis without additional allied health costs – see main paper).

In the absence of information on variance, a standard deviation of 10% was assumed for allied health costs.

Uncertainty

The data given in the resource use survey are interpreted as "on average patients required x time for this activity (the average point estimate), but some may require only y time (low end of range) and some may require as much as z time (upper end of range)." Thus, the variation is across individuals; as stated above, respondents found it too challenging to attempt to estimate variation in expected or mean values at the population-level.

Variation across the population will be less than that across individuals. To estimate population variation, we need to make an assumption regarding how much less that variation will be.

The standard formula for standard error of the mean (population variation) is:

$$SE = \frac{upper\ CI - lower\ CI}{2 * 1.96}$$

If we assume the population variation is quarter that of the individual variation, and that the range values approximate the 95% confidence intervals, the population standard error (SE) is calculated as follows:

$$SE = \frac{(upper\ range - lower\ range) * 0.25}{2 * 1.96}$$

The population variance is simply the SE squared.

In calculating costs, it was assumed that there is uncertainty around the number of resource units (e.g. minutes of care from the resource use survey), but no uncertainty around the price per unit

(salaries per minute). Thus scenario 3 for uncertainty of costs was applied, as per the BODE^3 Protocol on Direct Costing of Interventions[6].

The value of each item (i.e. the time assigned to each type of activity step according to the type of personnel performing the activity) was the average from the resource survey. Each respondent contributed data to a number of items, thus correlation would be expected – that is, an individual is likely to consistently underestimate or overestimate each item they provide an estimate for. Correlation both between items within each arm (intervention or comparator) and between arms was assumed to be 0.25. We used standard statistical formulas to estimate the variance about the sum of the individual items (with their variance as estimated above; normal distribution assumed), to give the total coordination cost and standard deviation estimate about the mean in <u>both</u> the intervention and comparator arms per patient:

- Provisional diagnosis to surgery cost:
    - CCC intervention arm: Estimated mean $350.20, s.d. $17.43
    - Comparator (or business-as-usual) arm: Estimated mean $286.16, s.d. $9.57
- Surgery to chemotherapy cost:
    - CCC intervention arm: Estimated mean $145.82, s.d. $7.53
    - Comparator (or business-as-usual) arm: Estimated mean $140.82, s.d. $4.79

Finally, we calculated the incremental cost and its variance and standard deviation (again assuming a correlation of 0.25 and standard statistical formula for the variance of differences):

- Provisional diagnosis to surgery incremental cost: $64.03 (s.d. $17.66)
- Surgery to start of chemotherapy incremental cost: $5.00 (s.d. $7.85).

Note that an assumption of correlations of 0 or 0.5 between items within each arm, and between the arms, had little impact on the estimated s.d. about the incremental cost. For example, the above

estimate of \$64.03 would have an s.d. of \$15.33 under the 0 correlation assumption, and \$17.56 under the 0.5 assumption. This is because the correlations in summing items within each arm largely off-set the correlation between the arms when working out the variance. Accordingly, we do not present scenario analyses about varying correlations.

Also note the use of a normal distribution above the central limit theorem supports such a use, although for parameterised costs within just one arm we would err to still using a gamma distribution. However, here we model the incremental cost, which is the comparator cost subtracted from intervention cost. It is plausible that such costs might be negative (i.e. cost saving). A gamma distribution does not allow negative costs, and now that we are subtracting one arm's estimate from another, the normal distribution assumption has a stronger basis.

Regarding additional allied health professional costs (i.e. dietician and social worker), a similar procedure to above was used, except that an expected cost in the intervention arm only was estimated, and therefore a gamma distribution assumed.

References

1. Blakely T, Foster R, Wilson N: **Burden of Disease Epidemiology, Equity and Cost-Effectiveness (BODE3) Study Protocol** In: *Burden of Disease Epidemiology, Equity and Cost-Effectiveness Programme.* Wellington: University of Otago Wellington 2012.
2. Resident Doctors' Association, District Health Boards: **Multi Employer Collective Agreement and Terms of Settlement 28 March 2011 to 31 March 2012**. In.; 2011.
3. New Zealand Nurses Organisation, District Health Boards: **Nursing and Midwifery Multi Employer Collective Agreement 1 April 2010 to 30 Sep 2011**. In.; 2011.
4. New Zealand District Health Boards, Association of Salaried Medical Specialists: **Senior Medical and Dental Officers Collective Agreement 1 July 2007 until 30 April 2010: Variation of Terms 9 September 2010**. In.; 2010.
5. Cancer Institute NSW: **Evaluation of the role of Cancer Care Coordinator. Sydney: Cancer Institute NSW**. In. New South Wales Cancer Institute NSW; 2011.
6. Foster R, Blakely T, Wilson N, al e: **Protocol for Direct Costing of Health Sector Interventions for Economic Modelling (Including Event Pathways)**. In. Wellington: Department of Public Health, University of Otago; 2012.
